# Orthogonal matrix factorization enables integrative analysis of multiple RNA binding proteins

**DOI:** 10.1093/bioinformatics/btw003

**Published:** 2016-01-18

**Authors:** Martin Stražar, Marinka Žitnik, Blaž Zupan, Jernej Ule, Tomaž Curk

**Affiliations:** ^1^University of Ljubljana, Faculty of Computer and Information Science, Ljubljana, SI 1000, Slovenia; ^2^Department of Molecular and Human Genetics, Baylor College of Medicine, Houston, TX 77030, USA; ^3^Department of Molecular Neuroscience, UCL Institute of Neurology, Queen Square, London WC1N 3BG, UK

## Abstract

**Motivation:** RNA binding proteins (RBPs) play important roles in post-transcriptional control of gene expression, including splicing, transport, polyadenylation and RNA stability. To model protein–RNA interactions by considering all available sources of information, it is necessary to integrate the rapidly growing RBP experimental data with the latest genome annotation, gene function, RNA sequence and structure. Such integration is possible by matrix factorization, where current approaches have an undesired tendency to identify only a small number of the strongest patterns with overlapping features. Because protein–RNA interactions are orchestrated by multiple factors, methods that identify discriminative patterns of varying strengths are needed.

**Results:** We have developed an integrative orthogonality-regularized nonnegative matrix factorization (iONMF) to integrate multiple data sources and discover non-overlapping, class-specific RNA binding patterns of varying strengths. The orthogonality constraint halves the effective size of the factor model and outperforms other NMF models in predicting RBP interaction sites on RNA. We have integrated the largest data compendium to date, which includes 31 CLIP experiments on 19 RBPs involved in splicing (such as hnRNPs, U2AF2, ELAVL1, TDP-43 and FUS) and processing of 3’UTR (Ago, IGF2BP). We show that the integration of multiple data sources improves the predictive accuracy of retrieval of RNA binding sites. In our study the key predictive factors of protein–RNA interactions were the position of RNA structure and sequence motifs, RBP co-binding and gene region type. We report on a number of protein-specific patterns, many of which are consistent with experimentally determined properties of RBPs.

**Availability and implementation:** The iONMF implementation and example datasets are available at https://github.com/mstrazar/ionmf.

**Contact**: tomaz.curk@fri.uni-lj.si

**Supplementary information:** Supplementary data are available at *Bioinformatics* online.

## 1 Introduction

RNA-binding proteins (RBPs) play a major role in the control of gene expression. Misregulation of RBPs is associated with diseases such as fragile X syndrome, neurologic disorders and cancer (Darnell, 2013). Our understanding of protein–RNA interaction has been greatly improved by the use of genomic methods such as individual-nucleotide resolution UV crosslinking and immunoprecipitation (iCLIP), which identifies RBP crosslinking sites on a genome-wide scale. Past iCLIP studies have shown that RBPs bind and regulate a large number of transcripts. Computational analysis and prediction of these interactions is therefore critical to gain a comprehensive understanding of RBP functions (Dieterich *et al.*, 2013).

Current approaches to model protein–RNA interactions focus on individual data sources and require precise structural knowledge of the involved proteins (Cirillo *et al.*, 2013; Klus *et al.*, 2014; Puton *et al.*, 2012). They rarely exploit other available omics data. General approaches such as Bayesian networks (Zhang *et al.*, 2010), Hidden Markov models (Zhang *et al.*, 2013), or SVMs (Livi *et al.*, 2014), have been applied to model protein–RNA interactions using multiple data sources. However, their application was limited to individual RBPs and the importance of only a limited number of features governing protein–RNA interaction were highlighted, such as single degenerate motifs. The lack of presented features is partly due to the difficulties associated with interpretation of inferred models. We have developed a modeling technique based on multiple matrix factorization that is capable of integrating data sources for multiple RBPs. The generated models are both accurate and interpretable. New biological knowledge can be gained by exploring the identified combinatorial effects among various features of data sources that define patterns of protein–RNA binding sites on RNA.

Nonnegative matrix factorization (NMF) methods have been extensively applied in machine learning for clustering, community detection, classification, etc. (Carmona-Saez *et al.*, 2006; Gao *et al.*, 2005; Wang *et al.*, 2013). The classic NMF algorithm (Lee *et al.*, 2001) finds an approximation of a data matrix that is described as a product of two or more matrices with lower ranks – a factor model. An advantage of NMF is the interpretable, parts-based representation of patterns present in the data. This is due to the latent factors being constrained to non-negative values, which can then be combined in an additive way to approximate the original data.

Integrative NMF approaches provide biologically meaningful results in various bioinformatics applications. For example, NMF was used to integrate multiple matrices with a common dimension and to discover miRNA and gene regulatory modules (Zhang *et al.*, 2011), or to discover modules of genes, miRNA targets and DNA methylation markers in cancer patients (Zhang *et al.*, 2012).

Various improvements of the NMF algorithm have been suggested to obtain more comprehensive models. The sparsity of factor models improves the interpretability and modularity of projections. Sparsity is achieved by including *L*_1_ norm constraints on the model coefficients. Alternatively, the $L_{1}/L_{2}$ norm ratio of the resulting projection can be explicitly tuned (Hoyer, 2004), which produces sparser solutions, but does not guarantee modularity. Other methods constrain the basis vectors to convex sets (Ding *et al.*, 2010; Guan *et al.*, 2012). The mentioned methods, however, do not focus on modular decompositions where samples and features do not overlap within clusters. This is a substantial drawback when classes are discriminated by multiple patterns of varying strengths. This phenomenon is common in the domain of protein–RNA interactions, as strong patterns, e.g. U-rich tracts present in binding sites (König *et al.*, 2010) of many proteins, may occlude weaker signals that discriminate between proteins. A possible solution is to require the basis vectors found by NMF to be orthogonal. One such example is the orthogonality-constrained NMF (Ding *et al.*, 2006) that assumes an initial orthogonal model, e.g. obtained by *k*-means clustering, which may bias the final model.

We have developed an integrative, *orthogonality-regularized* nonnegative matrix factorization method (iONMF). The method finds modular projections of data matrices, where data instances are assigned to *modules* described by non-overlapping features. In a supervised setting, orthogonality regularization prevents multicollinearity (Chatterjee *et al.*, 2015; Nicodemus *et al.*, 2009), where a feature vector can be expressed as a linear combination of a subset of remaining feature vectors. This is important, as RBPs differ in specificity and their binding target patterns differ in number and strength. We applied iONMF on the largest integrative analysis in the number of RBPs and different data sources used. The analysis included a compendium of 31 published CLIP experiments on 19 RBPs and other genomic data sources to predict RBP crosslinking sites at a nucleotide-resolution. We discovered discriminative patterns across different data sources and learned a comprehensive model of protein–RNA interaction for each of the 19 RBPs. We visualized the discovered patterns and used them to cluster RBPs into functionally related groups. Our results demonstrate the applicability of iONMF for fast and accurate prediction of RBP target sites on a genome-wide scale.

## 2 Methods

### 2.1 Data sources and sampling

We analyzed data on 19 proteins with one or more experimental replicates, 31 experiments in total. Three experimental protocols were used to determine protein–RNA crosslinking sites: **PAR-CLIP**: Ago/EIF2C1-4, IGF2BP1-3, PUM2 (Hafner *et al.*, 2010); Ago2-MNase, ELAVL1, ELAVL1-MNase, ELAVL1A (Kishore *et al.*, 2011); ESWR1, FUS, TAF15 (Hoell *et al.*, 2011); MOV10 (Sievers *et al.*, 2012); **iCLIP**: hnRNPC, U2AF2 (Zarnack *et al.*, 2013); hnRNPC (König *et al.*, 2010); hnRNPL, hnRNPL-like (Rossbach *et al.*, 2014); Nsun2 (Hussain *et al.*, 2013); TDP-43 (Tollervey *et al.*, 2011); TIA1, TIAL1 (Wang *et al.*, 2010); **CLIP-SEQ/HITS-CLIP**: Ago2, ELAVL1 (Kishore *et al.*, 2011); eIF4AIII (Saulière *et al.*, 2012); SRSF1 (Sanford *et al.*, 2009); Ago2 (Boudreau *et al.*, 2014). When clusters of interaction sites were reported (e.g. PAR-CLIP, Ago/EIF2C1-4), we treated all positions within clusters as interacting. Technical or biological replicates of the same selected RBP were grouped. We use the term *experimental group* to refer to one such group; see Supplementary Table S1. Data were obtained from servers iCount (http://icount.biolab.si) and DoRiNA (Anders *et al.*, 2012).

#### 2.1.1 Sampling of crosslinked sites

**Fig. 1.**
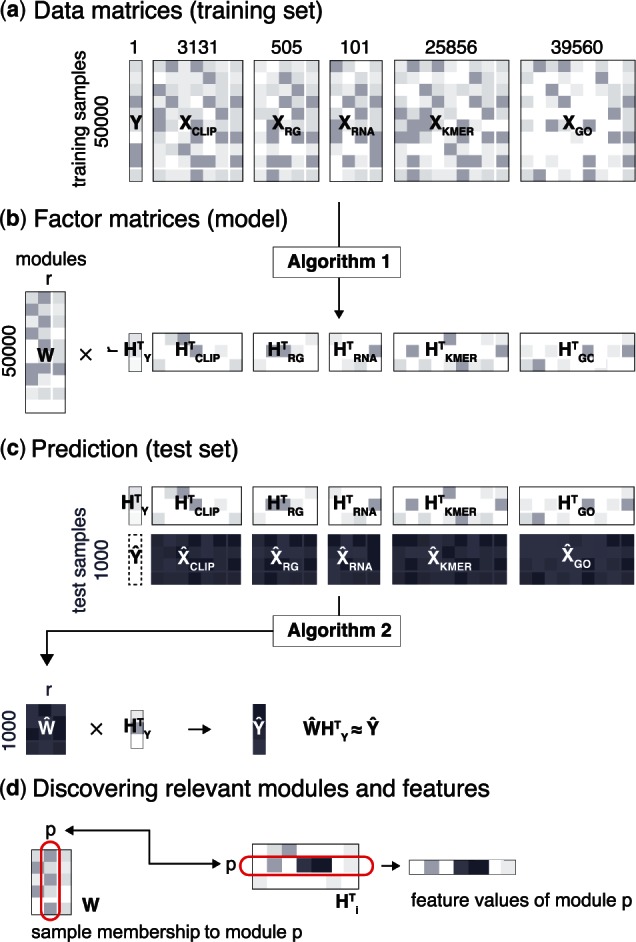
Overview of the analysis procedure. (**a**) Target column vector Y and other data sources $X_{i}$ used for training. (**b**) iONMF factorization (Algorithm 1) approximates the data sources with a factor model (common coefficient matrix **W** and a basis matrix $H_{i}$ for each data source). (**c**) Prediction of test samples (Algorithm 2) uses the basis matrices $H_{i}$ and other data sources $X_{i}$, $H_{Y}$ and test sample data $\hat{X_{i}}$ to estimate the coefficient matrix $\hat{W}$ and predict $\hat{Y}$ (**d**) Samples are assigned to modules based on rows in **W**. Row *p* in $H_{i}^{T}$ describes the characteristic feature values of each module (*p*)

In each experiment, we first identified up to 100 000 nucleotide positions with the highest cDNA counts. These were used as a pool of positive examples of protein–RNA crosslinking nucleotides. Among positions, which were less than 15 nucleotides apart, we considered only the positions with the highest cDNA count and ignored all others within a 15-nucleotide distance, as suggested in the original iCLIP publication (König *et al.*, 2010). With this step we prevented the sampling of consecutive genomic positions, which are very similar in composition. Among neighboring positions with the same cDNA count, one was randomly picked. To reduce processing time and ensure comparable results among experiments, we sampled up to 10 000 positions. For proteins with less than 20 000 identified crosslinking sites, we randomly split the sites into training and test sets. Including more positive examples did not change the predictive performance of our models (Supplementary Fig. S1). Negative examples of protein–RNA interaction sites were sites within genes that were not detected as interacting in any experiment (in total 1 293 531 975 sites). Among them we sampled at least 40 000 positions and used them as negative examples of crosslinking nucleotides. In total, the training set included 50 000 positions (Fig. 1a,b) which were uniformly drawn from the genome.

The test set (Fig. 1c) was constructed similarly. To assure a clear separation between the two sets, positions for the test set were sampled only from genes not used for training. The total number of detected clusters and crosslink sites are listed in Supplementary Table S1.

#### 2.1.2 Data matrices

Each training data matrix included up to 50 000 rows. For experiments performed on a smaller number of rows, the number is explicitly stated. Each row represents a nucleotide position described using various data sources. The number of columns varies for each data source:

**Y: selected RBP experiment CLIP cDNA count**, 50000×1. protein–RNA cDNA counts are reported for a selected RBP experiment on the current nucleotide position (in row), resulting in 1 column. This column was used for model fitting and to evaluate the predictive performance.

**X_CLIP:_ other proteins CLIP cDNA counts**, 50000×3131. For each of the remaining (up to 30) RBP experiments that were not from the same group as the selected RBP experiment, the cDNA counts at positions [−50..50] relative to the current nucleotide (in row) were reported as 1 for nonzero cDNA counts or 0 otherwise, resulting in up to 30×101=3030 columns. By explicitly ignoring experiments within the same replicate group (shown in Supplementary Table S1), we assured that replicate information was not used in evaluation.

**X_RG:_ Region type**, 50000×505. Each position [−50..50] relative to the current nucleotide (in row) was assigned into five types of gene regions, as determined by the Ensembl annotation version ensembl69 for human genome assembly hg19 (Hubbard *et al.*, 2002): exon, intron, 5’UTR, 3’UTR, CDS, resulting in 5 × 101 = 505 columns. Precise boundaries of regions near crosslink sites could thus be captured. For each gene region type, its presence at a relative position was indicated with a binary value.

**X_RNA:_ RNA secondary structure**, 50000×101. Sequences at positions [−50..50] relative to the current nucleotide (in row) were processed with RNAfold software (Denman, 1993), resulting in probabilities of double-stranded RNA secondary structure at each of 101 relative positions.

**X_KMER:_ RNA k-mers**, 50000×25856. Positions [−50..50] relative to the current nucleotide (in row) were scanned for the presence of RNA *k*-mers, with *k* = 4 in all experiments. The presence of a *k*-mer at a relative position was indicated with a binary value.

**X_GO:_ Gene annotation**. Genomic positions within known genes were annotated with Gene Ontology (Ashburner *et al.*, 2000) terms for *goa_human*, 39560 terms (revision 5758736 from 2014-10-06).

Test data matrices $\left(\hat{Y},{\hat{X}}_{\text{CLIP}},{\hat{X}}_{\text{RG}},{\hat{X}}_{\text{RNA}},{\hat{X}}_{\text{KMER}},{\hat{X}}_{\text{GO}}\right)$ have the same structure, but they described a different subset of positions not included in the training set.

### 2.2 Analysis overview

A *factor model* of the training set was inferred with iONMF (Fig. 1a). The resulting coefficient matrix **W** determined the grouping of nucleotides into *r* modules, based on similarity across all data sources. A *module* is defined as characteristic features in each data source, represented as a column vector in matrices $H_{i}$, corresponding to: co-binding to the same targets as other RBPs (**H**_CLIP_), RNA k-mers (**H**_KMER_), surrounding region types (**H**_RG_), RNA secondary structure (**H**_RNA_) and Gene Ontology terms (**H**_GO_) (Fig. 1b).

Having learned the coefficient and basis matrices with iONMF, we estimated the crosslinking affinity of the samples in the test set for all RBP experiments (columns) in the target **Y** column (Fig. 1d). The test samples were projected into the learned low dimensional space spanned by **W**, using all additional data sources $\left({\hat{X}}_{\text{CLIP}},{\hat{X}}_{\text{RG}},{\hat{X}}_{\text{RNA}},{\hat{X}}_{\text{KMER}},{\hat{X}}_{\text{GO}}\right)$ that describe the test set. Each step is described in detail in the following.

Threefold cross-validation was used to estimate the predictive accuracy. Internal cross-validation (80%/20% sampling, repeated three times) on the training set was used to select best hyperparameter values.

### 2.3 Integrative orthogonality-regularized nonnegative matrix factorization (iONMF)

Data sources are represented with matrices $X_{i}$, with *m* rows representing samples and *n_i_* columns representing features from each data source. The classic NMF approximates each $X_{i}\in R^{m\times n_{i}}$ with a factor model - a product of a common coefficient matrix $W\in R^{m\times r}$ and data source-specific basis matrices $H_{i}\in R^{n_{i}\times r}$, where the rank $r<<\text{min}(m,\sum_{i}n_{i})$. Empirically, a low approximation error model is achieved when patterns repeat across multiple data sources.

Each sample (row) is assigned to one or more of the *r* modules, which is reflected by the learned weights in **W**. Features relevant to each module are reflected in corresponding basis vectors in $H_{i}$ (rows in $H_{i}^{T}$). Highly correlated positions are assigned to common modules, depending on their similarity across all data sources $X_{i}$.

Non-overlapping features relevant to each module are obtained by imposing orthogonality on the basis vectors. Therefore, we developed integrative, Orthogonality-regularized NMF (iONMF), which employs the scalarization approach for orthogonality regularization, optimizing the trade-off between orthogonality and approximation error. The iONMF model is learned by solving the following optimization problem. Given multiple data matrices $X_{i}$, minimize the cost function:

(1) $$J(W,H_{i})=\sum_{i=1}^{N}\left(||X_{i}-WH_{i}^{T}|{|}_{F}^{2}+\alpha ||H_{i}^{T}H_{i}-I|{|}_{F}^{2}\right)$$

such that $W,H_{i}\geq 0$ and **I** is the identity matrix. The first term in the sum represents approximation error and second term the orthogonality of column vectors in $H_{i}$, where the trade-off is controlled by hyperparameter *α*. The problem is non-convex and can be solved by (projected) gradient descent, alternating-least squares (Lee *et al.*, 2001), multiplicative update rules (Lin, 2007) or second order gradient methods (Zdunek *et al.*, 2006). Due to computational efficiency and a principled way to include orthogonality and non-negativity constraints, we propose an iterative multiplicative update Algorithm 1, which is an instance of gradient descent with variable learning rate. The algorithm starts by initializing the values in **W** and $H_{i}$ randomly, uniformly distributed on [0,1], and updates them with the following rules until convergence:

(2) $$W=W○\sqrt{\frac{\sum_{i}X_{i}H_{i}}{\sum_{i}WH_{i}^{T}H_{i}}}$$

(3) $$H_{i}=H_{i}○\sqrt{\frac{X_{i}^{T}W+\alpha H_{i}}{H_{i}W^{T}W+2\alpha H_{i}H_{i}^{T}H_{i}}}$$

where ○ represents the element-wise (Hadamard) product. The treatment of the target column vector Y and its corresponding basis matrix $H_{Y}$ is equivalent to other data sources except for the orthogonality constraints, which are not used since $H_{Y}$ consists only of a single column. Further discussion on the choice of algorithm, derivation of update rules, relation to gradient descent, and convergence to a stationary point are shown in Supplementary Section S2. The algorithm is run for multiple random initializations and the factor model with the lowest approximation error is selected.

Algorithm 1. iONMF on multiple data matrices.

**Input:** data matrices $X_{i}$, target vector **Y**, approximation rank *r*, orthogonality-approximation trade-off *α*

**Output:** coefficient matrix **W**, basis matrices $H_{i},H_{Y}$

1: $W∼U{\left[0,1\right]}^{m\times r}$

2: **for each** i: $H_{i}∼U{\left[0,1\right]}^{n_{i}\times r}$

3: $H_{Y}∼U{\left[0,1\right]}^{r\times 1}$

4: **until** convergence:

5: $W=W○\sqrt{\frac{\sum_{i}X_{i}H_{i}+YH_{Y}}{\sum_{i}WH_{i}^{T}H_{i}+WH_{Y}^{T}H_{Y}}}$

6: **for each** i: $H_{i}=H_{i}○\sqrt{\frac{X_{i}^{T}W+\alpha H_{i}}{H_{i}W^{T}W+2\alpha H_{i}H_{i}^{T}H_{i}}}$

7: $H_{Y}=H_{Y}○\sqrt{\frac{Y^{T}W}{H_{Y}W^{T}W}}$

### 2.4 Predicting crosslinked sites

Running matrix factorization on large datasets requires a trade-off between the number of samples considered and the computational time. A common assumption when applying NMF for prediction is that all objects in the domain, including the test samples, are used in learning (Yoo *et al.*, 2009). Cold-start approaches (Zhou *et al.*, 2011) or regression on the obtained factors (Joshi *et al.*, 2010) can be used to predict test samples. Alternatively, non-negative least-squares optimization is used to approximate the coefficient matrix values from available matrices describing new samples (Zitnik *et al.*, 2015). These methods suffer from substantial drawbacks, namely the requirements of additional functions/classifiers, high computational cost, or lack of interpretability.

Our *model-based* approach reuses the learned low-rank matrices to predict test samples. Having learned the iONMF model of cDNA counts on a smaller subset of genomic positions, we therefore used it to predict cDNA counts for all other genomic positions (matrix $\hat{Y}$). Predicted counts were in turn used to classify positions as crosslinked or not crosslinked.

Algorithm 2 uses the learned factor model to address the problems mentioned above and is a special case of Algorithm 1. Given the learned and fixed basis matrices $H_{i}$, $H_{Y}$, and new samples with known $\hat{X_{i}}$, we use the update rule 2 to first solve for $\hat{W}$ and then predict $\hat{Y}=\hat{W}H_{Y}^{T}$.

Algorithm 2. Prediction of test samples.

**Input:** data matrices $\hat{X_{i}}$, target source basis matrix $H_{y}$, basis matrices $H_{i}$

**Output:** coefficient matrix $\hat{W}$, prediction $\hat{Y}$

1: $\hat{W}∼U{\left[0,1\right]}^{m\times r}$

2: **until** convergence:

3: $\hat{W}=\hat{W}○\sqrt{\frac{\sum_{i}\hat{X_{i}}H_{i}}{\sum_{i}\hat{W}H_{i}^{T}H_{i}}}$

4: $\hat{Y}=\hat{W}H_{Y}^{T}$

### 2.5 Discovering relevant modules and features

The obtained coefficient matrix **W** is used to assign data samples (in rows) to specific modules (in columns); see Figure 1d. The values of **W** are determined based on all $X_{i}$ and define the modules, while individual $H_{i}$ are determined based only on the corresponding data sources $X_{i}$.

Proposed methods include assigning the sample to the module with maximum row value or restricting the assignment to only one module (Brunet *et al.*, 2004). Alternatively, the ability to assign samples to multiple modules may be desired. One such approach, developed by Zhang *et al.* (2012), converts each entry in the coefficient matrix to the corresponding column-wise z-score. Samples are assigned to modules where the corresponding z-score exceeds a predefined threshold (empirically set to 1.96). For each of the *r* modules, we obtain a count *C_r_* of how many samples are related to the corresponding module.

Since we are interested in modules describing the positive data samples (crosslinked sites) for a particular protein in question, we sorted the modules on descending value of *C_r_*. The corresponding (column) vectors of matrices $H_{i}$ were then examined to discover the relevant features of each data source that determine protein crosslinking and binding (see Fig. 1d).

To extract complex RNA motifs of arbitrary length from the k-mer frequency and positional information encoded in the learned factor model ($H_{\text{KMER}}$), we used an approach similar to Hutchins *et al.* (2008). In contrast, we consider all data sources to identify the sequence motifs and their positional distribution associated with protein binding. For details on the algorithm, see Supplementary Section S7.6 and Supplementary Algorithm 2.

## 3 Results and discussion

### 3.1 Predictive performance

We compared iONMF against factorization methods using various constrained optimization techniques: NMF with multiplicative updates (Zhang *et al.*, 2012); Sparse NMF (SNMF) using alternating non-negative least-squares with *L*_1_ regularization (Kim *et al.*, 2007); NMF-QNO using quasi-newton optimization and *L*_1_ regularization (Zdunek *et al.*, 2006).

For each RBP experiment, the methods were run on the training set for three different initializations. The model with the lowest approximation error was used for prediction of the test set with Algorithm 2 (adapted for NMF, SNMF and NMF-QNO to assume fixed $H_{i}$). Samples were projected into the low dimensional space $\hat{W}$ to predict $\hat{Y}$. Empirically, algorithms converged in less than 100 iterations (change in cost function value $<10^{-6}$). The factorization rank was set to *r* = 10 for all methods.

We used cross-validation on the training set of 30 000 positions to choose hyperparameters: orthogonality regularization *α* (iONMF), *L*_1_ regularization (SNMF, NMF-QNO). Hyperparameters were sampled from range $\left[10^{-3}..10^{3}\right]$. The reported predictive performances are measured with the Area under ROC curve (AUC) on the prediction on the independent hold-out test set of size 1000. Prediction using iONMF resulted in highest AUC in 24 out of 31 cases. iONMF, NMF and NMF-QNO methods consistently outperformed SNMF. The critical distance diagram (Demšar, 2006) shown in Table 1 confirms the statistical significance (*P* < 0.05) of the observed differences in ranks of classifiers over multiple datasets, confirming the feasibility of orthogonality as a way to induce discriminative and parsimonious factor models.

**Table 1.** Predictive performance measured with area under ROC curve (AUC) on the hold-out test sets for the evaluated matrix factorization methods

Protein	iONMF	NMF	SNMF	QNO	Protein	iONMF	NMF	SNMF	QNO
[1] Ago/EIF.	0.89	**0.89**	0.85	0.87	[17] hnRNPC	**0.97**	0.96	0.48	0.70
[2] Ago2M.	**0.71**	0.69	0.66	0.69	[18] hnRNPL	0.74	0.73	0.70	**0.77**
[3] Ago2	0.81	0.81	0.76	**0.83**	[19] hnRNPL	**0.66**	0.62	0.56	0.61
[4] Ago2	**0.84**	0.82	0.79	0.82	[20] hnRNPLl.	**0.69**	0.67	0.63	0.68
[5] Ago2	**0.73**	0.71	0.65	0.66	[21] MOV10	**0.96**	**0.96**	0.89	0.92
[6] eIF4AIII	0.92	0.91	0.78	**0.95**	[22] Nsun2	0.81	0.80	0.69	**0.82**
[7] eIF4AIII	0.93	**0.93**	0.67	0.64	[23] PUM2	**0.93**	0.92	0.86	0.89
[8] ELAVL1	**0.91**	0.89	0.71	0.80	[24] QKI	**0.84**	0.77	0.52	0.62
[9] ELAVL1M.	**0.71**	0.70	0.68	0.70	[25] SRSF1	**0.85**	**0.85**	0.73	0.86
[10] ELAVL1A	**0.94**	0.93	0.91	0.92	[26] TAF15	**0.91**	0.89	0.82	**0.91**
[11] ELAVL1	0.95	0.94	0.90	**0.95**	[27] TDP-43	**0.84**	0.78	0.45	0.57
[12] ESWR1	**0.87**	0.85	0.80	0.85	[28] TIA1	**0.93**	0.92	0.86	0.90
[13] FUS	**0.81**	0.73	0.55	0.65	[29] TIAL1	**0.87**	0.86	0.73	0.85
[14] Mut FUS	**0.96**	0.95	0.91	0.94	[30] U2AF2	**0.82**	0.74	0.61	0.70
[15] IGF2.1-3	**0.93**	0.92	0.89	0.91	[31] U2AF2	**0.80**	0.74	0.60	0.74
[16] hnRNPC	**0.95**	0.93	0.45	0.63					
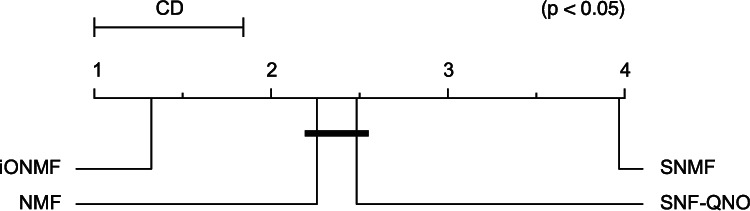									

A critical distance diagram of average ranks is shown above.

We compared iONMF with GraphProt and RNAContext on the same dataset used by Maticzka *et al.* (2014). iONMF performed best on 13 out of 24 RBP experiments (AUC=0.907±0.041). Critical difference diagrams show equivalent performance of iONMF and GraphProt (AUC=0.887 ± 0.079), while RNAContext was significantly lower (AUC=0.830 ± 0.119), see Supplementary Section S3 for details.

Next, we investigated the influence of hyperparameter *α* on sparseness and average angle between vectors in $H_{i}$. Higher angle values indicate greater orthogonality of the respective vectors. Supplementary Figure S2 shows the average AUC across all 31 experiments with varying *α*. As *α* is increased, sparseness (Hoyer, 2004) increases from 0.46 to 0.79, effectively halving the number of non-zero model parameters. Also, the pairwise angle between vectors in $H_{i}$ increases from 65^∘^ to 90^∘^. Even for extreme values of *α*, AUC changes from 0.88 to 0.83. We compared the feature vectors found by all factorization methods in more detail, see Supplementary Section S4.2.

A single run of iONMF model training for a single RBP experiment runs for 12 minutes on a 2.5 GHz CPU. With a trained model, prediction is performed at a rate 300 000 positions/h.

### 3.2 Estimating the importance of combinations of data sources

**Fig. 2.**
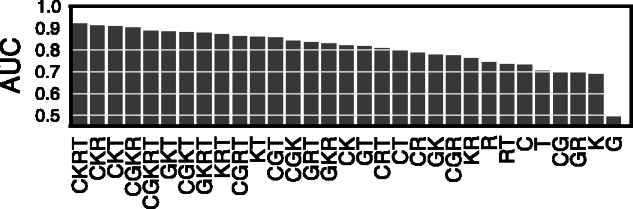
Average AUCs over 31 experiments, for all combinations of data sources: CLIP experiments (C; **X**_CLIP_), RNA k-mers (K; **X**_KMER_), region type (T; **X**_RG_), Gene Ontology terms (G; **X**_GO_) and RNAfold structure prediction (R; **X**_RNA_)

To estimate the importance of data sources, we measured AUC as stated above, for each possible subset of data sources and each selected RBP experiment (Supplementary Tables S7–S9 for AUC of individual experiments). We then calculated the average AUC and standard error obtained for each data source subset across all selected RBP experiments, shown in Figure 2 and Supplementary Table S6. To ensure fair comparison, the factorization rank *r* was selected such that the total number of model parameters was approximately equal for each subset of data sources (Supplementary Table S6).

We turn to estimation of the importance or particular data sources. According to AUC, the most informative data source is RNA structure (col. R, average AUC = 0.744 ± 0.024, Fig. 2, Supplementary Table S6). This agrees with previous observations about the importance of particular RNA structure interaction interfaces (Kazan *et al.*, 2010; Li *et al.*, 2010), but may also reflect the need for RNA bases to be single stranded to allow UV crosslinking (Sugimoto *et al.*, 2012). The second most informative data source is information on interaction of other proteins within the same gene region (col. C, average AUC = 0.732 ± 0.018, Fig. 2, Supplementary Table S6). This agrees with combinatorial protein–RNA interactions that compete or cooperate for RNAs binding (Chan *et al.*, 2014; Jens and Rajewsky, 2015), but may also indicate that many RNA nucleotides may have generally increased accessibility and crosslinking efficiency.

The most informative pair of data sources are RNA *k*-mers (K) and type of genomic region (T) with average AUC = 0.860 ± 0.017 (col. KT, Fig. 2, Supplementary Table S6). These features describe the genomic organization and sequence content biases of functional subunits, e.g. exon, intron, UTR and exon-intron boundaries.

The poor performance when using **X**_GO_ alone (col. G, AUC = 0.492 ± 0.008, Fig. 2, Supplementary Table S6) is likely due to sparse and incomplete gene function annotation, which is in great contrast to other data sources that are two orders of magnitude denser (density of **X**_GO_ is  0.01%, while density of **X**_RG_ is  16%). Inclusion of **X**_GO_ into the most informative subset (col. CKRT, AUC = 0.920 ± 0.006, Fig. 2, Supplementary Table S6) does not change the predictive accuracy significantly (col. CGKRT, AUC = 0.886 ± 0.011, Fig. 2, Supplementary Table S6).

The average AUC correlates with the total number of included data sources. The best accuracy was achieved on $\left\{X_{\text{CLIP}},X_{\text{KMER}},X_{\text{RNA}},X_{\text{RG}}\right\}$, col. CKRT. Except for **X**_GO,_ combining two or more data sources resulted in better accuracy than in models obtained on individual data sources, supporting the benefit of data integration. A more detailed, Spearman correlation-based comparison of all data source combinations confirms several binding preferences supported by the literature (Supplementary Section S6 and Supplementary Fig. S5).

### 3.3 Identifying factors associated with RBP binding

Factor models were used to identify features associated with each discovered module. As explained in Section 2.5 and shown in Figure 1d, each module reveals common feature values of crosslinked sites (samples) assigned to the module. These values are reflected in $H_{i}$, one row for each of *r* modules. The identified modules of crosslinked sites with common features were visualized and used to predict functionally relevant protein–RNA interactions. Visualization of a complete set of RBP experiments and the three most relevant feature vectors are shown in Supplementary Section S8.

In the following paragraphs we present the results and provide an explanation for U2AF2 [30], a known splicing factor, where the most informative single data sources are $X_{\text{KMER}}$ (AUC = 0.754), $X_{\text{RG}}$ (AUC = 0.695), $X_{\text{CLIP}}$ (AUC = 0.632), $X_{\text{RNA}}$ (AUC = 0.554), $X_{\text{GO}}$ (AUC = 0.372); see Supplementary Table S9. The most informative data subset is $\left\{X_{\text{CLIP}},X_{\text{KMER}},X_{\text{RNA}},X_{\text{RG}}\right\}$ (AUC = 0.933).

**Fig. 3.**
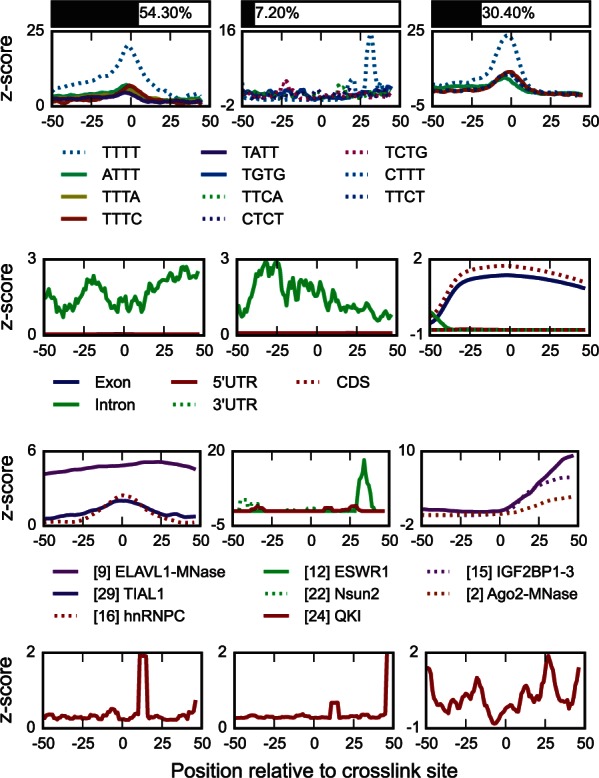
Three modules most associated with positions bound by U2AF2 [30] are shown, top to bottom: $H_{\text{KMER}},H_{\text{RG}},H_{\text{CLIP}},H_{\text{RNA}}$ Top bars show the percentage of nucleotides included in the corresponding module

**RNA secondary structure.** In agreement with U2AF2 being a single-strand RNA binding protein, the probability of double stranded RNA decreases around its crosslinked sites. Features in $H_{\text{RNA}}$ are shown in Figure 3. Hierarchical clustering of feature vectors in **H**_RNA_ are shown in Supplementary Figures S8, S9 and Supplementary Section S8.3.

**RBP co-binding and k-mer composition.** Examining features in $H_{\text{CLIP}}$ , one is able to discover factors associated with binding of individual or groups of RBPs. The features that are common to each module allow us to define hypotheses on cooperative or competitive binding of multiple proteins, which can then be experimentally tested. Figure 3 shows results for U2AF2. It also shows that splicing factor hnRNPC interacts with the same RNA positions as U2AF2. Competition between the two is reported by (Zarnack *et al.*, 2013). The two factors also share similar binding motifs (compare Fig. 3 and k-mers in Supplementary Section S8.1). The relationship is further confirmed by recognition of U-rich motifs, appearing in the corresponding module in $X_{\text{KMER}}$.

Orthogonality regularization provides an advantage in interpretation over NMF. iCLIP and CLIP based methods are subject to a U-rich sequence preference due to UV-C cross-linking. As reported previously (Sugimoto *et al.*, 2012), the detection of U-rich motifs may occur at crosslinks for RBPs not associated with U-rich tracts, such as TDP-43 (Supplementary Fig. S4). The NMF method discovers both U-rich motif and known tandem UG repeats in a single module (column vector in **H**_KMER)_, while iONMF successfully distinguishes the two. Assigning the data samples to corresponding modules (Section 2.5), 41.6% of positive samples are related to UG-rich component, while 80.3% are related to U-tracts (note that the two sets are overlapping). The similarity of proteins based on k-mer composition and co-binding is shown in Supplementary Figures S6, S7, S10 and Supplementary Sections S8.1, S8.4.

**Fig. 4.**
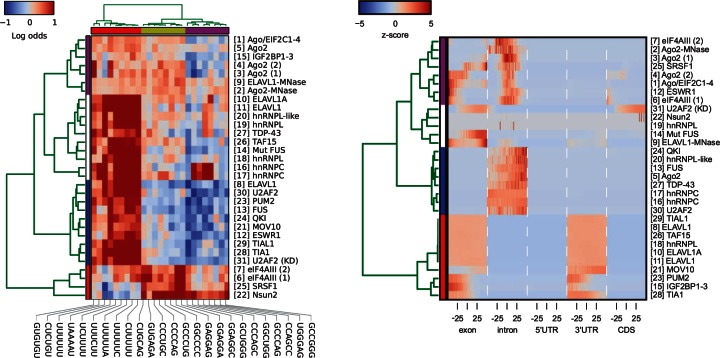
(left) Hierarchical clustering (Ward’s linkage) of proteins and 20 most common complex motifs, estimated from row vectors $H_{\text{KMER}}$obtained with iONMF. Heatmap shows log odds ratios of observed motif probability in sites proximal to crosslinked sites divided by the expected probability (at random positions). Weblogos of motifs are shown in Supplementary Figure S13. (right) Protein similarity based on gene region types row vectors in $H_{\text{RG}}$. For each region type, the interval [-50..50] relative to the crosslinked sites is shown

**Region type.** Figure 3 shows $H_{\text{RG}}$ features for U2AF2. The intron-exon boundary can be seen at ∼30 nucleotides upstream from the crosslinked site. This is expected since U2AF2 is a splicing factor that generally crosslinks to a 3’ splice site (Zarnack *et al.*, 2013). Protein similarity based on region types is shown in Figure 4b, confirming the ability of iONMF feature vectors to cluster the proteins into functionally related groups. Detailed data is shown in Supplementary Figure S11 and individual feature vectors in Supplementary Section S8.2.

**Sequence motif content and positioning.** Figure 3 shows the sequence content and positions of RNA sequence *k*-mers (features in $H_{\text{KMER}}$) for U2AF2. The most associated k-mers are U-rich and are similar to recognition sites of hnRNPC, an experimentally confirmed competitor for the same binding sites (Zarnack *et al.*, 2013). Co-binding of the two can be seen in $H_{\text{CLIP}}$ matrices; see Supplementary Section S8.4.

**Gene annotation**. Gene Ontology terms associated with targets of RBPs are shown in Supplementary Section S8.5 and in Supplementary Figure S12.

### 3.4 Comparison with previously known motifs

We estimated more complex and longer motifs from features in $H_{\text{KMER}}$, as explained in Supplementary Section S7.6. For example, Supplementary Figure S14 shows the top ranked 4-mers from data on PUM2 [23], and the reconstructed complex motif UGUANAUA, using the algorithm described in Supplementary Section S7.6. The identified motif perfectly matches the motif reported for PUM2 in (Hafner *et al.*, 2010). Supplementary Figure S14 also shows that the log probability of motif presence increases significantly in the vicinity of the crosslinked sites compared to the presence in random positions within protein coding genes. This important result shows the partial information in 4-mers can be used to infer longer and complex motifs.

To validate our approach, we compared the identified motifs with motifs obtained from the *in vitro* RNAcompete assay (Ray *et al.*, 2013). We compared motifs of nine RBPs that are included in both studies. Motifs were aligned to minimize the Levenshtein distance (*D*) between motifs derived with the procedure from Supplementary Section S7.6. Ten out of twelve motifs match their reported counterparts with D≤1; see Supplementary Figure S15 for results and visual rendering of the comparison. Moreover, the discovered motif agree in large part to GraphProt (Maticzka *et al.*, 2014).

To identify groups of proteins with similar interaction properties, we performed k-means clustering on binding motif preference. We obtained 10 candidate motifs for each RBP (310 motifs in total; see Supplementary Section S7.6). Using k-means clustering (*k* = 20), we reduced the set to 20 most common complex motifs. For each experiment and for each of the 20 selected motifs, we calculated the log odds ratio of observed versus expected occurrence in positive and negative positions. These values were then used in hierarchical clustering of proteins and motifs; see Figure 4a. For clustering and motif weblogos obtained for *k* = 50, see Supplementary Figure S13.

Four groups of proteins and three groups of motifs can be seen. We found that hnRNP proteins that bind to introns to regulate splicing bind to U-rich motifs (hnRNPs, U2AF2, ELAVL1, TDP-43, TAF15, FUS, QKI), whereas those binding to exons to regulate splicing (SR), spliced mRNA (eIF3E3), or 3 UTR (Ago, IGF2BP) mRNA are GC-rich, in agreement with the fact that introns are U-rich, and exons are GC-rich (Amit *et al.*, 2012; Aznarez *et al.*, 2008). Motifs associated with hnRNPC, e.g. GGCUGG, GCCCAG, CCUGCC, GCCGGG, commonly occur in antisense Alu elements next to the U-tract that directly interacts with hnRNPC (Supplementary Figs S16 and S17). Hence, iONMF can detect common neighboring motifs even if these are not part of the primary binding site.

## 4 Conclusion

Computational approaches already play a crucial role in protein–RNA interaction prediction by aiding experiment planning and interpretation of results. Genome-wide assays of protein–RNA interaction mapping (Castello *et al.*, 2012) has identified close to a thousand human RNA-binding proteins. Data on RNA binding proteins is growing rapidly, emphasizing the need for integrative methods which jointly consider all available data sources.

An interesting finding of our study is that in addition to RNA structure and sequence, the position relative to genomic features (exons, etc.) and CLIP data of other RBPs is informative for predicting binding sites of a specific RBP. Genomic regions are informative as many proteins bind at specific positions relative to these features, e.g. U2AF2 generally binds upstream of exons (Fig. 3). We show that CLIP data are predictive, as subsets of examined RBPs exhibit similar binding patterns (Fig. 4, Supplementary Figs S6–S13 and Supplementary Section S8.4). Importantly, overlap is only seen between a subset of RBPs, but we find no evidence that some sites or features are generally shared across all RBPs. While contribution of non-specific background should be considered, we find it most likely that co-binding profiles result from biologically relevant features. For example, many RBPs bind to similar RNA sequences or structures (Supplementary Figs S6–S9, S13).

Several of the examined RBPs are known to bind similar motifs, such as the U-rich motifs bound by ELAVL, TIA, hnRNPC and U2AF2, which are also detected in our analyses (Fig. 4). Moreover, RBPs may interact at the protein level, either directly or indirectly via co-factors, which could stabilize their binding to proximal RNA sites. Few experimental studies have explored the impact of protein–protein interactions on coordinated RNA binding, but our analyses could be used to explore such potential interactions in the future.

Data integration in iONMF yields improvements in accuracy when compared to state-of-the-art approaches. Orthogonality regularization favors non-overlapping and sparse solutions, which are desired by domain experts, providing class-specific descriptions and model interpretation. The resulting predictions are in strong accordance with a published *in vitro* study and identified a number of promising candidates for further investigation. Together, our experimental findings establish iONMF as the data integration technique of choice where sparse, modular models are desired.

## Supplementary Material

Supplementary Data
