# Supplementary material for: Orthogonal matrix factorization enables integrative analysis of multiple RNA binding proteins
Source: Bioinformatics. 2016 Jan 18;32(10):1527–35. doi: 10.1093/bioinformatics/btw003 (PMC4894278; doi:10.1093/bioinformatics/btw003)
Supplement: Supplementary Data [file supp_32_10_1527__index.html]

Orthogonal matrix factorization enables integrative analysis of multiple RNA binding proteins — Orthogonal matrix factorization enables integrative analysis of multiple RNA binding proteins — Supplementary Data 

# Orthogonal matrix factorization enables integrative analysis of multiple RNA binding proteins

## Supplementary Data

files

- Supplementary Data - pdf file
