# Supplementary material for: Orthogonal matrix factorization enables integrative analysis of multiple RNA binding proteins
Source: Bioinformatics. 2016 Jan 18;32(10):1527–35. doi: 10.1093/bioinformatics/btw003 (PMC4894278; doi:10.1093/bioinformatics/btw003)
Supplement: Supplementary Data [file supp_btw003_supplement_a_1_4.pdf]

Orthogonal matrix factorization enables integrative analysis of multiple RNA binding proteins: Supplementary Material

Martin Stražar [1], Marinka Žitnik[1], Blaž Zupan [1,2], Jernej Ule [3] and Tomaž Curk [1*]

December 11, 2015

Contents

1 Detailed information on analyzed RBP experiments **3**

2 Details on the iONMF algorithm **4**
2.1 Derivation of update rules . . . 4
2.2 Equivalence to gradient descent . . . 5

3 Comparison of iONMF, RNAContext and GraphProt **6**

4 Empirical evaluation of orthogonality-regularized factor models **9**
4.1 Effect of orthogonality on predictive performance and model sparseness . . . 9
4.2 Correlation among feature vectors (predictors) . . . 10
4.3 Comparison of discovered modules . . . 10

5 Details on models learned from subsets of data sources **11**
5.1 Model parameters . . . 11
5.2 Prediction accuracy for data source subsets on individual RBPs . . . 12

6 Importance of individual data sources **15**

7 Clustering of RBPs based on individual data sources **16**
7.1 RNA k-mers ($\mathbf{H}_{\text{KMER}}$) . . . 16
7.2 RNA secondary structure ($\mathbf{H}_{\text{RNA}}$) . . . 17
7.3 Experimental cDNA counts ($\mathbf{H}_{\text{CLIP}}$) . . . 18
7.4 Genomic region types ($\mathbf{H}_{\text{RG}}$) . . . 18
7.5 Gene associations ($\mathbf{H}_{\text{GO}}$) . . . 19
7.6 Discovery of RNA motifs . . . 20
7.7 Sequences of Alu elements bound and regulated by hnRNPC . . . 23

8 Data source feature values **25**
8.1 RNA k-mers ($\mathbf{X}_{\text{KMER}}$) . . . 25
8.2 Region Type ($\mathbf{X}_{\text{RG}}$) . . . 31
8.3 RNAfold ($\mathbf{X}_{\text{RNA}}$) . . . 37
8.4 Experiments ($\mathbf{X}_{\text{CLIP}}$) . . . 43
8.5 Gene associations ($\mathbf{H}_{\text{GO}}$) . . . 50

References **58**

List of Tables

1 Detailed information on individual protein-RNA experimental interaction data used. Total number of sites for each experiment. Experiments describing same protein in different technical or biological replicates are grouped. . . . 3
2 Comparison of AUC scores between iONMF, GraphProt and RNAContext. GraphProt and RNAContext performance values taken from Maticzka *et al.* (2014). Only sequence ($\mathbf{X}_{\text{KMER}}$) and structure ($\mathbf{X}_{\text{RNA}}$) data sources are used by iONMF. . . . 7
3 Comparison of AUC scores between iONMF, GraphProt and RNAContext. GraphProt and RNAContext performance values taken from Maticzka *et al.* (2014). All available data sources are used by iONMF. . . . 7

*to whom correspondence should be addressed

4 Comparison of precision-recall scores between iONMF, GraphProt and RNAContext. GraphProt and RNAContext performance values taken from Maticzka *et al.* (2014). Only sequence ($\mathbf{X}_{\mathrm{KMER}}$) and structure ($\mathbf{X}_{\mathrm{RNA}}$) data sources are used by iONMF. 8
5 Comparison of precision-recall scores between iONMF, GraphProt and RNAContext. GraphProt and RNAContext performance values taken from Maticzka *et al.* (2014). All available data sources are used by iONMF. 8
6 Details on parameter settings for factor models on differet subsets of data sources. 11
7 Area under ROC curve for all combinations of RBP experiments and data sources subsets. 12
8 Area under ROC curve for all combinations of RBP experiments and data sources subsets (continued). . . 13
9 Area under ROC curve for all combinations of RBP experiments and data sources subsets (continued). . . 14
10 Gene Ontology terms, modules and z-scores. 50
11 Gene Ontology terms, modules and z-scores (continued). 51
12 Gene Ontology terms, modules and z-scores (continued). 52
13 Gene Ontology terms, modules and z-scores (continued). 53
14 Gene Ontology terms, modules and z-scores (continued). 54
15 Gene Ontology terms, modules and z-scores (continued). 55
16 Gene Ontology terms, modules and z-scores (continued). 56
17 Gene Ontology terms, modules and z-scores (continued). 57

List of Figures

1 Average performance of iONMF and NMF over 31 RBP experiments, depending on the size of the training set. The test set contains 1000 positions with 20% positives. 9
2 Effect of parameter α on performance (y axis), sparseness (left), and angle of basis vectors $\mathbf{H}_i$ (right) of model obtained with iONMF. 9
3 Comparison of average maximal pairwise correlation between low-rank components (rows in $\mathbf{H}$). 10
4 Comparison of vectors in $\mathbf{H}_{\mathrm{KMER}}$related to cross-links of [27] TDP-43. **left:** Non-regularized NMF. **Middle, right:** Top two relavant components found by iONMF. 10
5 Hierarchical clustering of proteins based on the importance of each individual data source. 15
6 Protein similarity based on RNA k-mer row vectors in $\mathbf{H}_{\mathrm{KMER}}$. Features represent all possible kmers within the interval [-50..50] relative to the cross-link sites, resulting in $101 \times 256 = 25856$ features. To avoid clutter, only the centers and first three nucleotides of the 4-mer are displayed. 16
7 Protein similarity based on RNA k-mer row vectors in $\mathbf{H}_{\mathrm{KMER}}$. K-means clustering is performed on row vectors from top modules for each of the RBP experiments. Ten k-mers closest to the centroid vectors were selected. The z-scores within the intervals [-50..50] nucleotides are displayed. 16
8 Protein similarity based on RNA secondary structure row vectors in $\mathbf{H}_{\mathrm{RNA}}$. Z-scores of features obtained via RNAfold output are proportional to the predicted probability of double-stranded RNA at the particular nucleotide within the [-50..50] interval relative to the cross-link sites. 17
9 Protein similarity based on RNA secondary structure row vectors in $\mathbf{H}_{\mathrm{RNA}}$. Z-scores of features obtained via RNAfold output are proportional to the predicted probability of double-stranded RNA at the particular nucleotide within the [-50..50] interval relative to the cross-link sites. Scores within 5 nucleotide bins were summed. 17
10 Protein similarity based on cDNA counts row vectors in $\mathbf{H}_{\mathrm{CLIP}}$. Note that the values for RBPs in the same groups are zero in order not to bias the clustering. 18
11 Protein similarity based on genomic region types row vectors in $\mathbf{H}_{\mathrm{RG}}$. For each region type, the interval [-50..50] relative to the cross-link sites is shown. 18
12 Protein similarity based on row vectors in $\mathbf{H}_{\mathrm{GO}}$, representing gene associations within GeneOntology (GO) annotation. 19
13 A survey of 50 most common complex motifs, estimated from basis vectors in $\mathbf{H}_{\mathrm{KMER}}$. 21
14 Example of complex motif estimation from row vectors in $\mathbf{H}_{\mathrm{KMER}}$. (left) Visualization of feature values (row vectors in $\mathbf{H}_{\mathrm{KMER}}$) of most related module to cross-link samples of PUM2 protein. (right) Complex motif derived from the row vector using procedure in Suppl. Section 7.6. The motif is similar to known motif UGUANAUA (Hafner *et al.*, 2010). The observed frequency of the derived motif in proximity to cross-link sites (blue) is greater than the expected probability at random positions within protein coding genes (green). 22
15 Levenshtein distance (D) between predicted complex RNA sequence motifs and motifs reported in (Ray *et al.*, 2013) for nine proteins included in both studies. 22
16 Sequence of the CD55 alu element bound and regulated by hnRNPC, showing hnRNPC binding site (red), regulated exon (capital letters) and motifs associated with hnRNPC (underlined). 23
17 Consesus sequences of Alu elements bound by hnRNPC in human. Reverse complements are displayed due to hnRNPC antisense binding. Identified motifs associated with hnRNPC are underlined. 24

1 Detailed information on analyzed RBP experiments

Reference and details about all experiments used in the study are listed. Experiments for the same proteins are sorted into groups A-Q. Factor models for each experiment do not consider biological or technical replicates in the same group to eliminate bias. Depending on the experimental protocol used (PARCLIP, CLIPSEQ, iCLIP, HITSCLIP) we report number of cross-linking clusters and number of individual sites (measured as the sum of cluster lengths) for each experiment used. We used information on clusters (column Clusters) if provided by the original study. In case of experiments 18-20 and 22, we used information on individual crosslink sites (column CL sites). From individual positions (clusters or individual crosslinks), we select up to 100.000 samples with highest cDNA counts.

ID	Protein	Tissue	Protocol	Ref. group	CL sites	Clusters	Reference	File name
[1]	Ago/EIF2C1-4	HEK293	PARCLIP	A	1364096	41450	Hafner *et al.* (2010)	`PARCLIP_AGO1234_hg19.bedGraph.gz`
[2]	Ago2-MNase	HEK293	PARCLIP	A	3013785	33396	Kishore *et al.* (2011)	`PARCLIP_AGO2MNASE_hg19.bedGraph.gz`
[3]	Ago2 (1)	HEK293	HITSCLIP	A	432139	7153	Boudreau *et al.* (2014)	`HITSCLIP_Ago2_binding_clusters.bedGraph.gz`
[4]	Ago2 (2)	HEK293	HITSCLIP	A	432089	7152	Boudreau *et al.* (2014)	`HITSCLIP_Ago2_binding_clusters_2.bedGraph.gz`
[5]	Ago2	HEK293	CLIPSEQ	A	2766476	27812	Kishore *et al.* (2011)	`CLIPSEQ_AGO2_hg19.bedGraph.gz`
[6]	eIF4AIII (1)	HeLa	CLIPSEQ	B	63353334	5397466	Saulière *et al.* (2012)	`CLIP-seq-eIF4AIII_1.bedGraph.gz`
[7]	eIF4AIII (2)	HeLa	CLIPSEQ	B	20925715	1693124	Saulière *et al.* (2012)	`CLIP-seq-eIF4AIII_2.bedGraph.gz`
[8]	ELAVL1	HEK293	PARCLIP	C	1202570	32129	Kishore *et al.* (2011)	`PARCLIP_ELAVL1_hg19.bedGraph.gz`
[9]	ELAVL1-MNase	HEK293	PARCLIP	C	7940664	84469	Kishore *et al.* (2011)	`PARCLIP_ELAVL1MNASE_hg19.bedGraph.g z`
[10]	ELAVL1A	HEK293	PARCLIP	C	256387	5110	Kishore *et al.* (2011)	`PARCLIP_ELAVL1A_hg19.bedGraph.gz`
[11]	ELAVL1	HEK293	CLIPSEQ	C	223121	4806	Kishore *et al.* (2011)	`CLIPSEQ_ELAVL1_hg19.bedGraph.gz`
[12]	ESWR1	HEK293	PARCLIP	D	543116	19019	Hoell *et al.* (2011)	`PARCLIP_EWSR1_hg19.bedGraph.gz`
[13]	FUS	HEK293	PARCLIP	E	1012411	39983	Hoell *et al.* (2011)	`PARCLIP_FUS_hg19.bedGraph.gz`
[14]	Mut FUS	HEK293	PARCLIP	E	380345	14953	Hoell *et al.* (2011)	`PARCLIP_FUS_mut_hg19.bedGraph.gz`
[15]	IGF2BP1-3	HEK293	PARCLIP	F	6097934	43530	Hafner *et al.* (2010)	`PARCLIP_IGF2BP123_hg19.bedGraph.gz`
[16]	hnRNPC	HeLa	iCLIP	G	4602041	438360	Zarnack *et al.* (2013)	`ICLIP_hnRNPC_Hela_iCLIP_all_clusters.bedGraph.gz`
[17]	hnRNPC	HeLa	iCLIP	G	228961	24448	König *et al.* (2010)	`ICLIP_HNRNPC_hg19.bedGraph.gz`
[18]	hnRNPL	HeLa	iCLIP	H	1125304	-	Rossbach *et al.* (2014)	`ICLIP_hnRNPL_Hela_group_3975_all-hnRNPL-Hela-hg19_sum_G_hg19-ensembl59_from_2337-2339-741_bedGraph-cDNA-hits-in-genome.bedGraph.gz`
[19]	hnRNPL	U266	iCLIP	H	123685	-	Rossbach *et al.* (2014)	`ICLIP_hnRNPL_U266_group_3986_all-hnRNPL-U266-hg19_sum_G_hg19-ensembl59_from_2485_bedGraph-cDNA-hits-in-genome.bedGraph.gz`
[20]	hnRNPL-like	U266	iCLIP	H	128958	-	Rossbach *et al.* (2014)	`ICLIP_hnRNPlike_U266_group_4000_all-hnRNPLlike-U266-hg19_sum_G_hg19-ensembl59_from_2342-2486_bedGraph-cDNA-hits-in-genome.bedGraph.gz`
[21]	MOV10	HEK293	PARCLIP	I	592451	17053	Sievers *et al.* (2012)	`PARCLIP_MOV10_Sievers_hg19.bedGraph.gz`
[22]	Nsun2	HEK293	iCLIP	J	75343	-	Hussain *et al.* (2013)	`ICLIP_NSUN2_293_group_4007_all-NSUN2-293-hg19_sum_G_hg19-ensembl59_from_3137-3202_bedGraph-cDNA-hits-in-genome.bedGraph.gz`
[23]	PUM2	HEK293	PARCLIP	K	368700	10962	Hafner *et al.* (2010)	`PARCLIP_PUM2_hg19.bedGraph.gz`
[24]	QKI	HEK293	PARCLIP	L	381140	12035	Hafner *et al.* (2010)	`PARCLIP_QKI_hg19.bedGraph.gz`
[25]	SRSF1	HEK293	CLIPSEQ	M	966912	23629	Sanford *et al.* (2009)	`CLIPSEQ_SFRS1_hg19.bedGraph.gz`
[26]	TAF15	HEK293	PARCLIP	N	222421	8677	Hoell *et al.* (2011)	`PARCLIP_TAF15_hg19.bedGraph.gz`
[27]	TDP-43	HeLa	iCLIP	O	3053718	118703	Tollervey *et al.* (2011)	`ICLIP_TDP43_hg19.bedGraph.gz`
[28]	TIA1	HeLa	iCLIP	P	393111	21884	Wang *et al.* (2010)	`ICLIP_TIA1_hg19.bedGraph.gz`
[29]	TIAL1	HeLa	iCLIP	P	1146658	51751	Wang *et al.* (2010)	`ICLIP_TIAL1_hg19.bedGraph.gz`
[30]	U2AF2	HeLa	iCLIP	Q	3668916	518794	Zarnack *et al.* (2013)	`ICLIP_U2AF65_Hela_iCLIP_ctrl_all_clusters.bedGraph.gz`
[31]	U2AF2 (KD)	HeLa	iCLIP	Q	9147292	1122142	Zarnack *et al.* (2013)	`ICLIP_U2AF65_Hela_iCLIP_ctrl+kd_all_clusters.bedGraph.gz`

Supplementary Table 1: Detailed information on individual protein-RNA experimental interaction data used. Total number of sites for each experiment. Experiments describing same protein in different technical or biological replicates are grouped.

2 Details on the iONMF algorithm

2.1 Derivation of update rules

The general matrix factorization problem can be solved with different optimization approaches; these include:

- Alternative least squares Lee *et al.* (2001) have proven convergence properties but may be computationally inefficient,
- (projected) gradient descent can be computatinally efficient while requiring manually selecting the learning rate and requires explicit control over the solutions staying in the feasible region Lin (2007),
- (quasi-)Newton methods provide a solution to the general problem of selecting the learning rate Zdunek and Cichocki (2006),
- multiplicative update rules are an instance of the gradient descent algorithm with variable learning rate, are computationally efficiet (subject to optimal ordering of matrix multiplication). Also, the non-negativity constraint is satisfied by definition if initialized matrices are non-negative.
- There is a large body of work on probabilistic matrix factorization (PMF), see Salakhutdinov and Mnih (2008), which provides improved regression results, but the non-negativity and orthogonality constraints are difficult to include in the normal distribution on which most PMF methods are based.

Due to implicit adherence to non-negativity and orthogonality constraints as well as computational efficiency, we propose a multiplicative update rule-based algorithm, which is presented in more detail below.

Non-convexity of the problem follows from observing that there exist equivalent solutions $\mathbf{X} = \mathbf{W}\mathbf{U}^T\mathbf{U}\mathbf{H}$, where $\mathbf{U}$ is any unitary matrix of appropriate size. This implies that multiple solutions exist, which yield same objective value. The problem is thus non-convex and a unique minimum does not exist.

To learn a factor model with iONMF we propose the following optimization problem with respect to $\mathbf{W}$ and $\mathbf{H}_i$ for $i = 1, ..., N$:

$$\min_{\mathbf{W},\mathbf{H}} \sum_{i=1}^{N} (\|\mathbf{X}_i - \mathbf{W}\mathbf{H}_i^T\|_F^2 + \alpha\|\mathbf{H}_i^T\mathbf{H}_i \ - \mathbf{I}\|_F^2)$$

Regularization parameter α determines the trading off between approximation error and orthogonality of vectors in $\mathbf{H}_i$. Because the optimization problem is non-convex in all $\mathbf{W}$, $\mathbf{H}_i$, local minimum can be found by fixing all but one variable and applying multiplicative update rules. The cost function in Problem 2.1 can be rewritten as:

$$\begin{aligned} J = \sum_{i=1}^{N} & tr(\mathbf{X}_i^T\mathbf{X}_i - 2\mathbf{X}_i^T\mathbf{W}\mathbf{H}_i + \mathbf{H}_i\mathbf{W}^T\mathbf{W}\mathbf{H}_i^T) \\ & +\alpha \ tr(\mathbf{H}_i^T\mathbf{H}_i\mathbf{H}_i^T\mathbf{H}_i - 2\mathbf{H}_i^T\mathbf{H}_i + \mathbf{I}^T\mathbf{I}) \end{aligned}$$

Following standard theory of constrained multivariate optimization, we construct the Lagrangian:

$$L(\mathbf{W}, \mathbf{H}_1, ..., \mathbf{H}_N, \lambda_0, \lambda_1, ..., \lambda_N) = J - tr(\lambda_0\mathbf{W}) - \sum_{i=1}^{N} tr(\lambda_i\mathbf{H}_i)$$

By fixing all $\mathbf{H}_i$, we can calculate the derivative of the Lagrangian with respect to $\mathbf{W}$:

$$\frac{\delta L}{\delta \mathbf{W}} = \sum_{i=1}^{N} -2\mathbf{X}_i\mathbf{H}_i^T + 2\mathbf{W}\mathbf{H}_i^T\mathbf{H}_i - \lambda_0$$

To satisfy the Karush-Kuhn-Tucker optimality conditions at a stationary point, the following must hold:

$$\mathbf{W} \circ \lambda_0 = 0$$

Writing $\lambda_0 = (\lambda_0^+ - \lambda_0^-)$ we have:

$$\mathbf{W}^2 \circ (\lambda_0^+ - \lambda_0^-) = 0$$

This is a fixed point equation, which can be solved by iteratively applying the following update rule:

$$\mathbf{W} = \mathbf{W} \circ \sqrt{\frac{\lambda_0^-}{\lambda_0^+}} = \mathbf{W} \circ \sqrt{\frac{\sum_{i=1}^{N}(\mathbf{X}_i\mathbf{H}_i^T)^+ + (\mathbf{W}\mathbf{H}_i^T\mathbf{H}_i)^-}{\sum_{i=1}^{N}(\mathbf{X}_i\mathbf{H}_i^T)^- + (\mathbf{W}\mathbf{H}_i^T\mathbf{H}_i)^+}}$$

Since we assume $\mathbf{X}_i$, $\mathbf{H}_i$, $\mathbf{W}$ are non-negative for all $i = 1, ..., N$, the update is equal to:

$$\mathbf{W} = \mathbf{W} \circ \sqrt{\frac{\sum_{i=1}^{N}(\mathbf{X}_i\mathbf{H}_i^T)^+}{\sum_{i=1}^{N}(\mathbf{W}\mathbf{H}_i^T\mathbf{H}_i)^+}}$$

which is the update rule given in Equation 2 (see main text).

Following a similar argument, the update rules for coefficient matrices $\mathbf{H}_i$ can be derived. Fixing $\mathbf{W}$ and all $\mathbf{H}_j$, $j \neq i$, the derivative of the Lagrangian with respect to $\mathbf{H}_i$ is equal to:

$$\frac{\delta L}{\delta \mathbf{H}_i} = -2\mathbf{X}_i^T\mathbf{W} + 2\mathbf{H}_i\mathbf{W}^T\mathbf{W} + \alpha(4\mathbf{H}_i\mathbf{H}_i^T\mathbf{H}_i - 2\mathbf{H}_i) - \lambda_i = 0$$

so that

$$\lambda_i = -\mathbf{X}_i^T\mathbf{W} + \mathbf{H}_i\mathbf{W}^T\mathbf{W} + \alpha(2\mathbf{H}_i\mathbf{H}_i^T\mathbf{H}_i - \mathbf{H}_i)$$

To satisfy the Karush-Kuhn-Tucker optimality conditions at a stationary point we must have:

$$\mathbf{H}_i \circ \lambda_i = 0$$
$$\mathbf{H}_i^2 \circ (\lambda_i^+ - \lambda_i^-) = 0$$

which leads to the following update rules:

$$\mathbf{H}_i = \mathbf{H}_i \circ \sqrt{\frac{\lambda_0^-}{\lambda_0^+}} =$$
$$= \mathbf{H}_i \circ \sqrt{\frac{(\mathbf{H}_i\mathbf{W}^T\mathbf{W})^- + 2\alpha(\mathbf{H}_i\mathbf{H}_i^T\mathbf{H}_i)^- + (\mathbf{X}_i^T\mathbf{W})^+ + \alpha(\mathbf{H}_i)^+}{(\mathbf{H}_i\mathbf{W}^T\mathbf{W})^+ + 2\alpha(\mathbf{H}_i\mathbf{H}_i^T\mathbf{H}_i)^+ + (\mathbf{X}_i^T\mathbf{W})^- + \alpha(\mathbf{H}_i)^-}}$$

$$\mathbf{H}_i = \mathbf{H}_i \circ \sqrt{\frac{(\mathbf{X}_i^T\mathbf{W})^+ + \alpha(\mathbf{H}_i)^+}{(\mathbf{H}_i\mathbf{W}^T\mathbf{W})^+ + 2\alpha(\mathbf{H}_i\mathbf{H}_i^T\mathbf{H}_i)^+}}$$

Again, this is exactly the update rule in Equation 3 (see main text). ■.

2.2 Equivalence to gradient descent

As noted by Lee *et al.* (2001), the multiplicative update rules are a special case of gradient descent.

$$\mathbf{W} = \mathbf{W} - \nu_1(\sum_i \mathbf{X}_i\mathbf{H}_i - \sum_i \mathbf{W}\mathbf{H}_i^T\mathbf{H}_i)$$
$$\mathbf{H}_i = \mathbf{H}_i - \nu_2(\mathbf{X}_i^T\mathbf{W} + \alpha\mathbf{H}_i - \mathbf{H}_i\mathbf{W}^T\mathbf{W} + 2\alpha\mathbf{H}_i\mathbf{H}_i^T\mathbf{H}_i)$$

Setting the update steps

$$\nu_1 = \frac{\mathbf{W}}{\sum_i \mathbf{W}\mathbf{H}_i^T\mathbf{H}_i}$$
$$\nu_2 = \frac{\mathbf{H}_i}{\mathbf{H}_i\mathbf{W}^T\mathbf{W} + 2\alpha\mathbf{H}_i\mathbf{H}_i^T\mathbf{H}_i}$$

yields the update rules in Equation 2 and Equation 3, respectively (see main text). Hence, the same convergence properties of gradient descent apply.

3 Comparison of iONMF, RNAContext and GraphProt

We have compared iONMF with two state-of-the-art approaches to predict putative CLIP interaction sites: GraphProt by Maticzka *et al.* (2014) and RNAContext by Kazan *et al.* (2010). We have used the same sequences and evaluation as described in Maticzka *et al.* (2014), which we downloaded from the authors' website[1].

For reasons of computational complexity, we used the published results reported by Maticzka *et al.* (2014). Running RNAContext and GraphProt with suggested parameters yielded very similar results (data not shown) as originally reported by Maticzka *et al.* (2014).

Here, we report the exact values from Maticzka *et al.* (2014), Fig. 3, for GraphProt and RNAcontext. We evaluated iONMF using the same setup. The provided *parameter fitting set* contained 500 positive and 500 negative positions were used to select the optimal subset of data sources. These same positions were originally used to fit parameters of GraphProt and RNAContext. The optimal subset of data sources and hyperparameters for iONMF were selected via 3-fold cross-validation on the *parameter fitting set*. The final evaluation was performed on the provided *validation set* using 10-fold cross-validation.

The iONMF hyperparameter α was set to 0.1 for all experiments and the factorization rank was selected from range $[10, 50]$. The half-window size for generation of matrices $\mathbf{X}_{\text{CLIP}}$, $\mathbf{X}_{\text{RG}}$, $\mathbf{X}_{\text{RNA}}$, $\mathbf{X}_{\text{KMER}}$was set to 100 nucleotides.

Two separate comparisons were performed:

1. using data sources on sequence ($\mathbf{X}_{\text{KMER}}$) and structure ($\mathbf{X}_{\text{RNA}}$), which are the sources used by GraphProt and RNAContext. See Supplementary Table 2 for AUC scores and Supplementary Table 4 for precision-recall scores.

2. using all five data sources ($\mathbf{X}_{\text{CLIP}}$, $\mathbf{X}_{\text{RG}}$, $\mathbf{X}_{\text{RNA}}$, $\mathbf{X}_{\text{KMER}}$, $\mathbf{X}_{\text{GO}}$). See Supplementary Table 3 for AUC scores and Supplementary Table 5) for precision-recall scores.

When using sequence ($\mathbf{X}_{\text{KMER}}$) and structure ($\mathbf{X}_{\text{RNA}}$), iONMF performs best on 10 out of 24 data sets. Empirically, we have found that the largest effect on the test performance of the iONMF model is due to selection of the subset of data sources. Inclusion of other data sources increases the number times iONMF is ranked first to 13 (out of 24 data sets).

The results obtained with the measures AUC and precision-recall are very similar with respect to critical distance diagrams. It is interesting to compare the differences in predictive performance within Supplementary Table 2 with the importance of sequence ($\mathbf{X}_{\text{KMER}}$) and structural information ($\mathbf{X}_{\text{RNA}}$), estimated in Supplementary Figure 5. Cases where RNAContext outperforms iONMF appear to be highly enriched in sequence-specific RBPs (ELAVL1, ESWR1, FUS, QKI, TAF15, TDP-43).

Cases where iONMF improves the most are less sequence dependent according to Supplementary Figure 5. The predictive performance seems to be compensated for in the way structural information is processed, as these cases show higher dependence on $\mathbf{X}_{\text{RNA}}$ (Ago2, iONMF AUC=0.876, next best GraphProt AUC=0.765; ALKBH5, iONMF AUC=0.805, GraphProt AUC=0.680). This underlines the flexibility of weighting the contribution of particular data sources associated with target response, which happens implicitly within the iONMF model.

Inclusion of all data sources, reported in Supplementary Table 3, improves the performance on IGF2BP1-3, MOV10 and ZC3H7B. Not surprisingly, MOV10 and IGF2BP1-3 show less dependence on sequence and more on region types, as shown in Supplementary Table 5. In this setting, iONMF yields the best predictive performance in the majority 13 out of 24 data sets (average AUC=0.907, next best GraphProt avg. AUC=0.887). According to ranks in the critical distance diagram in Supplementary Table 3, iONMF and GraphProt perform equally well on this dataset, while RNAContext is significantly lower in both cases.

[1] `http://www.bioinf.uni-freiburg.de/Software/GraphProt/`

Protein	Data sources	iONMF	GraphProt	RNAContext
ALKBH5 PAR-CLIP	$\mathbf{X}_{\mathrm{KMER}(K=4)}$, $\mathbf{X}_{\mathrm{RNA}}$	**0.805**	0.680	0.600
Ago1-4 PAR-CLIP	$\mathbf{X}_{\mathrm{RNA}}$	0.850	**0.895**	0.721
Ago2 HITS-CLIP	$\mathbf{X}_{\mathrm{KMER}(K=2)}$, $\mathbf{X}_{\mathrm{KMER}(K=4)}$, $\mathbf{X}_{\mathrm{RNA}}$	**0.876**	0.765	0.732
C17ORF85 PAR-CLIP	$\mathbf{X}_{\mathrm{RNA}}$	**0.846**	0.800	0.695
C22ORF28 PAR-CLIP	$\mathbf{X}_{\mathrm{RNA}}$	**0.845**	0.751	0.671
CAPRIN1 PAR-CLIP	$\mathbf{X}_{\mathrm{RNA}}$	**0.865**	0.855	0.650
ELAVL1 HITS-CLIP	$\mathbf{X}_{\mathrm{KMER}(K=2)}$, $\mathbf{X}_{\mathrm{RNA}}$	**0.960**	0.955	0.958
ELAVL1 PAR-CLIP (A)	$\mathbf{X}_{\mathrm{KMER}(K=2)}$, $\mathbf{X}_{\mathrm{RNA}}$	0.914	0.959	**0.962**
ELAVL1 PAR-CLIP (B)	$\mathbf{X}_{\mathrm{KMER}(K=2)}$, $\mathbf{X}_{\mathrm{RNA}}$	**0.951**	0.935	0.923
ELAVL1 PAR-CLIP (C)	$\mathbf{X}_{\mathrm{KMER}(K=4)}$	0.949	**0.991**	0.974
EWSR1 PAR-CLIP	$\mathbf{X}_{\mathrm{KMER}(K=2)}$, $\mathbf{X}_{\mathrm{KMER}(K=4)}$, $\mathbf{X}_{\mathrm{RNA}}$	0.840	**0.935**	**0.935**
FUS PAR-CLIP	$\mathbf{X}_{\mathrm{KMER}(K=4)}$	0.884	**0.968**	0.954
HNRNPC iCLIP	$\mathbf{X}_{\mathrm{KMER}(K=2)}$, $\mathbf{X}_{\mathrm{KMER}(K=4)}$	**0.960**	0.952	0.951
IGF2BP1-3 PAR-CLIP	$\mathbf{X}_{\mathrm{KMER}(K=2)}$, $\mathbf{X}_{\mathrm{RNA}}$	0.865	**0.889**	0.778
MOV10 PAR-CLIP	$\mathbf{X}_{\mathrm{KMER}(K=2)}$, $\mathbf{X}_{\mathrm{RNA}}$	0.843	**0.863**	0.750
PTB HITS-CLIP	$\mathbf{X}_{\mathrm{KMER}(K=2)}$	0.871	**0.937**	0.875
PUM2 PAR-CLIP	$\mathbf{X}_{\mathrm{KMER}(K=2)}$, $\mathbf{X}_{\mathrm{RNA}}$	0.926	**0.954**	0.906
QKI PAR-CLIP	$\mathbf{X}_{\mathrm{KMER}(K=4)}$	0.926	**0.957**	0.945
SFRS1 HITS-CLIP	$\mathbf{X}_{\mathrm{KMER}(K=2)}$, $\mathbf{X}_{\mathrm{RNA}}$	0.830	**0.898**	0.833
TAF15 PAR-CLIP	$\mathbf{X}_{\mathrm{KMER}(K=4)}$	0.913	**0.970**	0.967
TDP43 iCLIP	$\mathbf{X}_{\mathrm{KMER}(K=2)}$, $\mathbf{X}_{\mathrm{RNA}}$	0.854	**0.874**	0.828
TIA1 iCLIP	$\mathbf{X}_{\mathrm{KMER}(K=2)}$, $\mathbf{X}_{\mathrm{RNA}}$	**0.905**	0.861	0.855
TIAL1 iCLIP	$\mathbf{X}_{\mathrm{KMER}(K=2)}$	**0.860**	0.833	0.833
ZC3H7B PAR-CLIP	$\mathbf{X}_{\mathrm{KMER}(K=4)}$, $\mathbf{X}_{\mathrm{RNA}}$	0.748	**0.820**	0.636
Average		0.879 ± 0.051	0.887 ± 0.079	0.830 ± 0.119

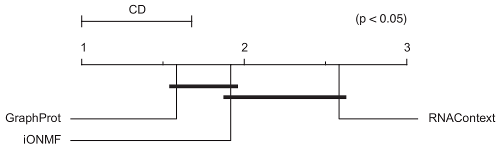

Supplementary Table 2: Comparison of AUC scores between iONMF, GraphProt and RNAContext. GraphProt and RNAContext performance values taken from Maticzka *et al.* (2014). Only sequence ($\mathbf{X}_{\mathrm{KMER}}$) and structure ($\mathbf{X}_{\mathrm{RNA}}$) data sources are used by iONMF.

Protein	Data sources	iONMF	GraphProt	RNAContext
ALKBH5 PAR-CLIP	$\mathbf{X}_{\mathrm{KMER}(K=4)}$, $\mathbf{X}_{\mathrm{RNA}}$, $\mathbf{X}_{\mathrm{CLIP}}$	**0.873**	0.680	0.600
Ago1-4 PAR-CLIP	$\mathbf{X}_{\mathrm{KMER}(K=2)}$, $\mathbf{X}_{\mathrm{RNA}}$, $\mathbf{X}_{\mathrm{CLIP}}$	0.892	**0.895**	0.721
Ago2 HITS-CLIP	$\mathbf{X}_{\mathrm{KMER}(K=2)}$, $\mathbf{X}_{\mathrm{RNA}}$, $\mathbf{X}_{\mathrm{CLIP}}$, $\mathbf{X}_{\mathrm{RG}}$	**0.921**	0.765	0.732
C17ORF85 PAR-CLIP	$\mathbf{X}_{\mathrm{KMER}(K=4)}$, $\mathbf{X}_{\mathrm{CLIP}}$	**0.862**	0.800	0.695
C22ORF28 PAR-CLIP	$\mathbf{X}_{\mathrm{RNA}}$, $\mathbf{X}_{\mathrm{CLIP}}$	**0.912**	0.751	0.671
CAPRIN1 PAR-CLIP	$\mathbf{X}_{\mathrm{RNA}}$, $\mathbf{X}_{\mathrm{CLIP}}$, $\mathbf{X}_{\mathrm{RG}}$	**0.928**	0.855	0.650
ELAVL1 HITS-CLIP	$\mathbf{X}_{\mathrm{KMER}(K=2)}$, $\mathbf{X}_{\mathrm{CLIP}}$	**0.963**	0.955	0.958
ELAVL1 PAR-CLIP (A)	$\mathbf{X}_{\mathrm{KMER}(K=2)}$, $\mathbf{X}_{\mathrm{KMER}(K=4)}$, $\mathbf{X}_{\mathrm{CLIP}}$	0.933	0.959	**0.962**
ELAVL1 PAR-CLIP (B)	$\mathbf{X}_{\mathrm{KMER}(K=2)}$, $\mathbf{X}_{\mathrm{RNA}}$, $\mathbf{X}_{\mathrm{CLIP}}$	**0.972**	0.935	0.923
ELAVL1 PAR-CLIP (C)	$\mathbf{X}_{\mathrm{KMER}(K=2)}$, $\mathbf{X}_{\mathrm{KMER}(K=4)}$, $\mathbf{X}_{\mathrm{CLIP}}$	0.958	**0.991**	0.974
EWSR1 PAR-CLIP	$\mathbf{X}_{\mathrm{KMER}(K=2)}$, $\mathbf{X}_{\mathrm{CLIP}}$	0.871	**0.935**	**0.935**
FUS PAR-CLIP	$\mathbf{X}_{\mathrm{KMER}(K=2)}$, $\mathbf{X}_{\mathrm{KMER}(K=4)}$, $\mathbf{X}_{\mathrm{CLIP}}$	0.827	**0.968**	0.954
HNRNPC iCLIP	$\mathbf{X}_{\mathrm{KMER}(K=2)}$, $\mathbf{X}_{\mathrm{CLIP}}$	**0.959**	0.952	0.951
IGF2BP1-3 PAR-CLIP	$\mathbf{X}_{\mathrm{KMER}(K=2)}$, $\mathbf{X}_{\mathrm{KMER}(K=4)}$, $\mathbf{X}_{\mathrm{RNA}}$, $\mathbf{X}_{\mathrm{CLIP}}$, $\mathbf{X}_{\mathrm{RG}}$	**0.928**	0.889	0.778
MOV10 PAR-CLIP	$\mathbf{X}_{\mathrm{RNA}}$, $\mathbf{X}_{\mathrm{CLIP}}$, $\mathbf{X}_{\mathrm{RG}}$	**0.939**	0.863	0.750
PTB HITS-CLIP	$\mathbf{X}_{\mathrm{KMER}(K=2)}$	0.860	**0.937**	0.875
PUM2 PAR-CLIP	$\mathbf{X}_{\mathrm{KMER}(K=2)}$, $\mathbf{X}_{\mathrm{KMER}(K=4)}$, $\mathbf{X}_{\mathrm{RNA}}$, $\mathbf{X}_{\mathrm{CLIP}}$, $\mathbf{X}_{\mathrm{RG}}$	0.942	**0.954**	0.906
QKI PAR-CLIP	$\mathbf{X}_{\mathrm{KMER}(K=4)}$, $\mathbf{X}_{\mathrm{RNA}}$	0.880	**0.957**	0.945
SFRS1 HITS-CLIP	$\mathbf{X}_{\mathrm{KMER}(K=2)}$, $\mathbf{X}_{\mathrm{KMER}(K=4)}$, $\mathbf{X}_{\mathrm{RNA}}$, $\mathbf{X}_{\mathrm{CLIP}}$, $\mathbf{X}_{\mathrm{RG}}$	0.857	**0.898**	0.833
TAF15 PAR-CLIP	$\mathbf{X}_{\mathrm{KMER}(K=2)}$, $\mathbf{X}_{\mathrm{KMER}(K=4)}$, $\mathbf{X}_{\mathrm{RNA}}$, $\mathbf{X}_{\mathrm{CLIP}}$	0.927	**0.970**	0.967
TDP43 iCLIP	$\mathbf{X}_{\mathrm{KMER}(K=2)}$, $\mathbf{X}_{\mathrm{RNA}}$	0.855	**0.874**	0.828
TIA1 iCLIP	$\mathbf{X}_{\mathrm{KMER}(K=2)}$, $\mathbf{X}_{\mathrm{RNA}}$, $\mathbf{X}_{\mathrm{CLIP}}$	**0.937**	0.861	0.855
TIAL1 iCLIP	$\mathbf{X}_{\mathrm{KMER}(K=2)}$, $\mathbf{X}_{\mathrm{KMER}(K=4)}$, $\mathbf{X}_{\mathrm{CLIP}}$, $\mathbf{X}_{\mathrm{RG}}$	**0.915**	0.833	0.833
ZC3H7B PAR-CLIP	$\mathbf{X}_{\mathrm{KMER}(K=2)}$, $\mathbf{X}_{\mathrm{RNA}}$, $\mathbf{X}_{\mathrm{CLIP}}$	**0.849**	0.820	0.636
Average		0.907 ± 0.041	0.887 ± 0.079	0.830 ± 0.119

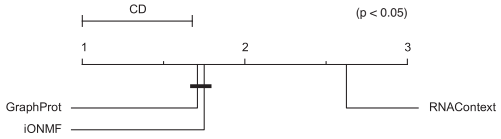

Supplementary Table 3: Comparison of AUC scores between iONMF, GraphProt and RNAContext. GraphProt and RNAContext performance values taken from Maticzka *et al.* (2014). All available data sources are used by iONMF.

Protein	Data sources	iONMF	GraphProt	RNAContext
ALKBH5 PAR-CLIP	$\mathbf{X}_{\mathrm{RNA}}$	**0.785**	0.669	0.585
Ago1-4 PAR-CLIP	$\mathbf{X}_{\mathrm{KMER(K=2)}}$, $\mathbf{X}_{\mathrm{KMER(K=4)}}$, $\mathbf{X}_{\mathrm{RNA}}$	0.819	**0.906**	0.730
Ago2 HITS-CLIP	$\mathbf{X}_{\mathrm{KMER(K=2)}}$, $\mathbf{X}_{\mathrm{KMER(K=4)}}$, $\mathbf{X}_{\mathrm{RNA}}$	**0.856**	0.756	0.715
C17ORF85 PAR-CLIP	$\mathbf{X}_{\mathrm{KMER(K=2)}}$, $\mathbf{X}_{\mathrm{KMER(K=4)}}$, $\mathbf{X}_{\mathrm{RNA}}$	**0.799**	0.775	0.670
C22ORF28 PAR-CLIP	$\mathbf{X}_{\mathrm{KMER(K=2)}}$, $\mathbf{X}_{\mathrm{RNA}}$	0.695	**0.746**	0.676
CAPRIN1 PAR-CLIP	$\mathbf{X}_{\mathrm{KMER(K=4)}}$, $\mathbf{X}_{\mathrm{RNA}}$	0.747	**0.851**	0.635
ELAVL1 HITS-CLIP	$\mathbf{X}_{\mathrm{KMER(K=2)}}$, $\mathbf{X}_{\mathrm{RNA}}$	**0.949**	0.940	0.943
ELAVL1 PAR-CLIP (A)	$\mathbf{X}_{\mathrm{KMER(K=2)}}$, $\mathbf{X}_{\mathrm{KMER(K=4)}}$, $\mathbf{X}_{\mathrm{RNA}}$	0.913	0.951	**0.953**
ELAVL1 PAR-CLIP (B)	$\mathbf{X}_{\mathrm{KMER(K=2)}}$, $\mathbf{X}_{\mathrm{RNA}}$	**0.951**	0.935	0.918
ELAVL1 PAR-CLIP (C)	$\mathbf{X}_{\mathrm{KMER(K=2)}}$, $\mathbf{X}_{\mathrm{RNA}}$	0.928	**0.992**	0.972
EWSR1 PAR-CLIP	$\mathbf{X}_{\mathrm{KMER(K=2)}}$, $\mathbf{X}_{\mathrm{RNA}}$	0.818	**0.942**	0.936
FUS PAR-CLIP	$\mathbf{X}_{\mathrm{KMER(K=2)}}$, $\mathbf{X}_{\mathrm{KMER(K=4)}}$	0.812	**0.970**	0.953
HNRNPC iCLIP	$\mathbf{X}_{\mathrm{KMER(K=4)}}$	**0.963**	0.947	0.947
IGF2BP1-3 PAR-CLIP	$\mathbf{X}_{\mathrm{KMER(K=2)}}$, $\mathbf{X}_{\mathrm{KMER(K=4)}}$, $\mathbf{X}_{\mathrm{RNA}}$	0.883	**0.901**	0.792
MOV10 PAR-CLIP	$\mathbf{X}_{\mathrm{KMER(K=2)}}$, $\mathbf{X}_{\mathrm{RNA}}$	0.821	**0.853**	0.715
PTB HITS-CLIP	$\mathbf{X}_{\mathrm{KMER(K=2)}}$, $\mathbf{X}_{\mathrm{KMER(K=4)}}$	0.867	**0.925**	0.863
PUM2 PAR-CLIP	$\mathbf{X}_{\mathrm{KMER(K=2)}}$, $\mathbf{X}_{\mathrm{RNA}}$	0.927	**0.958**	0.917
QKI PAR-CLIP	$\mathbf{X}_{\mathrm{KMER(K=4)}}$, $\mathbf{X}_{\mathrm{RNA}}$	0.936	**0.971**	0.964
SFRS1 HITS-CLIP	$\mathbf{X}_{\mathrm{KMER(K=4)}}$	0.828	**0.898**	0.842
TAF15 PAR-CLIP	$\mathbf{X}_{\mathrm{KMER(K=4)}}$	0.885	**0.973**	0.969
TDP43 iCLIP	$\mathbf{X}_{\mathrm{KMER(K=2)}}$	0.879	**0.895**	0.864
TIA1 iCLIP	$\mathbf{X}_{\mathrm{KMER(K=2)}}$, $\mathbf{X}_{\mathrm{KMER(K=4)}}$, $\mathbf{X}_{\mathrm{RNA}}$	**0.900**	0.842	0.837
TIAL1 iCLIP	$\mathbf{X}_{\mathrm{KMER(K=2)}}$	**0.830**	0.819	0.819
ZC3H7B PAR-CLIP	$\mathbf{X}_{\mathrm{RNA}}$	0.754	**0.813**	0.613
Average		0.856 ± 0.070	0.884 ± 0.083	0.826 ± 0.125

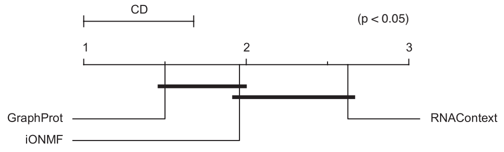

Supplementary Table 4: Comparison of precision-recall scores between iONMF, GraphProt and RNAContext. GraphProt and RNAContext performance values taken from Maticzka *et al.* (2014). Only sequence ($\mathbf{X}_{\mathrm{KMER}}$) and structure ($\mathbf{X}_{\mathrm{RNA}}$) data sources are used by iONMF.

Protein	Data sources	iONMF	GraphProt	RNAContext
ALKBH5 PAR-CLIP	$\mathbf{X}_{\mathrm{KMER(K=4)}}$, $\mathbf{X}_{\mathrm{RNA}}$, $\mathbf{X}_{\mathrm{CLIP}}$, $\mathbf{X}_{\mathrm{RG}}$	**0.868**	0.669	0.585
Ago1-4 PAR-CLIP	$\mathbf{X}_{\mathrm{KMER(K=2)}}$, $\mathbf{X}_{\mathrm{RNA}}$, $\mathbf{X}_{\mathrm{CLIP}}$	0.899	**0.906**	0.730
Ago2 HITS-CLIP	$\mathbf{X}_{\mathrm{KMER(K=2)}}$, $\mathbf{X}_{\mathrm{KMER(K=4)}}$, $\mathbf{X}_{\mathrm{RNA}}$, $\mathbf{X}_{\mathrm{CLIP}}$, $\mathbf{X}_{\mathrm{RG}}$	**0.929**	0.756	0.715
C17ORF85 PAR-CLIP	$\mathbf{X}_{\mathrm{RNA}}$, $\mathbf{X}_{\mathrm{CLIP}}$	**0.860**	0.775	0.670
C22ORF28 PAR-CLIP	$\mathbf{X}_{\mathrm{KMER(K=2)}}$, $\mathbf{X}_{\mathrm{KMER(K=4)}}$, $\mathbf{X}_{\mathrm{RNA}}$, $\mathbf{X}_{\mathrm{CLIP}}$	**0.910**	0.746	0.676
CAPRIN1 PAR-CLIP	$\mathbf{X}_{\mathrm{RNA}}$, $\mathbf{X}_{\mathrm{CLIP}}$, $\mathbf{X}_{\mathrm{RG}}$	**0.912**	0.851	0.635
ELAVL1 HITS-CLIP	$\mathbf{X}_{\mathrm{KMER(K=2)}}$, $\mathbf{X}_{\mathrm{CLIP}}$	**0.963**	0.940	0.943
ELAVL1 PAR-CLIP (A)	$\mathbf{X}_{\mathrm{KMER(K=2)}}$, $\mathbf{X}_{\mathrm{CLIP}}$	0.930	0.951	**0.953**
ELAVL1 PAR-CLIP (B)	$\mathbf{X}_{\mathrm{KMER(K=2)}}$, $\mathbf{X}_{\mathrm{KMER(K=4)}}$, $\mathbf{X}_{\mathrm{RNA}}$, $\mathbf{X}_{\mathrm{CLIP}}$	**0.970**	0.935	0.918
ELAVL1 PAR-CLIP (C)	$\mathbf{X}_{\mathrm{KMER(K=2)}}$, $\mathbf{X}_{\mathrm{CLIP}}$	0.948	**0.992**	0.972
EWSR1 PAR-CLIP	$\mathbf{X}_{\mathrm{KMER(K=2)}}$, $\mathbf{X}_{\mathrm{CLIP}}$	0.898	**0.942**	0.936
FUS PAR-CLIP	$\mathbf{X}_{\mathrm{KMER(K=2)}}$, $\mathbf{X}_{\mathrm{KMER(K=4)}}$, $\mathbf{X}_{\mathrm{CLIP}}$	0.821	**0.970**	0.953
HNRNPC iCLIP	$\mathbf{X}_{\mathrm{KMER(K=2)}}$, $\mathbf{X}_{\mathrm{RNA}}$, $\mathbf{X}_{\mathrm{CLIP}}$	**0.962**	0.947	0.947
IGF2BP1-3 PAR-CLIP	$\mathbf{X}_{\mathrm{KMER(K=2)}}$, $\mathbf{X}_{\mathrm{KMER(K=4)}}$, $\mathbf{X}_{\mathrm{RNA}}$, $\mathbf{X}_{\mathrm{CLIP}}$, $\mathbf{X}_{\mathrm{RG}}$	**0.930**	0.901	0.792
MOV10 PAR-CLIP	$\mathbf{X}_{\mathrm{KMER(K=4)}}$, $\mathbf{X}_{\mathrm{RNA}}$, $\mathbf{X}_{\mathrm{CLIP}}$, $\mathbf{X}_{\mathrm{RG}}$	**0.935**	0.853	0.715
PTB HITS-CLIP	$\mathbf{X}_{\mathrm{KMER(K=2)}}$, $\mathbf{X}_{\mathrm{KMER(K=4)}}$	0.866	**0.925**	0.863
PUM2 PAR-CLIP	$\mathbf{X}_{\mathrm{KMER(K=2)}}$, $\mathbf{X}_{\mathrm{CLIP}}$, $\mathbf{X}_{\mathrm{RG}}$	0.937	**0.958**	0.917
QKI PAR-CLIP	$\mathbf{X}_{\mathrm{KMER(K=4)}}$	0.949	**0.971**	0.964
SFRS1 HITS-CLIP	$\mathbf{X}_{\mathrm{KMER(K=2)}}$, $\mathbf{X}_{\mathrm{CLIP}}$, $\mathbf{X}_{\mathrm{RG}}$	0.848	**0.898**	0.842
TAF15 PAR-CLIP	$\mathbf{X}_{\mathrm{KMER(K=2)}}$, $\mathbf{X}_{\mathrm{CLIP}}$	0.945	**0.973**	0.969
TDP43 iCLIP	$\mathbf{X}_{\mathrm{KMER(K=2)}}$	0.880	**0.895**	0.864
TIA1 iCLIP	$\mathbf{X}_{\mathrm{KMER(K=2)}}$, $\mathbf{X}_{\mathrm{KMER(K=4)}}$, $\mathbf{X}_{\mathrm{CLIP}}$	**0.932**	0.842	0.837
TIAL1 iCLIP	$\mathbf{X}_{\mathrm{KMER(K=2)}}$, $\mathbf{X}_{\mathrm{RNA}}$, $\mathbf{X}_{\mathrm{CLIP}}$	**0.924**	0.819	0.819
ZC3H7B PAR-CLIP	$\mathbf{X}_{\mathrm{KMER(K=2)}}$, $\mathbf{X}_{\mathrm{CLIP}}$	**0.839**	0.813	0.613
Average		0.911 ± 0.041	0.884 ± 0.083	0.826 ± 0.125

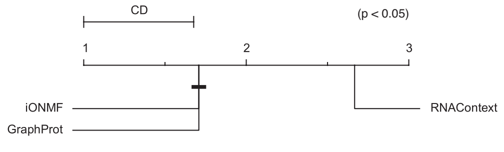

Supplementary Table 5: Comparison of precision-recall scores between iONMF, GraphProt and RNAContext. GraphProt and RNAContext performance values taken from Maticzka *et al.* (2014). All available data sources are used by iONMF.

4 Empirical evaluation of orthogonality-regularized factor models

4.1 Effect of orthogonality on predictive performance and model sparseness

The number of training examples can affect the performance of factor models. In Suppl. Fig. 1, we show how the performance of iONMF and NMF changes with increasing training set size. For all experiments, the hyperparameter α was chosen from the interval $[10^{-3}...10^{3}]$ via three-fold cross-validation. As the number of training examples increases, the gap between predictive performance of iONMF vs. NMF increases as well.

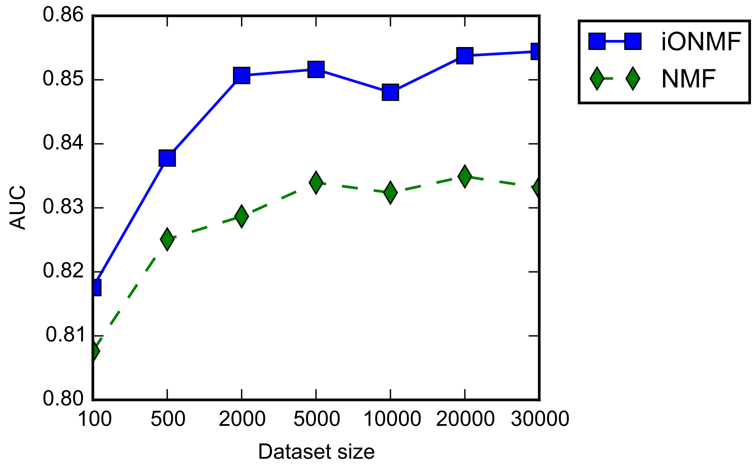

Supplementary Fig. 1: Average performance of iONMF and NMF over 31 RBP experiments, depending on the size of the training set. The test set contains 1000 positions with 20% positives.

Effect of parameter α on average sparseness and average angle between all pairs of basis vectors $\mathbf{H}_i$ is shown on Suppl. Figure 2. Note, because all vectors are non-negative, the maximum angle is 90°, representing and orthogonal model. As α increases (from left to right) both measures increase by a large amount, while retaining a similar level of predictive performance (AUC).

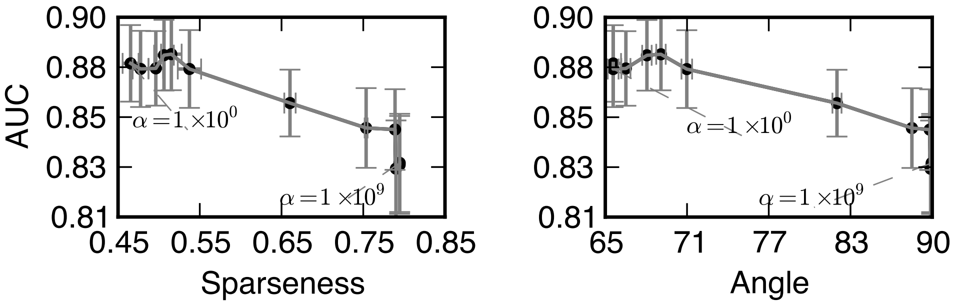

Supplementary Fig. 2: Effect of parameter α on performance (y axis), sparseness (left), and angle of basis vectors $\mathbf{H}_i$ (right) of model obtained with iONMF.

4.2 Correlation among feature vectors (predictors)

To illustrate the advantage of orthogonal decomposition, we examine the differences in feature vectors discovered by each matrix factorization method in more detail. Orthogonality is related to the phenomenon of *multicollinearity*, where multiple feature vectors in a model are highly correlated Chatterjee and Hadi (2015). This may lead to suboptimal prediction performance and can have an effect on the magnitude of particular regression coefficients. Our framework can be seen as learning multiple regression models, where each row $\mathbf{x}_j \in \mathbf{X}$ is predicted by a (non-negative) linear combination of feature vectors in $\mathbf{H}$ given by coefficients in row $\mathbf{w}_j$.

We examine the models used for obtaining results in Section and Table 1 (see main text) in more detail. For each of the methods iONMF, NMF, and NMF-QNO[2], we select the model resulting from parameters with best performance on the test set. Recall that each model produces the coefficient matrices $\mathbf{H}_i$, each with rank $r = 10$.

For each RBP experiment and model, we examine the feature vectors in each $\mathbf{H}_i$. Let ρ_j be the maximal Pearson correlation of each feature vector $h_j \in \mathbf{H}$ with all other feature fectors in $\mathbf{H}$. Supplementary Fig. 3 shows the average maximal correlation $\bar{\rho}$ computed over 10 feature vectors in a particular model.

We can observe iONMF found models that have least $\bar{\rho}$ in 30 out of 31 experiments. Also, the feature vectors are on average the least correlated in models found by iONMF when averaged over all 31 experiments (shown by horizontal lines in Supplementary Fig. 3). This results support the claim that multicollinearity is alleviated and explain the improved predictive performance measured by AUC (Table 1, see main text).

The feature vectors obtained by SNMF report an $\bar{\rho} = 0.01$ and were not included into the comparison due to inferior predictive performance resulting from over-sparse vectors.

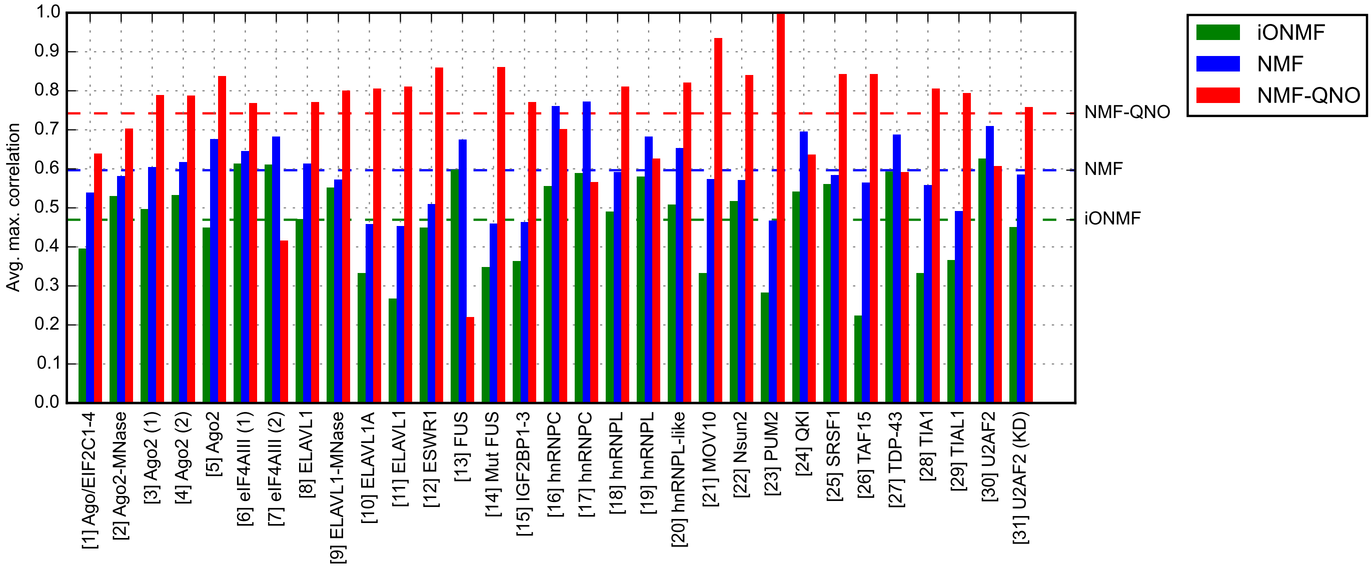

Supplementary Fig. 3: Comparison of average maximal pairwise correlation between low-rank components (rows in $\mathbf{H}$).

4.3 Comparison of discovered modules

Comparison of most relevant component found by NMF (Suppl. Fig4a), and two most relevant component found by iONMF (Suppl. Fig4b, c). Shown are the corresponding column vectors in $\mathbf{H}_{\text{KMER}}$ with five 4-mers with highest coefficient values.

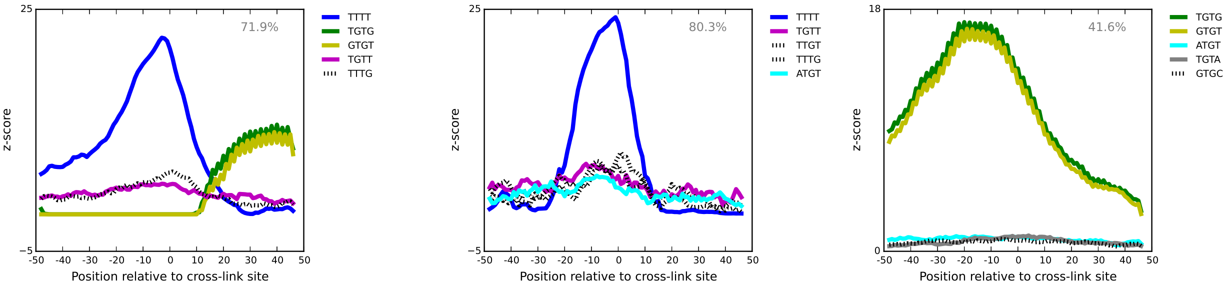

Supplementary Fig. 4: Comparison of vectors in $\mathbf{H}_{\text{KMER}}$ related to cross-links of [27] TDP-43. **left:** Non-regularized NMF. **Middle, right:** Top two relavant components found by iONMF.

[2] http://www.bsp.brain.riken.go.jp/ICALAB/

5 Details on models learned from subsets of data sources

5.1 Model parameters

In this section, we present details on parameter settings (factorization rank) for factor models learned on different subsets of data sources. The model learned on the complete set of data sources (CRTKG), with $m = 50000$ training samples, expected rank $r_c = 10$ and number of target columns n_Y requires $N_c = (m + n_Y + n) \times r_c = (m + 1 + 3030 + 101 + 505 + 25865 + 39560) \times r_c = 1190570$ free parameters. This is the total number of entries in matrices $\mathbf{W}$, $\mathbf{H}_Y$ and all $\mathbf{H}_i$ of a particular subset, where total number of features is $n = \sum_i n_i$. Note that the $\mathbf{X}_{\mathrm{CLIP}}$matrix has up to 3030 columns, the exact number is depending on the replicate group corresponding to the selected protein. To ensure fair comparison among different combinations of data sources, we set the rank to $r = \lceil N_c/(m + n_Y + n) \rceil$, for each combination of data sources separately. This ensures an approximately equal number of free parameters of the factor model (column N in Suppl. Table 6) that need to be fit.

Subset name	Data subset S	$\sum_i n_i$	No. params. N	Rank r	Avg. AUC
C	$\mathbf{X}_{\mathrm{CLIP}}$	3030	1219713	23	0.733 ± 0.018
G	$\mathbf{X}_{\mathrm{GO}}$	101	1202448	24	0.493 ± 0.009
K	$\mathbf{X}_{\mathrm{KMER}}$	505	1212144	24	0.690 ± 0.017
R	$\mathbf{X}_{\mathrm{RNA}}$	25856	1213712	16	0.744 ± 0.024
T	$\mathbf{X}_{\mathrm{RG}}$	39560	1253854	14	0.704 ± 0.018
CG	$\mathbf{X}_{\mathrm{CLIP}}$,$\mathbf{X}_{\mathrm{GO}}$	3131	1222059	23	0.701 ± 0.021
CK	$\mathbf{X}_{\mathrm{CLIP}}$,$\mathbf{X}_{\mathrm{KMER}}$	3535	1231351	23	0.820 ± 0.020
CR	$\mathbf{X}_{\mathrm{CLIP}}$,$\mathbf{X}_{\mathrm{RNA}}$	28886	1262208	16	0.788 ± 0.023
CT	$\mathbf{X}_{\mathrm{CLIP}}$,$\mathbf{X}_{\mathrm{RG}}$	42590	1203696	13	0.796 ± 0.021
GK	$\mathbf{X}_{\mathrm{GO}}$,$\mathbf{X}_{\mathrm{KMER}}$	606	1214592	24	0.776 ± 0.020
GR	$\mathbf{X}_{\mathrm{GO}}$,$\mathbf{X}_{\mathrm{RNA}}$	25957	1215344	16	0.699 ± 0.026
GT	$\mathbf{X}_{\mathrm{GO}}$,$\mathbf{X}_{\mathrm{RG}}$	39661	1255282	14	0.816 ± 0.015
KR	$\mathbf{X}_{\mathrm{KMER}}$,$\mathbf{X}_{\mathrm{RNA}}$	26361	1221808	16	0.763 ± 0.019
KT	$\mathbf{X}_{\mathrm{KMER}}$,$\mathbf{X}_{\mathrm{RG}}$	40065	1260938	14	0.860 ± 0.018
RT	$\mathbf{X}_{\mathrm{RNA}}$,$\mathbf{X}_{\mathrm{RG}}$	65416	1269598	11	0.735 ± 0.022
CGK	$\mathbf{X}_{\mathrm{CLIP}}$,$\mathbf{X}_{\mathrm{GO}}$,$\mathbf{X}_{\mathrm{KMER}}$	3636	1233697	23	0.842 ± 0.016
CGR	$\mathbf{X}_{\mathrm{CLIP}}$,$\mathbf{X}_{\mathrm{GO}}$,$\mathbf{X}_{\mathrm{RNA}}$	28987	1263840	16	0.774 ± 0.026
CGT	$\mathbf{X}_{\mathrm{CLIP}}$,$\mathbf{X}_{\mathrm{GO}}$,$\mathbf{X}_{\mathrm{RG}}$	42691	1205022	13	0.858 ± 0.018
CKR	$\mathbf{X}_{\mathrm{CLIP}}$,$\mathbf{X}_{\mathrm{KMER}}$,$\mathbf{X}_{\mathrm{RNA}}$	29391	1190910	15	0.911 ± 0.000
CKT	$\mathbf{X}_{\mathrm{CLIP}}$,$\mathbf{X}_{\mathrm{KMER}}$,$\mathbf{X}_{\mathrm{RG}}$	43095	1210274	13	0.910 ± 0.000
CRT	$\mathbf{X}_{\mathrm{CLIP}}$,$\mathbf{X}_{\mathrm{RNA}}$,$\mathbf{X}_{\mathrm{RG}}$	68446	1302939	11	0.807 ± 0.021
GKR	$\mathbf{X}_{\mathrm{GO}}$,$\mathbf{X}_{\mathrm{KMER}}$,$\mathbf{X}_{\mathrm{RNA}}$	26462	1223440	16	0.830 ± 0.017
GKT	$\mathbf{X}_{\mathrm{GO}}$,$\mathbf{X}_{\mathrm{KMER}}$,$\mathbf{X}_{\mathrm{RG}}$	40166	1262366	14	0.884 ± 0.008
GRT	$\mathbf{X}_{\mathrm{GO}}$,$\mathbf{X}_{\mathrm{RNA}}$,$\mathbf{X}_{\mathrm{RG}}$	65517	1270720	11	0.834 ± 0.015
KRT	$\mathbf{X}_{\mathrm{KMER}}$,$\mathbf{X}_{\mathrm{RNA}}$,$\mathbf{X}_{\mathrm{RG}}$	65921	1275164	11	0.873 ± 0.016
CGKR	$\mathbf{X}_{\mathrm{CLIP}}$,$\mathbf{X}_{\mathrm{GO}}$,$\mathbf{X}_{\mathrm{KMER}}$,$\mathbf{X}_{\mathrm{RNA}}$	29492	1192440	15	0.903 ± 0.008
CGKT	$\mathbf{X}_{\mathrm{CLIP}}$,$\mathbf{X}_{\mathrm{GO}}$,$\mathbf{X}_{\mathrm{KMER}}$,$\mathbf{X}_{\mathrm{RG}}$	43196	1211600	13	0.880 ± 0.011
CGRT	$\mathbf{X}_{\mathrm{CLIP}}$,$\mathbf{X}_{\mathrm{GO}}$,$\mathbf{X}_{\mathrm{RNA}}$,$\mathbf{X}_{\mathrm{RG}}$	68547	1304061	11	0.863 ± 0.016
CKRT	$\mathbf{X}_{\mathrm{CLIP}}$,$\mathbf{X}_{\mathrm{KMER}}$,$\mathbf{X}_{\mathrm{RNA}}$,$\mathbf{X}_{\mathrm{RG}}$	68951	1308505	11	0.921 ± 0.007
GKRT	$\mathbf{X}_{\mathrm{GO}}$,$\mathbf{X}_{\mathrm{KMER}}$,$\mathbf{X}_{\mathrm{RNA}}$,$\mathbf{X}_{\mathrm{RG}}$	66022	1276286	11	0.878 ± 0.012
CGKRT	$\mathbf{X}_{\mathrm{CLIP}}$,$\mathbf{X}_{\mathrm{GO}}$,$\mathbf{X}_{\mathrm{KMER}}$,$\mathbf{X}_{\mathrm{RNA}}$,$\mathbf{X}_{\mathrm{RG}}$	69052	1190570	10	0.887 ± 0.011

Supplementary Table 6: Details on parameter settings for factor models on differet subsets of data sources.

5.2 Prediction accuracy for data source subsets on individual RBPs

Each combination of RBP experiments and subset of data sources yields a factor model. In Suppl. Tables 7,8 and 9 we compare all such models on area under ROC curve (AUC), obtained via prediction of the independent hold-out test set (see main text). For each protein, we highlight the best-scoring subset of data sources.

Subset	[1] Ago/EIF2C1-4	[2] Ago2-MNase	[3] Ago2 (1)	[4] Ago2 (2)	[5] Ago2	[6] eIF4AIII (1)	[7] eIF4AIII (2)	[8] ELAVL1	[9] ELAVL1-MNase	[10] ELAVL1A
$\mathbf{X}_{\mathrm{CLIP}}$	0.805	0.716	0.689	0.698	0.711	0.729	0.716	0.783	0.688	0.927
$\mathbf{X}_{\mathrm{GO}}$	0.497	0.505	0.515	0.527	0.505	0.439	0.513	0.519	0.512	0.540
$\mathbf{X}_{\mathrm{KMER}}$	0.601	0.518	0.641	0.660	0.523	0.776	0.762	0.682	0.530	0.660
$\mathbf{X}_{\mathrm{RNA}}$	0.885	0.705	0.874	0.870	0.691	0.926	0.939	0.749	0.736	0.907
$\mathbf{X}_{\mathrm{RG}}$	0.732	0.632	0.771	0.584	0.634	0.797	0.752	0.651	0.638	0.964
$\mathbf{X}_{\mathrm{CLIP}}\mathbf{X}_{\mathrm{GO}}$	0.751	0.713	0.662	0.697	0.674	0.667	0.675	0.774	0.672	0.932
$\mathbf{X}_{\mathrm{CLIP}}\mathbf{X}_{\mathrm{KMER}}$	0.698	0.601	0.761	0.742	0.593	0.863	0.863	0.924	0.594	0.960
$\mathbf{X}_{\mathrm{CLIP}}\mathbf{X}_{\mathrm{RNA}}$	0.909	0.745	0.889	0.887	0.720	0.926	0.938	0.810	0.749	0.943
$\mathbf{X}_{\mathrm{CLIP}}\mathbf{X}_{\mathrm{RG}}$	0.907	0.691	0.881	0.879	0.661	0.897	0.920	0.847	0.704	0.980
$\mathbf{X}_{\mathrm{GO}}\mathbf{X}_{\mathrm{KMER}}$	0.625	0.538	0.718	0.725	0.582	0.853	0.889	0.789	0.585	0.848
$\mathbf{X}_{\mathrm{KMER}}\mathbf{X}_{\mathrm{RNA}}$	0.685	0.543	0.805	0.750	0.551	0.909	0.947	0.725	0.558	0.877
$\mathbf{X}_{\mathrm{KMER}}\mathbf{X}_{\mathrm{RG}}$	0.886	0.617	0.920	0.925	0.696	0.931	0.935	0.943	0.621	0.967
$\mathbf{X}_{\mathrm{GO}}\mathbf{X}_{\mathrm{RNA}}$	0.808	0.695	0.829	0.854	0.659	0.877	0.922	0.713	0.683	0.908
$\mathbf{X}_{\mathrm{GO}}\mathbf{X}_{\mathrm{RG}}$	0.884	0.778	0.883	0.895	0.785	0.901	0.898	0.807	0.811	0.973
$\mathbf{X}_{\mathrm{RNA}}\mathbf{X}_{\mathrm{RG}}$	0.856	0.645	0.815	0.717	0.656	0.883	0.949	0.600	0.620	0.968
$\mathbf{X}_{\mathrm{CLIP}}\mathbf{X}_{\mathrm{GO}}\mathbf{X}_{\mathrm{KMER}}$	0.770	0.710	0.744	0.759	0.718	0.791	0.777	0.934	0.678	0.968
$\mathbf{X}_{\mathrm{CLIP}}\mathbf{X}_{\mathrm{KMER}}\mathbf{X}_{\mathrm{RNA}}$	0.831	0.592	0.816	0.802	0.600	0.946	0.955	0.943	0.590	0.967
$\mathbf{X}_{\mathrm{CLIP}}\mathbf{X}_{\mathrm{KMER}}\mathbf{X}_{\mathrm{RG}}$	0.913	0.714	**0.928**	0.925	0.752	0.939	0.942	**0.954**	0.723	0.974
$\mathbf{X}_{\mathrm{CLIP}}\mathbf{X}_{\mathrm{GO}}\mathbf{X}_{\mathrm{RNA}}$	0.915	0.748	0.843	0.880	0.700	0.938	0.944	0.805	0.740	0.963
$\mathbf{X}_{\mathrm{CLIP}}\mathbf{X}_{\mathrm{GO}}\mathbf{X}_{\mathrm{RG}}$	0.909	0.824	0.897	0.906	0.816	0.932	0.926	0.861	0.816	**0.984**
$\mathbf{X}_{\mathrm{CLIP}}\mathbf{X}_{\mathrm{RNA}}\mathbf{X}_{\mathrm{RG}}$	0.860	0.690	0.859	0.908	0.667	0.952	0.962	0.811	0.670	0.980
$\mathbf{X}_{\mathrm{GO}}\mathbf{X}_{\mathrm{KMER}}\mathbf{X}_{\mathrm{RNA}}$	0.719	0.628	0.829	0.853	0.596	0.924	0.939	0.888	0.691	0.905
$\mathbf{X}_{\mathrm{GO}}\mathbf{X}_{\mathrm{KMER}}\mathbf{X}_{\mathrm{RG}}$	0.885	0.808	0.903	0.912	0.775	0.900	0.892	0.921	0.819	0.969
$\mathbf{X}_{\mathrm{KMER}}\mathbf{X}_{\mathrm{RNA}}\mathbf{X}_{\mathrm{RG}}$	0.890	0.659	0.918	0.929	0.713	0.951	0.959	0.932	0.649	0.970
$\mathbf{X}_{\mathrm{GO}}\mathbf{X}_{\mathrm{RNA}}\mathbf{X}_{\mathrm{RG}}$	0.901	0.810	0.906	0.911	0.779	0.936	0.953	0.840	0.817	0.972
$\mathbf{X}_{\mathrm{CLIP}}\mathbf{X}_{\mathrm{GO}}\mathbf{X}_{\mathrm{KMER}}\mathbf{X}_{\mathrm{RNA}}$	0.919	0.771	0.910	0.919	0.752	0.942	0.957	0.951	0.753	0.967
$\mathbf{X}_{\mathrm{CLIP}}\mathbf{X}_{\mathrm{GO}}\mathbf{X}_{\mathrm{KMER}}\mathbf{X}_{\mathrm{RG}}$	0.900	0.846	0.906	0.914	0.821	0.926	0.938	0.933	0.820	0.970
$\mathbf{X}_{\mathrm{CLIP}}\mathbf{X}_{\mathrm{KMER}}\mathbf{X}_{\mathrm{RNA}}\mathbf{X}_{\mathrm{RG}}$	**0.927**	0.724	0.928	**0.932**	0.731	**0.958**	**0.964**	0.948	0.777	0.973
$\mathbf{X}_{\mathrm{CLIP}}\mathbf{X}_{\mathrm{GO}}\mathbf{X}_{\mathrm{RNA}}\mathbf{X}_{\mathrm{RG}}$	0.923	**0.851**	0.913	0.915	0.822	0.942	0.956	0.865	0.816	0.983
$\mathbf{X}_{\mathrm{GO}}\mathbf{X}_{\mathrm{KMER}}\mathbf{X}_{\mathrm{RNA}}\mathbf{X}_{\mathrm{RG}}$	0.906	0.822	0.913	0.905	0.783	0.940	0.951	0.924	0.824	0.963
$\mathbf{X}_{\mathrm{CLIP}}\mathbf{X}_{\mathrm{GO}}\mathbf{X}_{\mathrm{KMER}}\mathbf{X}_{\mathrm{RNA}}\mathbf{X}_{\mathrm{RG}}$	0.924	0.849	0.917	0.924	**0.825**	0.944	0.956	0.929	**0.828**	0.973

Supplementary Table 7: Area under ROC curve for all combinations of RBP experiments and data sources subsets.

Subset	[11] ELAVL1	[12] ESWR1	[13] FUS	[14] Mut FUS	[15] IGF2BP1-3	[16] hnRNPC	[17] hnRNPC	[18] hnRNPL	[19] hnRNPL	[20] hnRNPL-like
$\mathbf{X}_{\text{CLIP}}$	0.918	0.783	0.633	0.893	0.808	0.603	0.597	0.733	0.656	0.685
$\mathbf{X}_{\text{GO}}$	0.555	0.498	0.499	0.510	0.508	0.399	0.402	0.487	0.519	0.514
$\mathbf{X}_{\text{KMER}}$	0.690	0.749	0.761	0.724	0.570	0.768	0.970	0.627	0.614	0.599
$\mathbf{X}_{\text{RNA}}$	0.888	0.737	0.573	0.862	0.885	0.509	0.511	0.708	0.666	0.712
$\mathbf{X}_{\text{RG}}$	0.930	0.604	0.627	0.769	0.758	0.734	0.724	0.613	0.600	0.586
$\mathbf{X}_{\text{CLIP}}\mathbf{X}_{\text{GO}}$	0.928	0.760	0.607	0.886	0.782	0.491	0.482	0.706	0.646	0.660
$\mathbf{X}_{\text{CLIP}}\mathbf{X}_{\text{KMER}}$	0.956	0.863	0.798	0.930	0.699	0.952	0.973	0.788	0.713	0.728
$\mathbf{X}_{\text{CLIP}}\mathbf{X}_{\text{RNA}}$	0.922	0.808	0.624	0.931	0.910	0.580	0.569	0.760	0.693	0.736
$\mathbf{X}_{\text{CLIP}}\mathbf{X}_{\text{RG}}$	0.968	0.649	0.664	0.941	0.928	0.755	0.721	0.669	0.622	0.612
$\mathbf{X}_{\text{GO}}\mathbf{X}_{\text{KMER}}$	0.883	0.795	0.807	0.804	0.613	0.952	0.974	0.697	0.697	0.669
$\mathbf{X}_{\text{KMER}}\mathbf{X}_{\text{RNA}}$	0.762	0.763	0.773	0.766	0.698	0.949	0.971	0.658	0.670	0.678
$\mathbf{X}_{\text{KMER}}\mathbf{X}_{\text{RG}}$	0.965	0.831	0.782	0.910	0.875	0.945	0.970	0.761	0.767	0.679
$\mathbf{X}_{\text{GO}}\mathbf{X}_{\text{RNA}}$	0.864	0.662	0.554	0.823	0.852	0.424	0.430	0.668	0.627	0.665
$\mathbf{X}_{\text{GO}}\mathbf{X}_{\text{RG}}$	0.975	0.770	0.638	0.868	0.904	0.735	0.724	0.768	0.704	0.767
$\mathbf{X}_{\text{RNA}}\mathbf{X}_{\text{RG}}$	0.952	0.574	0.628	0.796	0.911	0.737	0.726	0.612	0.604	0.587
$\mathbf{X}_{\text{CLIP}}\mathbf{X}_{\text{GO}}\mathbf{X}_{\text{KMER}}$	0.974	0.872	0.812	0.938	0.791	0.956	0.975	0.770	0.748	0.702
$\mathbf{X}_{\text{CLIP}}\mathbf{X}_{\text{KMER}}\mathbf{X}_{\text{RNA}}$	0.963	0.866	0.811	**0.956**	0.821	0.955	0.973	0.801	0.784	0.747
$\mathbf{X}_{\text{CLIP}}\mathbf{X}_{\text{KMER}}\mathbf{X}_{\text{RG}}$	0.971	0.893	0.811	0.955	0.936	0.942	0.976	**0.826**	**0.801**	0.796
$\mathbf{X}_{\text{CLIP}}\mathbf{X}_{\text{GO}}\mathbf{X}_{\text{RNA}}$	0.960	0.816	0.609	0.937	0.920	0.484	0.477	0.753	0.698	0.722
$\mathbf{X}_{\text{CLIP}}\mathbf{X}_{\text{GO}}\mathbf{X}_{\text{RG}}$	**0.983**	0.834	0.702	0.933	0.926	0.780	0.770	0.794	0.754	0.786
$\mathbf{X}_{\text{CLIP}}\mathbf{X}_{\text{RNA}}\mathbf{X}_{\text{RG}}$	0.968	0.765	0.675	0.950	0.939	0.754	0.729	0.689	0.621	0.590
$\mathbf{X}_{\text{GO}}\mathbf{X}_{\text{KMER}}\mathbf{X}_{\text{RNA}}$	0.947	0.809	0.813	0.825	0.705	0.952	0.974	0.732	0.734	0.724
$\mathbf{X}_{\text{GO}}\mathbf{X}_{\text{KMER}}\mathbf{X}_{\text{RG}}$	0.967	0.833	0.821	0.890	0.918	0.955	0.973	0.795	0.785	0.776
$\mathbf{X}_{\text{KMER}}\mathbf{X}_{\text{RNA}}\mathbf{X}_{\text{RG}}$	0.965	0.834	0.782	0.929	0.922	0.932	0.975	0.770	0.783	0.769
$\mathbf{X}_{\text{GO}}\mathbf{X}_{\text{RNA}}\mathbf{X}_{\text{RG}}$	0.969	0.785	0.637	0.900	0.921	0.738	0.734	0.783	0.744	0.779
$\mathbf{X}_{\text{CLIP}}\mathbf{X}_{\text{GO}}\mathbf{X}_{\text{KMER}}\mathbf{X}_{\text{RNA}}$	0.971	0.886	**0.832**	0.955	0.916	0.955	0.975	0.794	0.764	0.769
$\mathbf{X}_{\text{CLIP}}\mathbf{X}_{\text{GO}}\mathbf{X}_{\text{KMER}}\mathbf{X}_{\text{RG}}$	0.978	0.846	0.817	0.933	0.931	**0.958**	**0.978**	0.810	0.786	0.791
$\mathbf{X}_{\text{CLIP}}\mathbf{X}_{\text{KMER}}\mathbf{X}_{\text{RNA}}\mathbf{X}_{\text{RG}}$	0.969	**0.894**	0.829	0.954	**0.944**	0.952	0.975	0.822	0.790	**0.801**
$\mathbf{X}_{\text{CLIP}}\mathbf{X}_{\text{GO}}\mathbf{X}_{\text{RNA}}\mathbf{X}_{\text{RG}}$	0.979	0.830	0.715	0.945	0.934	0.779	0.755	0.799	0.742	0.778
$\mathbf{X}_{\text{GO}}\mathbf{X}_{\text{KMER}}\mathbf{X}_{\text{RNA}}\mathbf{X}_{\text{RG}}$	0.968	0.836	0.796	0.906	0.930	0.926	0.970	0.769	0.761	0.776
$\mathbf{X}_{\text{CLIP}}\mathbf{X}_{\text{GO}}\mathbf{X}_{\text{KMER}}\mathbf{X}_{\text{RNA}}\mathbf{X}_{\text{RG}}$	0.980	0.865	0.776	0.950	0.940	0.953	0.976	0.795	0.785	0.788

Supplementary Table 8: Area under ROC curve for all combinations of RBP experiments and data sources subsets (continued).

Subset	[21] MOV10	[22] Nsun2	[23] PUM2	[24] QKI	[25] SRSF1	[26] TAF15	[27] TDP-43	[28] TIA1	[29] TIAL1	[30] U2AF2	[31] U2AF2 (KD)
$\mathbf{X}_{\text{CLIP}}$	0.879	0.676	0.812	0.585	0.700	0.843	0.512	0.839	0.811	0.632	0.650
$\mathbf{X}_{\text{GO}}$	0.496	0.531	0.511	0.521	0.515	0.516	0.529	0.478	0.334	0.372	0.505
$\mathbf{X}_{\text{KMER}}$	0.652	0.715	0.746	0.845	0.718	0.768	0.696	0.682	0.706	0.754	0.695
$\mathbf{X}_{\text{RNA}}$	0.882	0.761	0.836	0.536	0.874	0.728	0.480	0.780	0.713	0.554	0.593
$\mathbf{X}_{\text{RG}}$	0.792	0.554	0.758	0.658	0.679	0.584	0.729	0.870	0.734	0.695	0.673
$\mathbf{X}_{\text{CLIP}}\mathbf{X}_{\text{GO}}$	0.861	0.668	0.795	0.582	0.661	0.842	0.540	0.798	0.699	0.504	0.632
$\mathbf{X}_{\text{CLIP}}\mathbf{X}_{\text{KMER}}$	0.907	0.823	0.936	0.901	0.787	0.897	0.733	0.928	0.907	0.818	0.795
$\mathbf{X}_{\text{CLIP}}\mathbf{X}_{\text{RNA}}$	0.924	0.798	0.872	0.572	0.878	0.838	0.492	0.858	0.821	0.647	0.679
$\mathbf{X}_{\text{CLIP}}\mathbf{X}_{\text{RG}}$	0.933	0.709	0.902	0.677	0.852	0.767	0.739	0.905	0.892	0.720	0.692
$\mathbf{X}_{\text{GO}}\mathbf{X}_{\text{KMER}}$	0.684	0.779	0.834	0.912	0.761	0.835	0.868	0.821	0.820	0.878	0.837
$\mathbf{X}_{\text{KMER}}\mathbf{X}_{\text{RNA}}$	0.731	0.720	0.879	0.902	0.745	0.790	0.758	0.769	0.725	0.819	0.786
$\mathbf{X}_{\text{KMER}}\mathbf{X}_{\text{RG}}$	0.926	0.834	0.943	0.866	0.888	0.816	0.838	0.935	0.912	0.909	0.876
$\mathbf{X}_{\text{GO}}\mathbf{X}_{\text{RNA}}$	0.841	0.716	0.752	0.546	0.812	0.690	0.521	0.709	0.557	0.423	0.575
$\mathbf{X}_{\text{GO}}\mathbf{X}_{\text{RG}}$	0.896	0.702	0.869	0.662	0.873	0.754	0.759	0.875	0.849	0.796	0.796
$\mathbf{X}_{\text{RNA}}\mathbf{X}_{\text{RG}}$	0.908	0.556	0.842	0.657	0.708	0.646	0.729	0.787	0.693	0.713	0.696
$\mathbf{X}_{\text{CLIP}}\mathbf{X}_{\text{GO}}\mathbf{X}_{\text{KMER}}$	0.896	0.846	0.931	0.847	0.800	0.905	0.859	0.918	0.911	0.909	0.892
$\mathbf{X}_{\text{CLIP}}\mathbf{X}_{\text{KMER}}\mathbf{X}_{\text{RNA}}$	0.944	0.840	0.945	**0.912**	0.806	0.909	0.808	0.937	0.920	0.851	0.821
$\mathbf{X}_{\text{CLIP}}\mathbf{X}_{\text{KMER}}\mathbf{X}_{\text{RG}}$	0.956	0.868	**0.961**	0.877	0.898	**0.910**	0.860	0.943	**0.942**	0.915	**0.925**
$\mathbf{X}_{\text{CLIP}}\mathbf{X}_{\text{GO}}\mathbf{X}_{\text{RNA}}$	0.932	0.748	0.875	0.574	0.878	0.849	0.523	0.841	0.756	0.528	0.651
$\mathbf{X}_{\text{CLIP}}\mathbf{X}_{\text{GO}}\mathbf{X}_{\text{RG}}$	0.933	0.779	0.897	0.671	0.881	0.879	0.753	0.907	0.897	0.832	0.821
$\mathbf{X}_{\text{CLIP}}\mathbf{X}_{\text{RNA}}\mathbf{X}_{\text{RG}}$	0.950	0.697	0.906	0.683	0.849	0.824	0.738	0.914	0.896	0.780	0.737
$\mathbf{X}_{\text{GO}}\mathbf{X}_{\text{KMER}}\mathbf{X}_{\text{RNA}}$	0.854	0.811	0.893	0.889	0.824	0.845	0.869	0.874	0.880	0.908	0.884
$\mathbf{X}_{\text{GO}}\mathbf{X}_{\text{KMER}}\mathbf{X}_{\text{RG}}$	0.932	0.744	0.928	0.831	0.884	0.826	**0.895**	0.919	0.889	0.926	0.901
$\mathbf{X}_{\text{KMER}}\mathbf{X}_{\text{RNA}}\mathbf{X}_{\text{RG}}$	0.947	0.840	0.952	0.871	0.884	0.827	0.845	0.941	0.927	0.906	0.879
$\mathbf{X}_{\text{GO}}\mathbf{X}_{\text{RNA}}\mathbf{X}_{\text{RG}}$	0.926	0.782	0.881	0.665	0.896	0.795	0.734	0.886	0.866	0.820	0.804
$\mathbf{X}_{\text{CLIP}}\mathbf{X}_{\text{GO}}\mathbf{X}_{\text{KMER}}\mathbf{X}_{\text{RNA}}$	0.941	**0.872**	0.933	0.846	0.880	0.895	0.872	0.933	0.903	0.908	0.902
$\mathbf{X}_{\text{CLIP}}\mathbf{X}_{\text{GO}}\mathbf{X}_{\text{KMER}}\mathbf{X}_{\text{RG}}$	0.950	0.794	0.924	0.797	0.891	0.880	0.891	0.918	0.917	0.908	0.899
$\mathbf{X}_{\text{CLIP}}\mathbf{X}_{\text{KMER}}\mathbf{X}_{\text{RNA}}\mathbf{X}_{\text{RG}}$	**0.959**	0.869	0.956	0.868	**0.913**	0.900	0.858	**0.949**	0.931	**0.933**	0.904
$\mathbf{X}_{\text{CLIP}}\mathbf{X}_{\text{GO}}\mathbf{X}_{\text{RNA}}\mathbf{X}_{\text{RG}}$	0.949	0.794	0.902	0.686	0.898	0.875	0.742	0.905	0.893	0.830	0.820
$\mathbf{X}_{\text{GO}}\mathbf{X}_{\text{KMER}}\mathbf{X}_{\text{RNA}}\mathbf{X}_{\text{RG}}$	0.941	0.760	0.932	0.834	0.898	0.836	0.893	0.903	0.879	0.872	0.895
$\mathbf{X}_{\text{CLIP}}\mathbf{X}_{\text{GO}}\mathbf{X}_{\text{KMER}}\mathbf{X}_{\text{RNA}}\mathbf{X}_{\text{RG}}$	0.955	0.793	0.930	0.842	0.909	0.896	0.893	0.911	0.885	0.903	0.901

Supplementary Table 9: Area under ROC curve for all combinations of RBP experiments and data sources subsets (continued).

6 Importance of individual data sources

Assesing the importance of individual data sources in Suppl. Tables 7-9 is non-trivial since the scores obtained by a particular subsets are not independent. Nevertheless, we transformed each column of Suppl. Tables 7-9 in a 31×5 binary matrix $\mathbf{B}$, corresponding to 31 subsets of 5 data sources. For each data source, we calculate the Spearman correlation coefficient between: its corresponding column in $\mathbf{B}$ and column with AUC scores. Thus, we obtain a 5×1 vector containing correlation coefficients for each protein, which we use to perform hierarchical clustering in Suppl. Figure 5.

We observe clear influence of $\mathbf{X}_{\mathrm{KMER}}$on sequence-dependent proteins (TIA1/TIAL1, PUM2, hnRNPs, U2AF2, FUS, etc.), while the influence is less pronounce for more region-type dependent proteins, such as those displaying bias towards introns and 3'UTRS (ELAVL, Uren *et al.* (2011)), or in both exons and introns (SRSF1, Aenkoe *et al.* (2012)). On the other hand, RNA structure is most informative data source for eIF4AIII, which binds unstructured RNA (Saulière *et al.* (2012)). Indeed, this can be observed on plots of individual feature vectors (Suppl. Section 8.3), where the first component is associated to $> 70\%$ of positive nucleotides and appears with no distinctive structure.

Interestingly, all experiments performed using iCLIP show strong RNA k-mer preference, which is attriuted to the individual nucleotide resolution. Protocols with lower resolution, such as CLIPSEQ, conversely are not correlated with RNA k-mers, but are rather modelled by more coarse data sources such as region type and RNAfold.

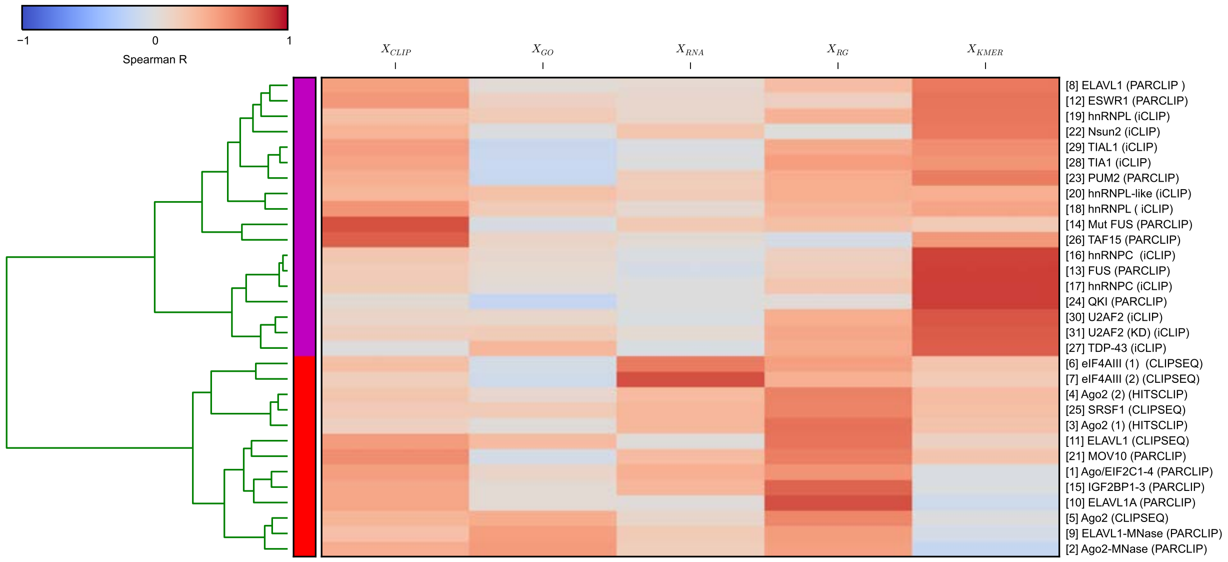

Supplementary Fig. 5: Hierarchical clustering of proteins based on the importance of each individual data source.

7 Clustering of RBPs based on individual data sources

Here, we examine values of features in the coefficient matrices $\mathbf{H}_i$ for each RBP experiment. By comparing the magnitude of individual features in the modules related to positive samples (cross-link sites), we identify features responsible for RBP binding on the target sites. Detailed plots on vectors in the coefficient matrices, on which the clustering is based are displayed in Suppl. Section 8.

For each RBP experiment, we select the most relevant module (see main text) and normalize the corresponging row vector in $\mathbf{H}_i$ by converting it to z-scores. We run hierarchical clustering (Ward's linkage) on the row vectors and display the results are heatmaps for each data source.

7.1 RNA k-mers ($\mathbf{H}_{\mathrm{KMER}}$)

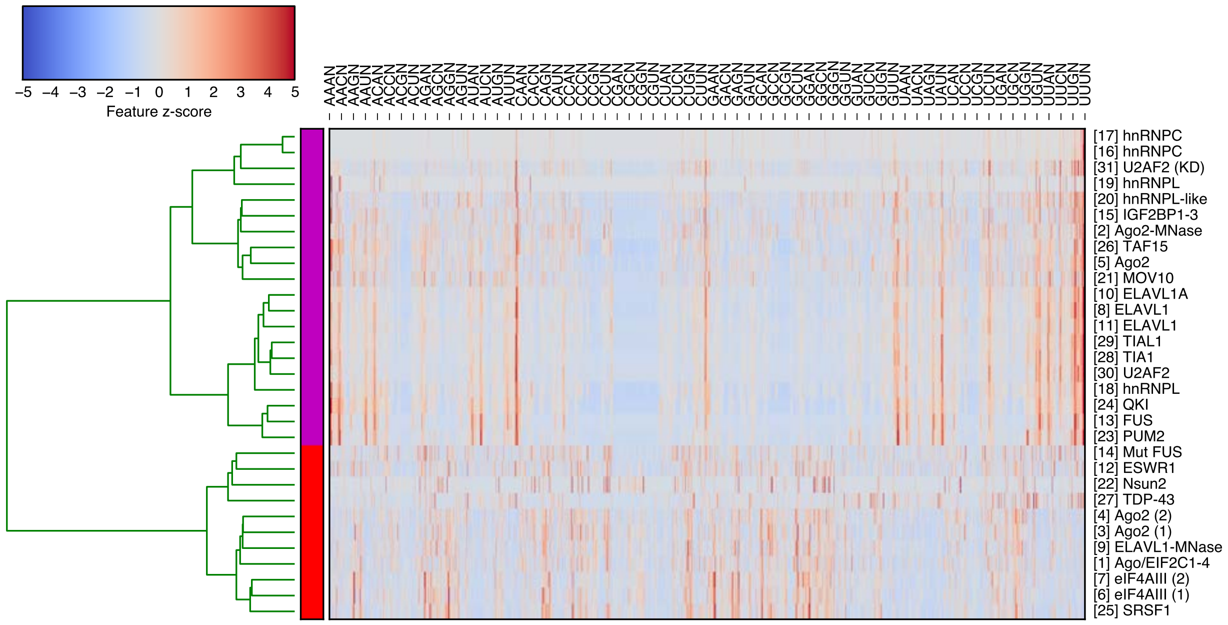

Supplementary Fig. 6: Protein similarity based on RNA k-mer row vectors in $\mathbf{H}_{\mathrm{KMER}}$. Features represent all possible kmers within the interval [-50..50] relative to the cross-link sites, resulting in $101 \times 256 = 25856$ features. To avoid clutter, only the centers and first three nucleotides of the 4-mer are displayed.

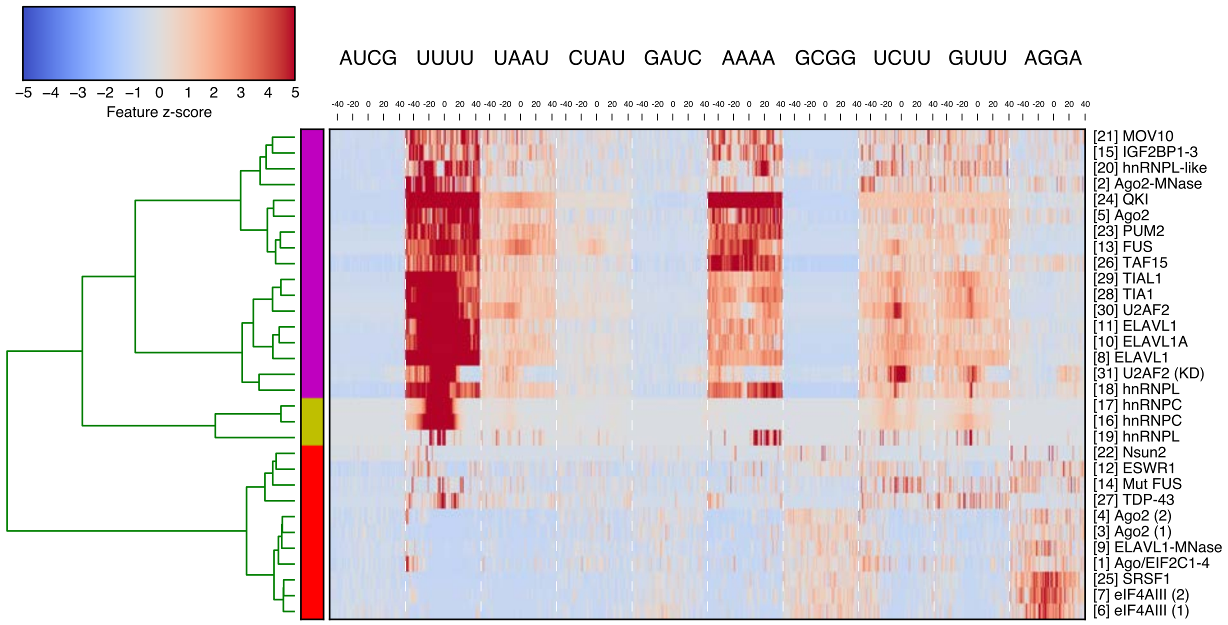

Supplementary Fig. 7: Protein similarity based on RNA k-mer row vectors in $\mathbf{H}_{\mathrm{KMER}}$. K-means clustering is performed on row vectors from top modules for each of the RBP experiments. Ten k-mers closest to the centroid vectors were selected. The z-scores within the intervals [-50..50] nucleotides are displayed.

7.2 RNA secondary structure ($\mathbf{H}_{\mathrm{RNA}}$)

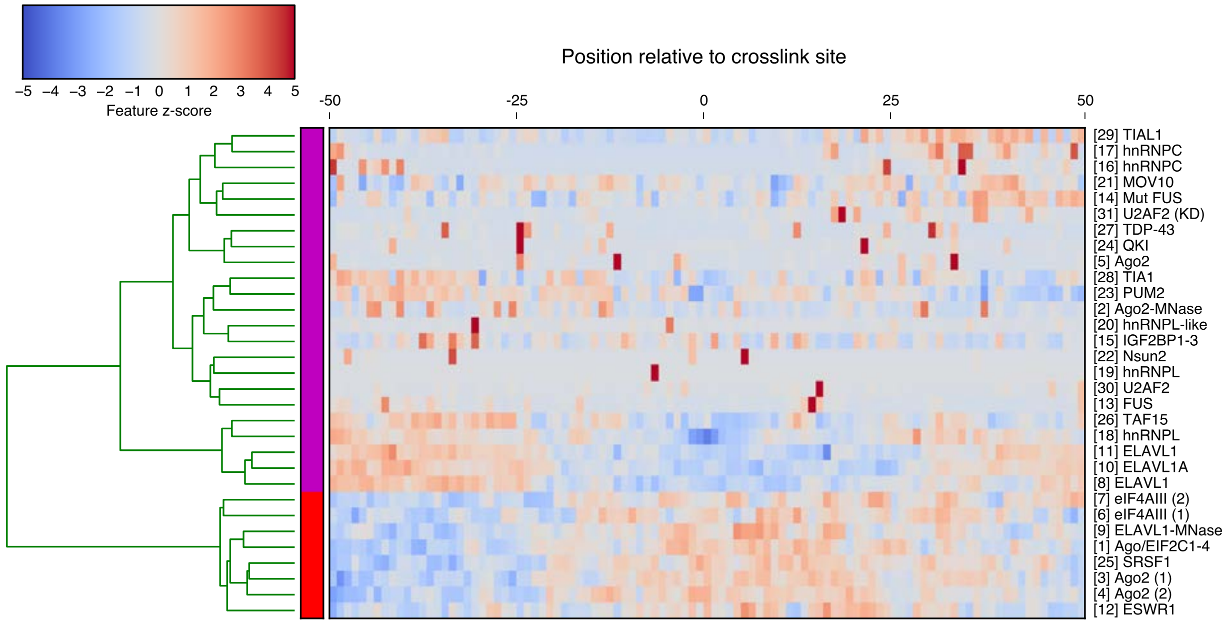

Supplementary Fig. 8: Protein similarity based on RNA secondary structure row vectors in $\mathbf{H}_{\mathrm{RNA}}$. Z-scores of features obtained via RNAfold output are proportional to the predicted probability of double-stranded RNA at the particular nucleotide within the [-50..50] interval relative to the cross-link sites.

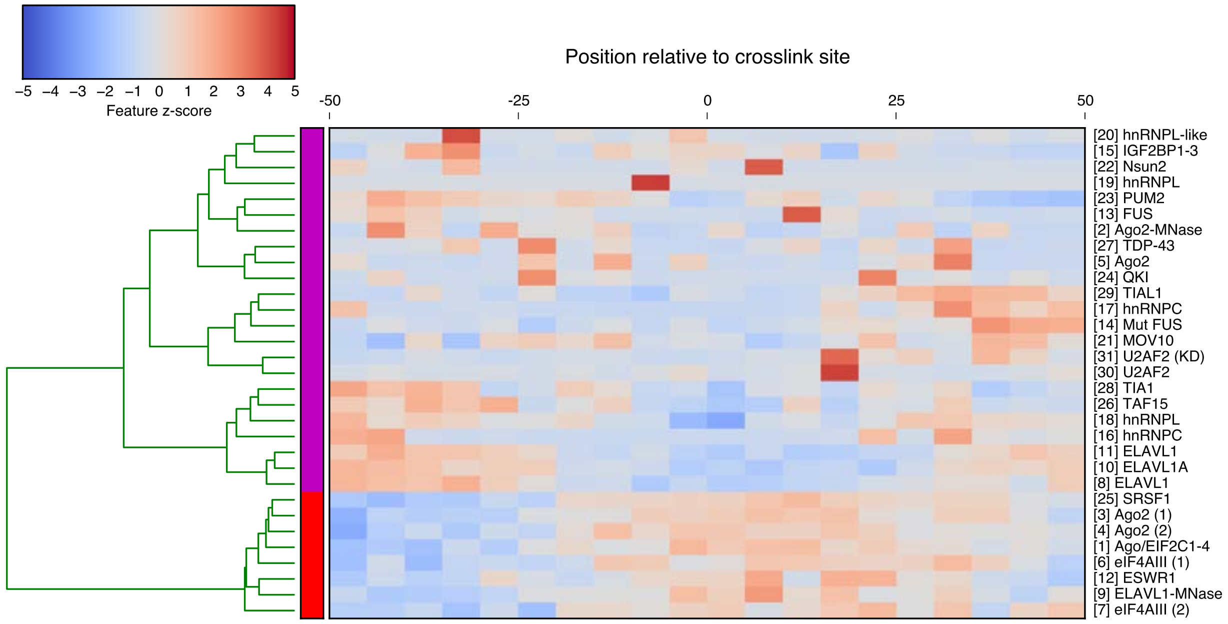

Supplementary Fig. 9: Protein similarity based on RNA secondary structure row vectors in $\mathbf{H}_{\mathrm{RNA}}$. Z-scores of features obtained via RNAfold output are proportional to the predicted probability of double-stranded RNA at the particular nucleotide within the [-50..50] interval relative to the cross-link sites. Scores within 5 nucleotide bins were summed.

7.3 Experimental cDNA counts ($\mathbf{H}_{\text{CLIP}}$)

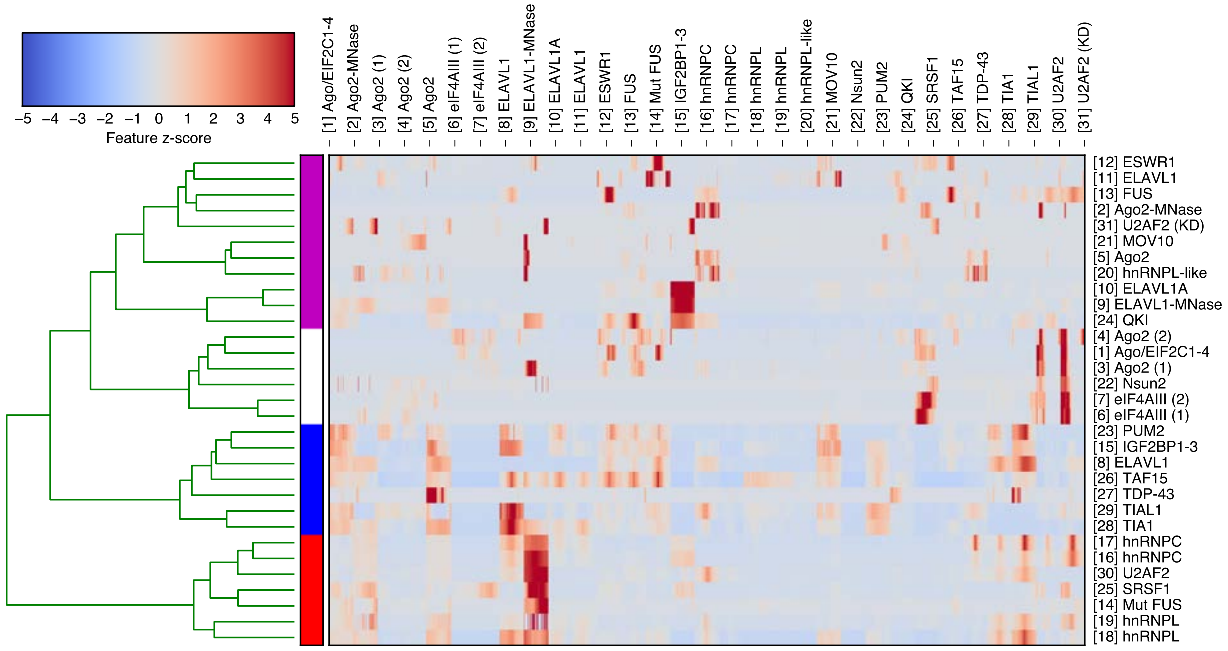

Supplementary Fig. 10: Protein similarity based on cDNA counts row vectors in $\mathbf{H}_{\text{CLIP}}$. Note that the values for RBPs in the same groups are zero in order not to bias the clustering.

7.4 Genomic region types ($\mathbf{H}_{\text{RG}}$)

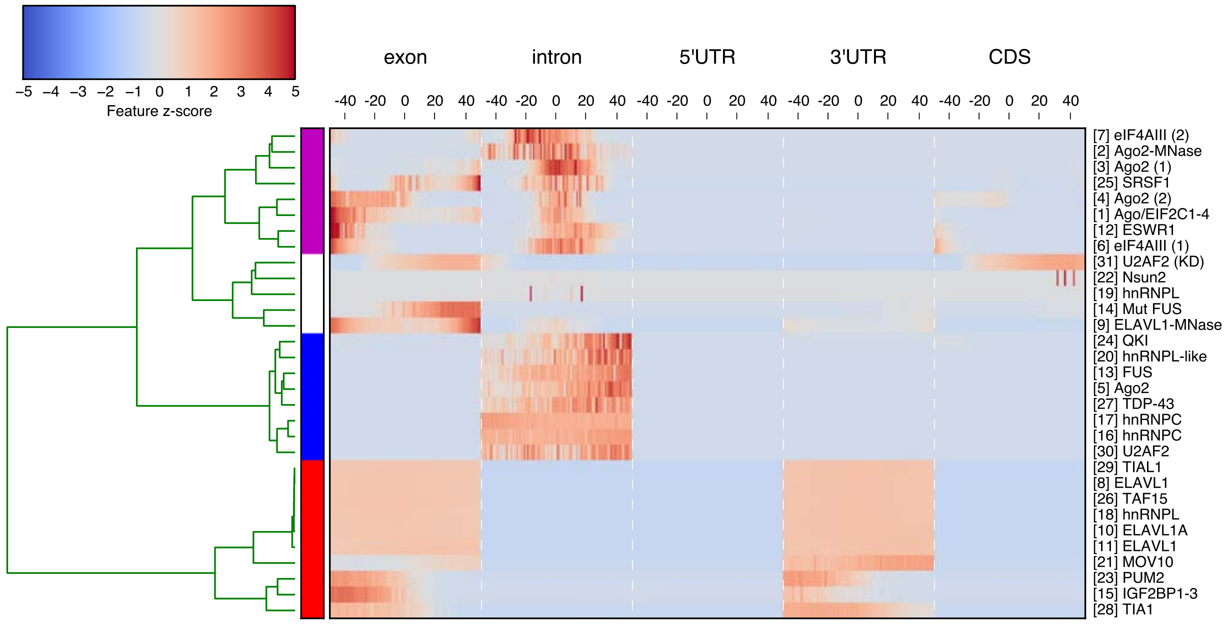

Supplementary Fig. 11: Protein similarity based on genomic region types row vectors in $\mathbf{H}_{\text{RG}}$. For each region type, the interval [-50..50] relative to the cross-link sites is shown.

7.5 Gene associations ($\mathbf{H}_{\mathrm{GO}}$)

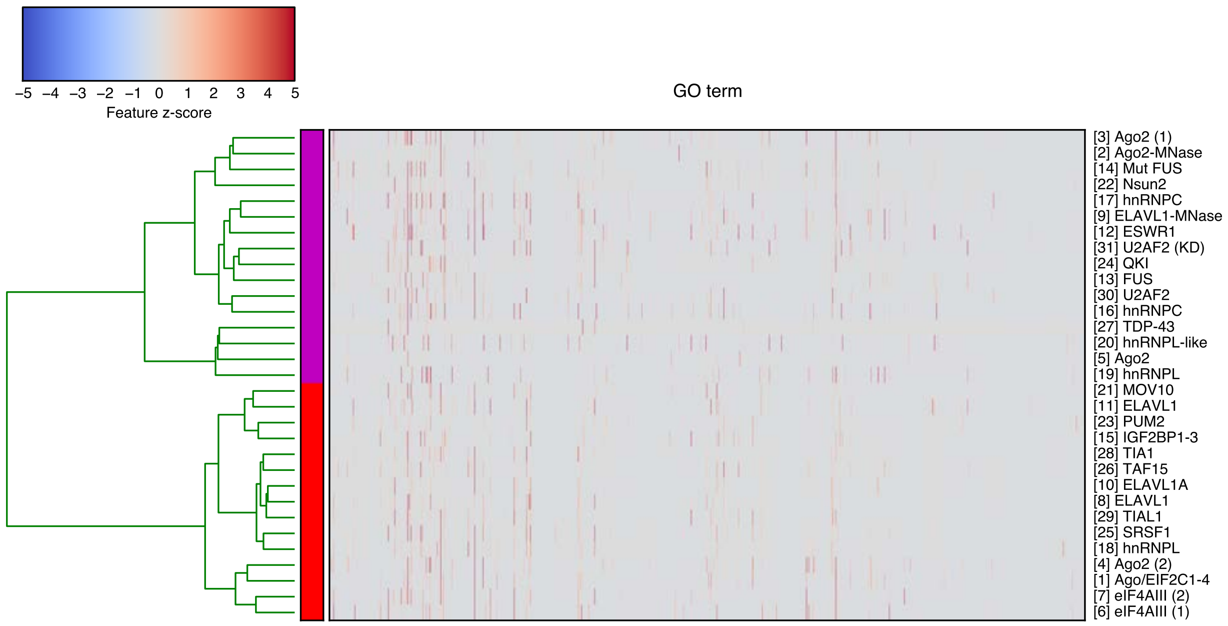

Supplementary Fig. 12: Protein similarity based on row vectors in $\mathbf{H}_{\mathrm{GO}}$, representing gene associations within GeneOntology (GO) annotation.

7.6 Discovery of RNA motifs

RBP binding is dependent on both positioning and sequence content of motifs; these are encoded in $\mathbf{X}_{\mathrm{KMER}}$, where each column represents the presence of a specific RNA k-mer at a specific offset from the cross-linked site. Because we scan for k-mers within w nucleotides, this gives $W \cdot 4^k$ columns (in our case, $k = 4$ and $W = 101$). To alleviate the exponential increase in the number of columns with increasing k, we use a heuristic approach based on the learned factor models for extracting complex motifs of arbitrary length from k-mer frequency and positional information, similar to Hutchins et al. (Hutchins *et al.*, 2008). Our approach differs in that it uses all other data sources on additional circumstantial evidence besides k-mer frequency to aid the identification of the sequence motifs and the positional distribution associated with protein binding.

Upon discovery of modules most associated with positions of a specific selected RBP (Section 2.5 in main text), associate the positions beloning to module as the *positive set* $\mathcal{P}$. The vector in $\mathbf{H}_{\mathrm{KMER}}$ corresponding to the module is termed $\mathbf{h}$. To improve specificity, we retain only the 5% highest elements of $\mathbf{h}$ by setting other elements to zero. The vector $\mathbf{h}$ vector is then interpreted as an estimate of the probability distribution of k-mers within sequences associated to the set $\mathcal{P}$. We use a background probability distribution for nucleotide n is given by $P_{exp}(n) \sim$ (A:0.3, C:0.2, G:0.2, T:0.3).

For a given module, we randomly initialize a estimated motif count matrix $\mathbf{M} \in \mathbb{R}^{4 \times L}$, where columns represent nucleotide counts (probabilities) at each of L positions within the motif. For simplicity, the rows of $\mathbf{M}$ are indexed by nucleotides, $\mathbf{M}(A,:) = \mathbf{M}(0,:)$, $\mathbf{M}(C,:) = \mathbf{M}(1,:)$, etc.

At each iteration, $\mathbf{M}$ is scored the log-probability that the observed distribution is generated by $\mathbf{M}$. Define a following function to score a motif M represented a a positional count matrix against the positions in the positive set $\mathcal{P}$.

Supplementary Function 1 Estimation of complex motifs

Input: estimated motif count matrix $\mathbf{M} \in \mathbb{R}^{4 \times L}$, set $\mathcal{P}$, expected nucleotide probability distribution P_{exp}.
Output: Motif log odds comparing to the expected distribution.

function scoremotif($\mathbf{M}$, $\mathcal{P}$, P_{exp}):
  for $l = 1..L$: $\mathbf{M}(:,l) = \mathbf{M}(:,l) / \sum_{n \in \{A,C,T,G\}} \mathbf{M}(n,l)$
  $p_e = \prod_{l=1}^{L} \sum_{n \in \{A,C,T,G\}} P_{\mathrm{exp}}(n) \cdot \mathbf{M}(n,l)$
  $p_o = \max_{w=1}^{W-L+1} \sum_{s \in \mathcal{P}} \prod_{l=1}^{L} \sum_{n \in \{A,C,G,T\}} s(w+l) \cdot \mathbf{M}(n,l)$
  return $\log(p_o)$ - $\log(p_e)$

Consider Suppl. Function 1. The positions within the motif at index l are first normalized to obtain an estimate of the probabilities. The motif $\mathbf{M}$ is then scored against the expected distribution P_{exp} to get p_e. The observed probability distribution is defined as the maximum probability within the nucleotide regions at positions $w = 1...W - L + 1$, averaged over all sequences $s \in \{A, C, T, G\}^W$ corresponding to the positions in the positive set $\mathcal{P}$. The returned score is the log odds ratio $\log \frac{p_o}{p_e}$.

Until convergence, k-mers are sampled from the distribution given by $\mathbf{h}$ and $\mathbf{M}$ is updated at columns j:$(j + k - 1)$ to give $\mathbf{M}'$. At each iteration, $\mathbf{M}$ is updated at positions $(j + 1)$ mod $(L - k + 1)$ to ensure equal update frequency for all positions within the motif. The update is accepted if the log-probability of the updated $\mathbf{M}$ increases comparing to the previous iteration. The procedure is summarized in Suppl. Algorithm 2.

Supplementary Algorithm 2 Estimation of complex motifs

Input: feature vector $\mathbf{h}$, set $\mathcal{P}$, expected nucleotide probability distribution P_{exp}, initial pseudocount $C \in \mathbb{N}$.
Output: estimated motif count matrix $\mathbf{M} \in \mathbb{N}^{4 \times l}$

Initialize $\mathbf{M} \sim C \cdot \mathcal{U}[0,1]^{4 \times l}$
until convergence:
  for $j = 1 : l - k + 1$
    Let $\mathbf{k} \sim$ sample a k-mer according to the distribution given by $\mathbf{h}$.
    Let $\mathbf{M}$' equal $\mathbf{M}$ updated with $\mathbf{k}$ at positions $(j + 1)$ mod $(L - k + 1)$.
    s_M = scoremotif($\mathbf{M}$, $\mathcal{P}$, P_{exp})
    s_M = scoremotif($\mathbf{M}'$, $\mathcal{P}$, P_{exp})

In all our experiments, the pseudocount C was set to 20 and the estimation converged within 1000 iterations.

Using the algorithm described, we estimate longer, complex motifs. Four modules resulting in four motifs with highest probability were chosen for each protein. Ward's hierarchical clustering is performed and 50 motifs which result as centroids after K-means clustering. Heatmap displays the difference in observed frequency (at cross-link sites) versus expected probability (at random positions). Columns and rows were reordered using Ward's hierarchical clustering. Each motif is displayed with Weblogo software.

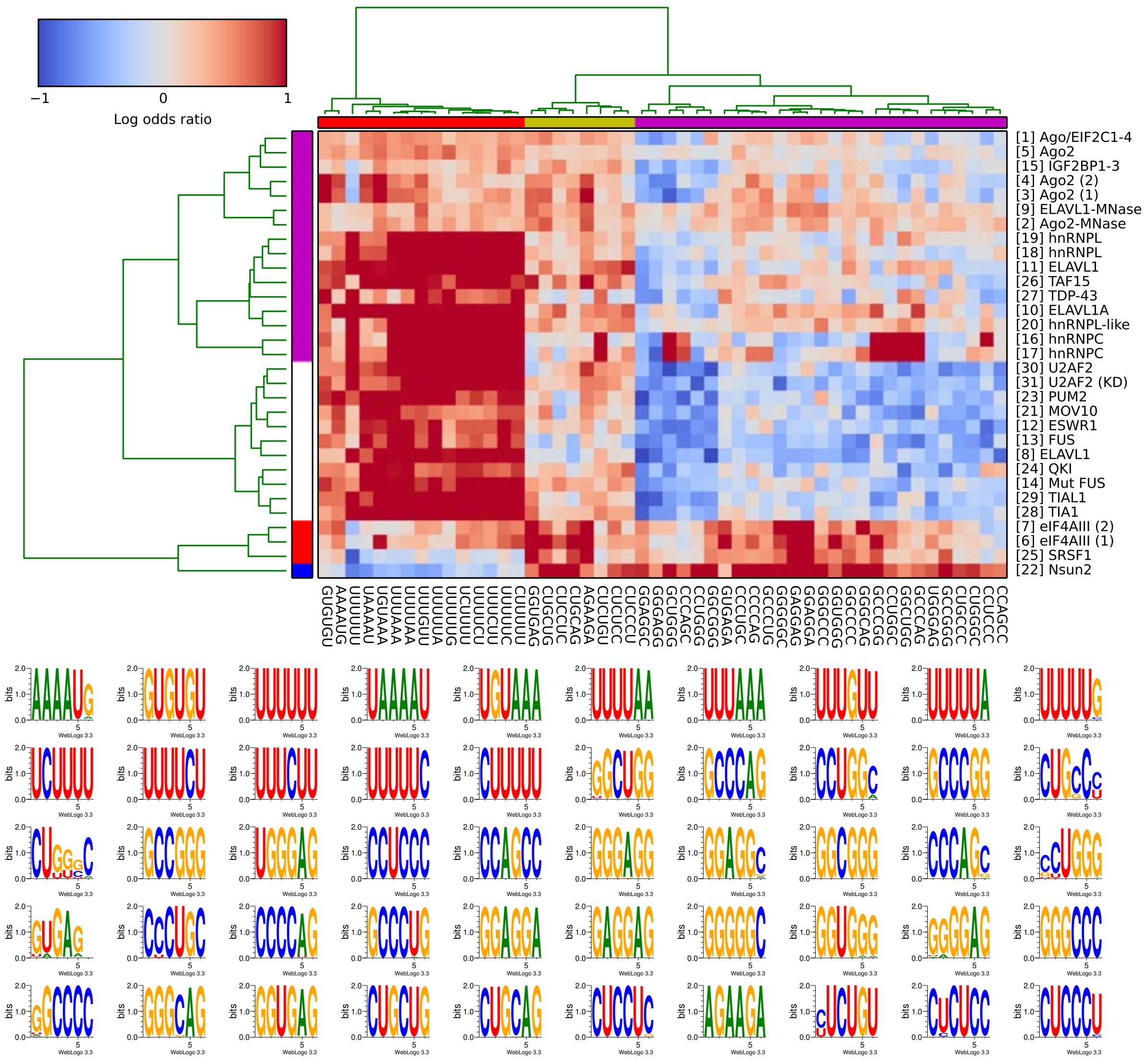

Supplementary Fig. 13: A survey of 50 most common complex motifs, estimated from basis vectors in $\mathbf{H}_{\mathrm{KMER}}$.

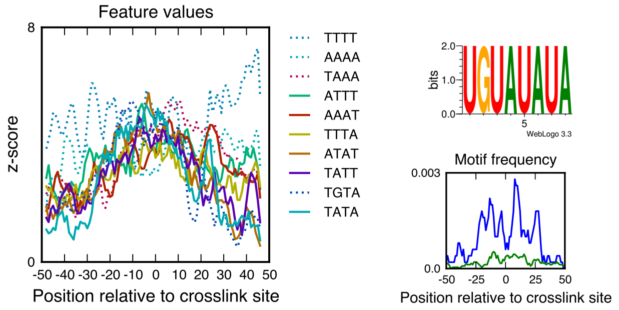

Supplementary Fig. 14: Example of complex motif estimation from row vectors in $\mathbf{H}_{\text{KMER}}$. (left) Visualization of feature values (row vectors in $\mathbf{H}_{\text{KMER}}$) of most related module to cross-link samples of PUM2 protein. (right) Complex motif derived from the row vector using procedure in Suppl. Section 7.6. The motif is similar to known motif UGUANAUA (Hafner *et al.*, 2010). The observed frequency of the derived motif in proximity to cross-link sites (blue) is greater than the expected probability at random positions within protein coding genes (green).

Protein	Predicted motif	Known motif	D	Source	ID
[8] ELAVL1	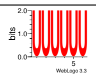	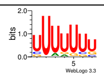	0	RNAcompete	M232_0.6
[11] ELAVL1	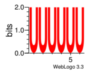	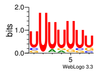	0	RNAcompete	M232_0.6
[13] FUS	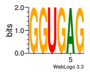	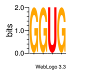	0	SELEX	M316_0.6
[17] hnRNPC	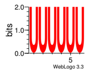	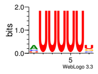	0	RNAcompete	M025_0.6
[18] hnRNPL	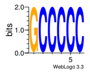	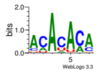	3	RNAcompete	M027_0.6
[19] hnRNPL	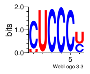	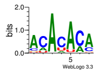	3	RNAcompete	M027_0.6
[24] QKI	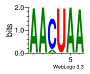	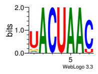	1	SELEX	M262_0.6
[25] SRSF1	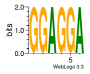	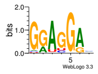	0	RNAcompete	M102_0.6
[26] TAF15	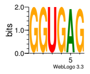	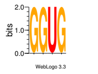	0	SELEX	M316_0.6
[28] TIA1	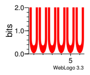	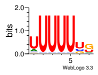	0	RNAcompete	M075_0.6
[30] U2AF2	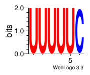	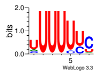	0	RNAcompete	M077_0.6
[31] U2AF2 (KD)	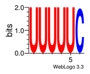	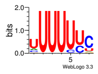	0	RNAcompete	M077_0.6

Supplementary Fig. 15: Levenshtein distance (D) between predicted complex RNA sequence motifs and motifs reported in (Ray *et al.*, 2013) for nine proteins included in both studies.

7.7 Sequences of Alu elements bound and regulated by hnRNPC

Investigation of known binding sites of [16] hnRNPC and [17] hnRNP C (König *et al.*, 2010; Zarnack *et al.*, 2013). As supported by Fig. 4a (see main text) and Supplementary Fig. 13, motifs GGCTGG, GCCCAG, CCTGCC, GCCGGG are associated with hnRNPC . These motifs commonly occur in antisense Alu elements next to the U-tract that directly interacts with hnRNPC (Fig. 16), which demonstrates that our algorithm can detect common neighbouring motifs, even if these are not part of the primary binding site. Consesus Alu elements sequences in human were scanned for motifs, where at least three motifs were identified in each consesus sequence (Supplementary Fig. 17).

```
ttttctttcttttctttttttttattttattttttttttgagacagGTTCTCGTCCTGTCA
CCCAGGCTGGTATGCGGTGGTGTGATCGTAGCTCACTGCAGTCTCGAACTCCTGGGTTCAA
GCGATCCTTCCACTTCAGCCTCCCAAGTAGCTGGTACTACAGgtgtgtgccacgacacccg
gctaagtttttgaaatttatttttttgtagagacaggattttcctatgttgcccaggctggt
tttcaaactcctggcc
```

Supplementary Fig. 16: Sequence of the CD55 alu element bound and regulated by hnRNPC, showing hnRNPC binding site (red), regulated exon (capital letters) and motifs associated with hnRNPC (underlined).

>HSU14567 Human Alu-J subfamily consensus sequence. (rev. complement)
TTTTTTTTGAGACAGGGTCTCGCTCTGTCGCCCAGGCTGGAGTGCAGTGGCGCGATCACGGCTCACTGCAGCCTCGACCTC
CCCGGGCTCAAGCGATCCTCCTGCCTCAGCCTCCCGAGTAGCTGGGACTACAGGCGCGCGCCACCACGCCCGGCTAATTTT
TTGTATTTTTTGTAGAGACGGGGTTTCACTATGTTGCCCAGGCTGGTCTCGAACTCCTGGGCTCAAGTGATCCTCCCGCCT
TCGGCCTCCCAAAGTGCTGGGATTACAGGCGTGAGCCACCGCGCCCGGCC

>HSU14573 Human Alu-Sq subfamily consensus sequence. (rev. complement)
TTTTTTTTGAGACGGAGTTTCGCTCTTGTTGCCCAGGCTGGAGTGCAGTGGCGCGATCTCGGCTCACTGCAACCTCCGCCT
TCCCGGGTTCAAGCGATTCTCCTGCCTCAGCCTCCCGAGTAGCTGGGATTACAGGCGCCCGCCACCACGCCCGGCTAATTT
TTTGTATTTTTAGTAGAGACGGGGTTTCACCATGTTGGCCAGGCTGGTCTCGAACTCCTGACCTCAGGTGATCCACCCGCC
CTCGGCCTCCCAAAGTGCTGGGATTACAGGCGTGAGCCACCGCGCCCGGCC

>HSU14568 Human Alu-Sb subfamily consensus sequence. (rev. complement)
TTTTTTTTGAGACGGAGTCTCGCTCTGTCGCCCAGGCTGGAGTGCAGTGGCGCGATCTCGGCTCACTGCAAGCTCCGCCTC
CCCGGGTTCACGCCATTCTCCTGCCTCAGCCTCCCGAGTAGCTGGGACTACAGGCGCCCGCCACCACGCCCGGCTAATTTT
TTGTATTTTTAGTAGAGACGGGGTTTCACCGTGTTAGCCAGGATGGTCTCGATCTCCTGACCTCGTGATCCGCCCGCCTCG
GGCCTCCCAAAGTGCTGGGATTACAGGCGTGAGCCACCGCGCCCGGCC

>HSU14572 Human Alu-Sp subfamily consensus sequence. (rev. complement)
TTTTTTTTGAGACGGAGTTTCGCTCTTGTTGCCCAGGCTGGAGTGCAATGGCGCGATCTCGGCTCACCGCAACCTCCGCCT
TCCCGGGTTCAAGCGATTCTCCTGCCTCAGCCTCCCGAGTAGCTGGGATTACAGGCATGCGCCACCACGCCCGGCTAATTT
TTTGTATTTTTAGTAGAGACGGGGTTTCTCCATGTTGGTCAGGCTGGTCTCGAACTCCCGACCTCAGGTGATCCGCCCGCC
CTCGGCCTCCCAAAGTGCTGGGATTACAGGCGTGAGCCACCGCGCCCGGCC

>HSU14571 Human Alu-Sc subfamily consensus sequence. (rev. complement)
TTTTTTTTGAGACGGAGTCTCGCTCTGTCGCCAGGCTGGAGTGCAGTGGCGCGATCTCGGCTCACTGCAACCTCCGCCTCC
CCGGGTTCAAGCGATTCTCCTGCCTCAGCCTCCCGAGTAGCTGGGACTACAGGCGCGCGCCACCACGCCCAGCTAATTTTT
TGTATTTTTAGTAGAGACGGGGTTTCACCATGTTGGCCAGGATGGTCTCGATCTCTTGACCTCGTGATCCGCCCGCCTCGG
GCCTCCCAAAGTGCTGGGATTACAGGCGTGAGCCACCGCGCCCGGCC

>HSU14574 Human Alu-Sx subfamily consensus sequence. (rev. complement)
TTTTTTTTGAGACGGAGTCTCGCTCTGTCGCCCAGGCTGGAGTGCAGTGGCGCGATCTCGGCTCACTGCAACCTCCGCCTC
CCCGGGTTCAAGCGATTCTCCTGCCTCAGCCTCCCGAGTAGCTGGGATTACAGGCGCGCGCCACCACGCCCGGCTAATTTT
TTGTATTTTTAGTAGAGACGGGGTTTCACCATGTTGGCCAGGCTGGTCTCGAACTCCTGACCTCAGGTGATCCGCCCGCCT
TCGGCCTCCCAAAGTGCTGGGATTACAGGCGTGAGCCACCGCGCCCGGCC

>HSU14569 Human Alu-Sb1 subfamily consensus sequence. (rev. complement)
TTTTTTTTGAGACGGAGTCTCGCTCTGTCGCCCAGGCTGGAGTGCAGTGGCGGGATCTCGGCTCACTGCAAGCTCCGCCTC
CCCGGGTTCACGCCATTCTCCTGCCTCAGCCTCCCAAGTAGCTGGGACTACAGGCGCCCGCCACTACGCCCGGCTAATTTT
TTGTATTTTTAGTAGAGACGGGGTTTCACCGTTTTAGCCGGGATGGTCTCGATCTCCTGACCTCGTGATCCGCCCGCCTCG
GGCCTCCCAAAGTGCTGGGATTACAGGCGTGAGCCACCGCGCCCGGCC

>HSU14570 Human Alu-Sb2 subfamily consensus sequence. (rev. complement)
TTTTTTTTGAGACGGAGTCTCGCTCTGTCGCCCAGGCCGGACTGCGGACTGCAGTGGCGCAATCTCGGCTCACTGCAAGCT
TCCGCTTCCCGGGTTCACGCCATTCTCCTGCCTCAGCCTCCCCAGTAGCTGGGACTACAGGCGCCCGCCACCGCGCCCGG
CTAATTTTTTGTATTTTTAGTAGAGACGGGGTTTCACCTTGTTAGCCAGGATGGTCTCGATCTCCTGACCTCATGATCCAC
CCCGCCTCGGCCTCCCAAAGTGCTGGGATTACAGGCGTGAGCCACCGCGCCCGGCC

Supplementary Fig. 17: Consesus sequences of Alu elements bound by hnRNPC in human. Reverse complements are displayed due to hnRNPC antisense binding. Identified motifs associated with hnRNPC are underlined.

8 Data source feature values

We present the most relevant row vectors in the coefficent matrices $\mathbf{H}_i$ for each RBP experiment. Top three row vectors associated with three most relevant modules for each RBP experiment are displayed. After normalization via the z-score transformation, we plot the values in the row vectors. For matrices associated with a high number of features that would cause clutter ($\mathbf{H}_{\mathrm{KMER}}$), at most five features are selected for each module. The bars above the plot represent the percentage of positions, covered by each module. The coverages can overlap (*i.e.*, the modules have non empty intersections) and are sorted by the percentage of new positions described (comparing to the top scoring module). For each RBP, orthogonality parameter α for model with best prediction performance according to AUC is reported.

8.1 RNA k-mers ($\mathbf{X}_{\mathbf{KMER}}$)

[1] Ago/EIF2C1-4 ($\alpha = 10^4$)

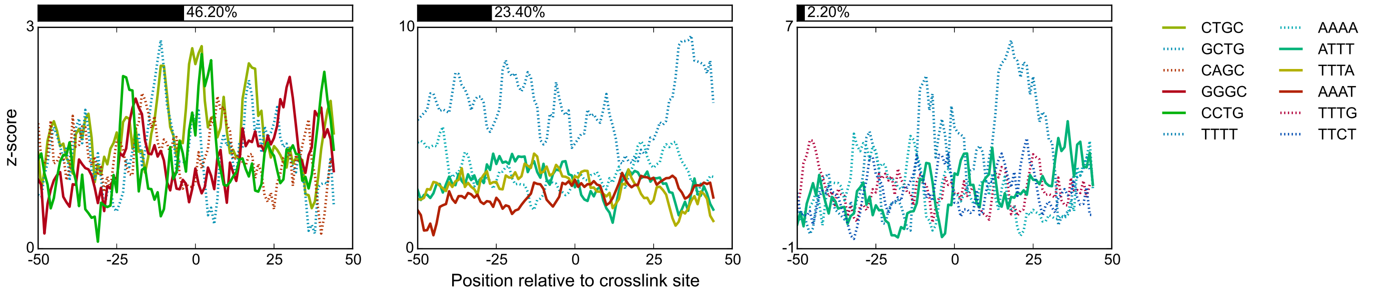

[2] Ago2-MNase ($\alpha = 10^3$)

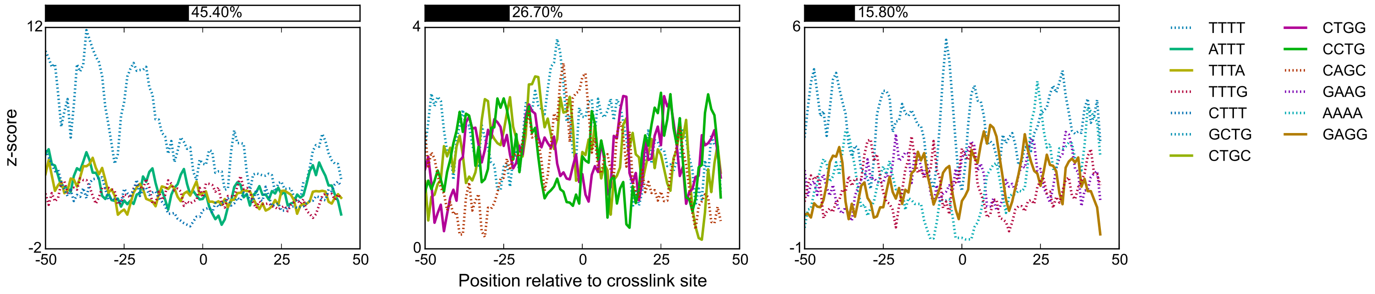

[3] Ago2 (1) ($\alpha = 10^{-1}$)

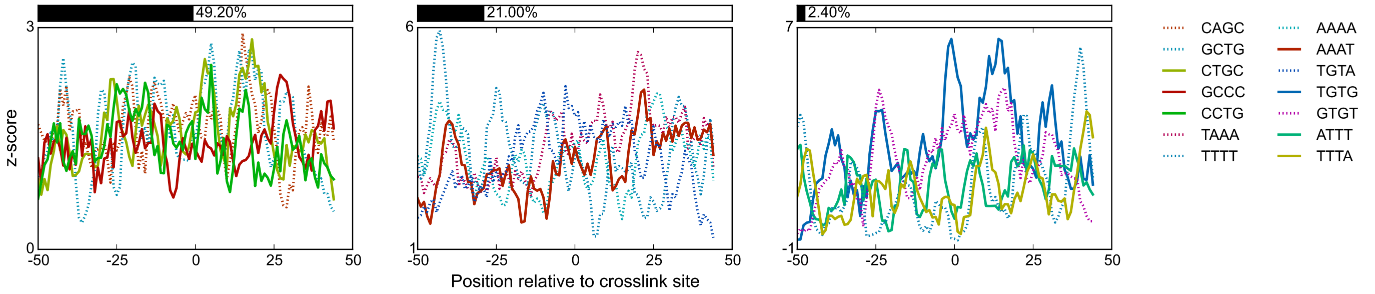

[4] Ago2 (2) ($\alpha = 10^2$)

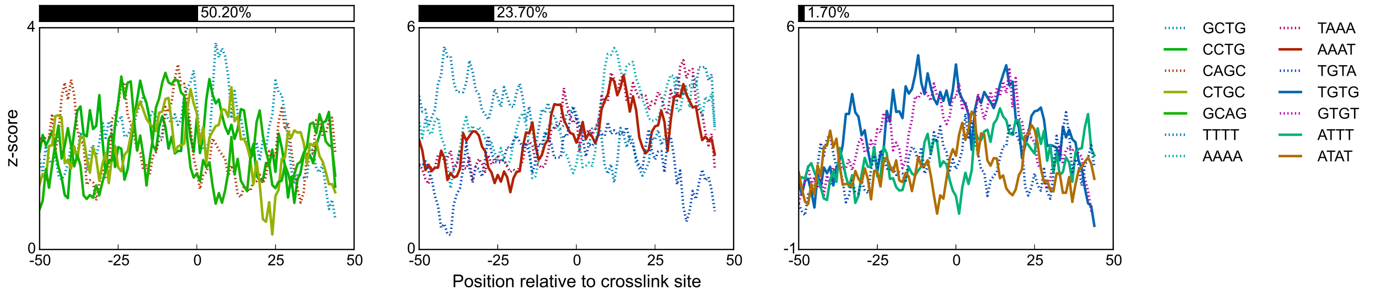

[5] Ago2 ($\alpha = 10^2$)

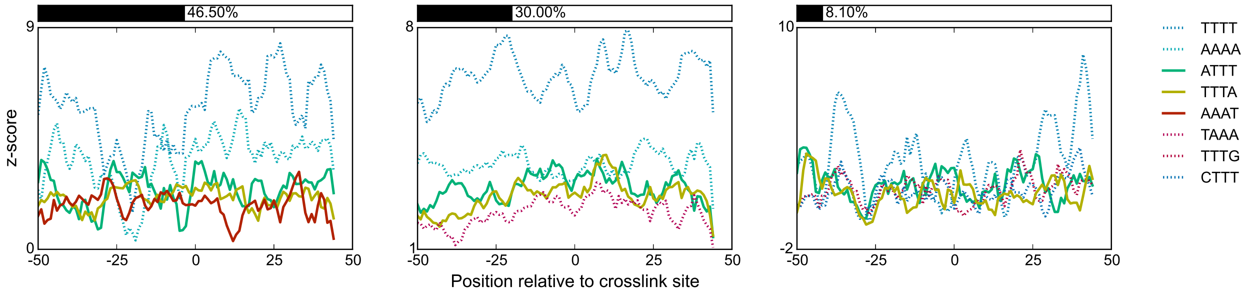

[6] eIF4AIII (1) ($\alpha = 10^3$)

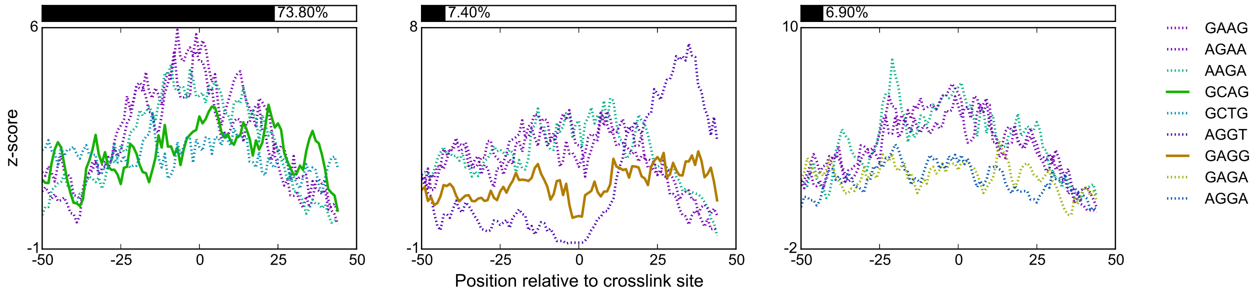

[7] eIF4AIII (2) ($\alpha = 10^2$)

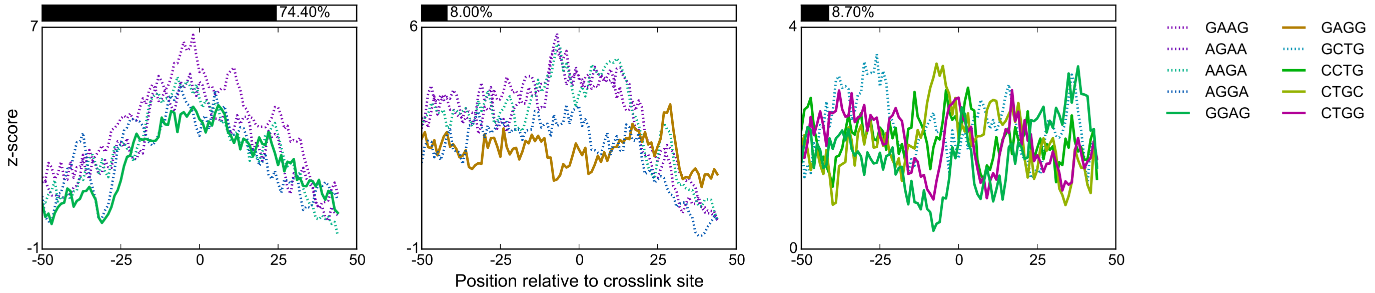

[8] ELAVL1 ($\alpha = 10^2$)

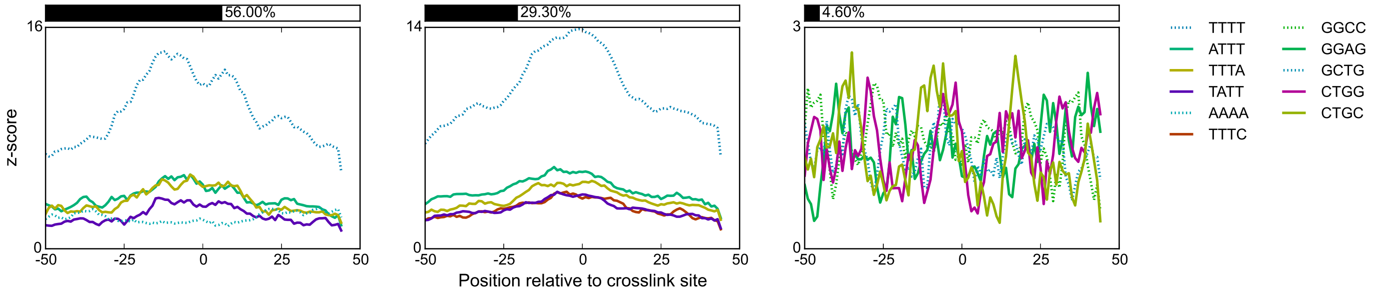

[9] ELAVL1-MNase ($\alpha = 10^0$)

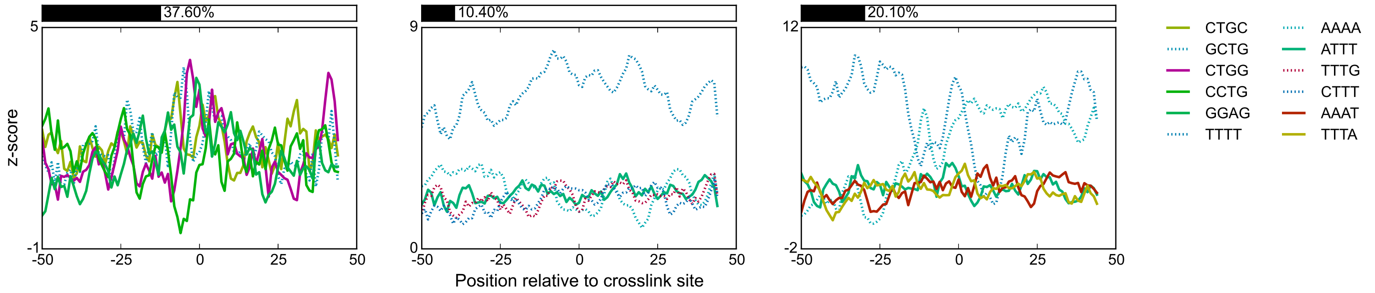

[10] ELAVL1A ($\alpha = 10^3$)

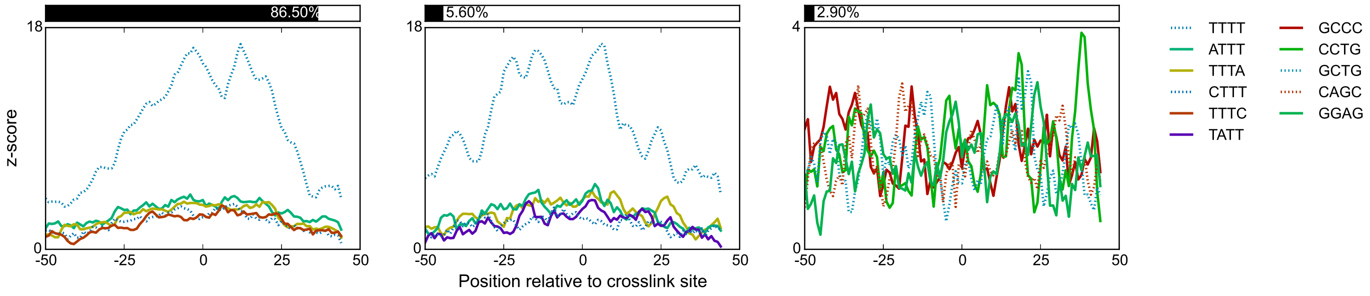

[11] ELAVL1 ($\alpha = 10^2$)

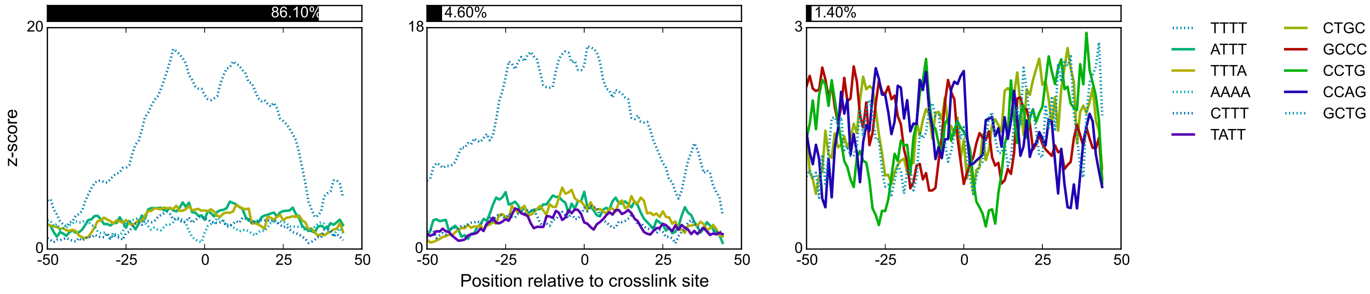

[12] ESWR1 ($\alpha = 10^2$)

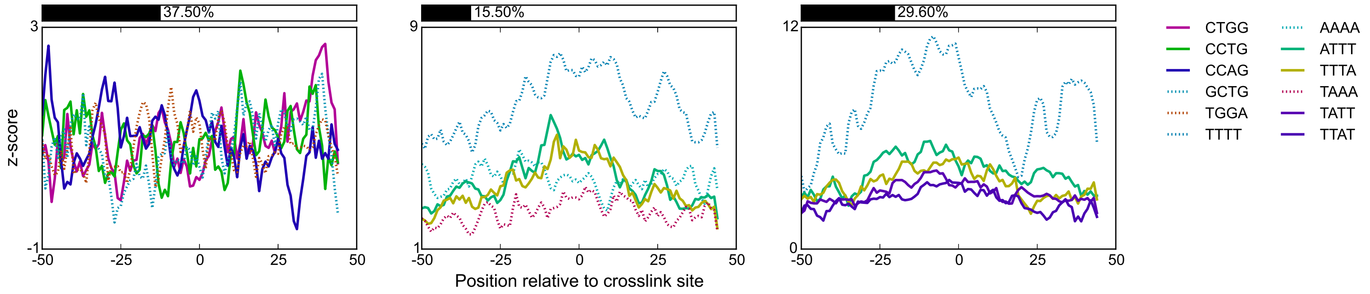

[13] FUS ($\alpha = 10^{-1}$)

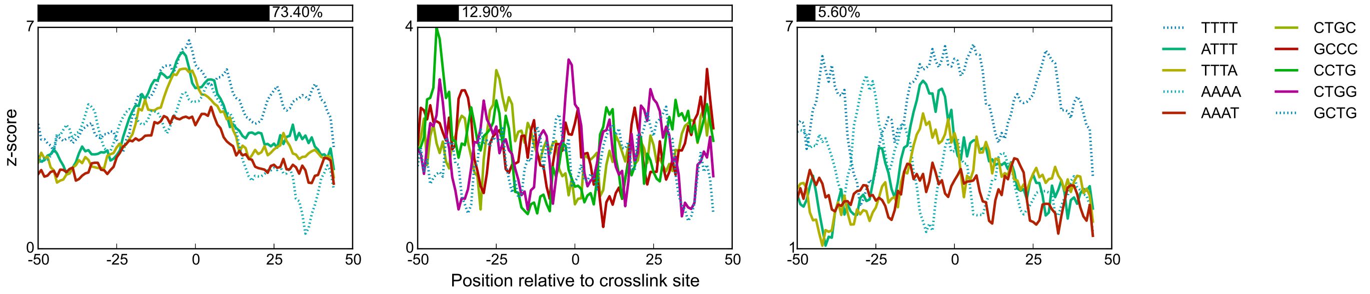

[14] Mut FUS ($\alpha = 10^2$)

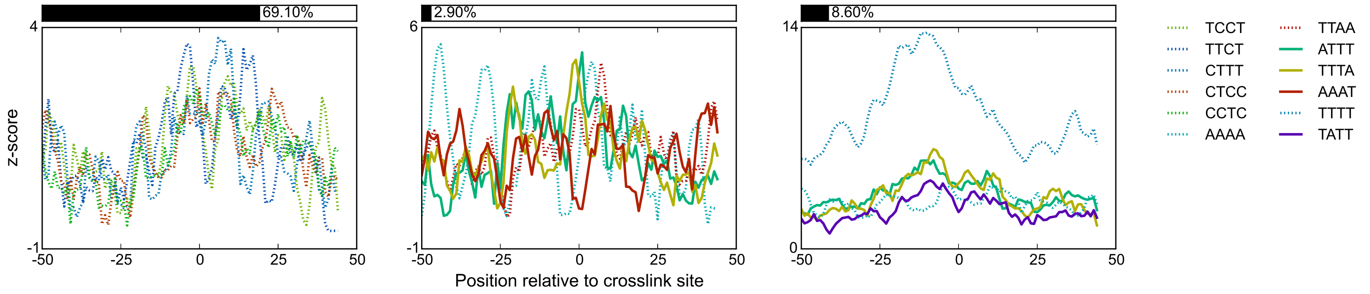

[15] IGF2BP1-3 ($\alpha = 10^2$)

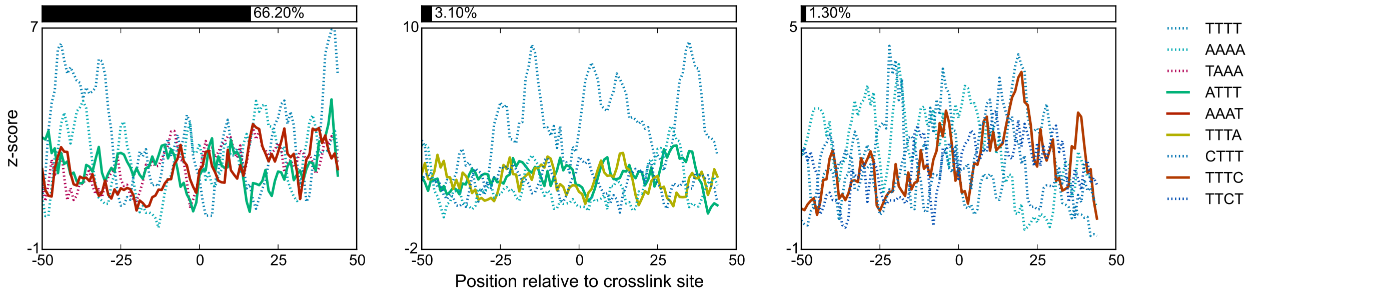

[16] hnRNPC ($\alpha = 10^0$)

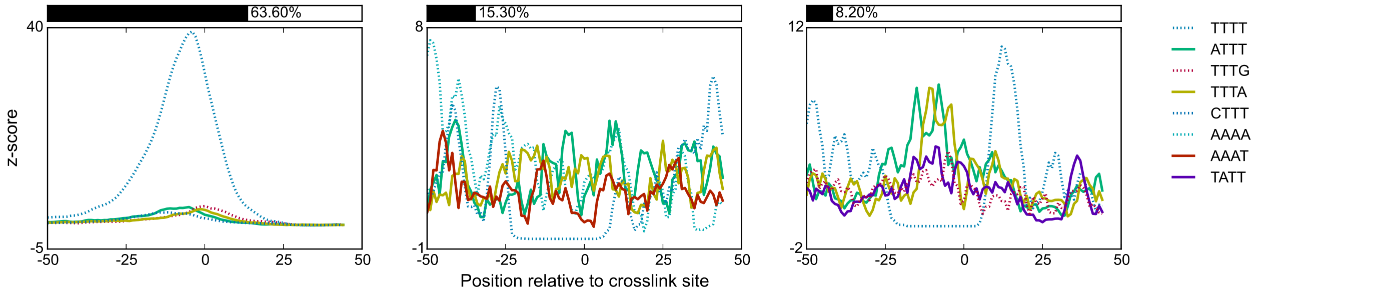

[17] hnRNPC ($\alpha = 10^0$)

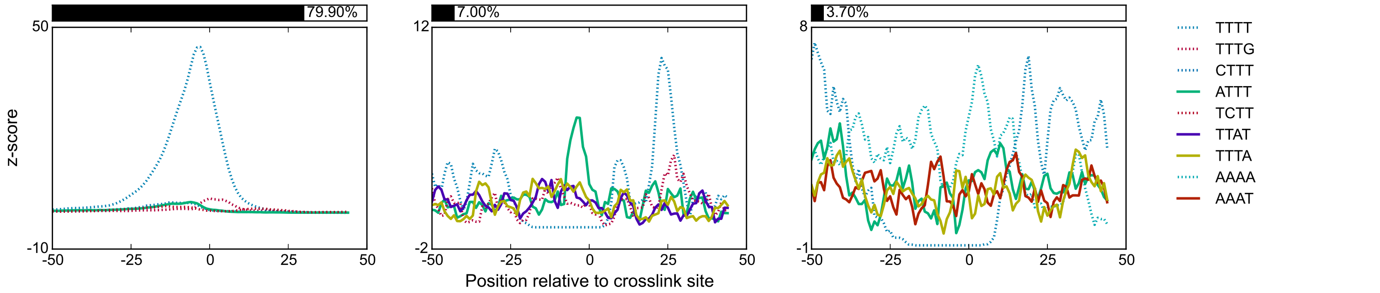

[18] hnRNPL ($\alpha = 10^3$)

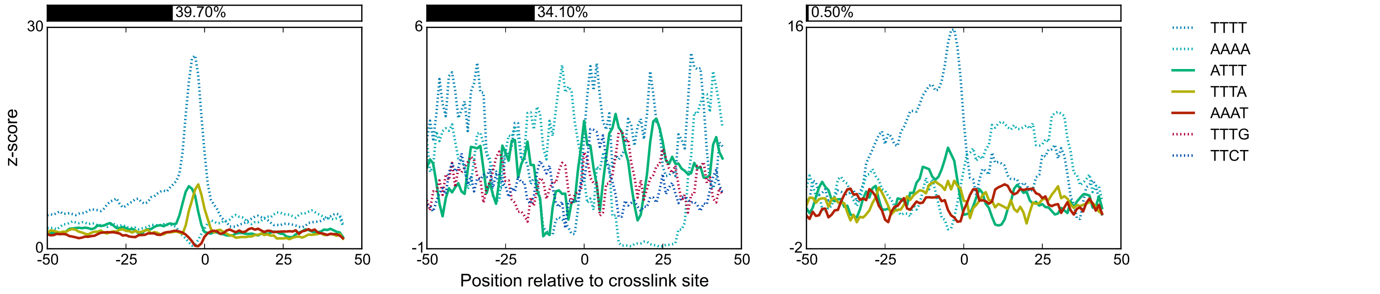

[19] hnRNPL ($\alpha = 10^5$)

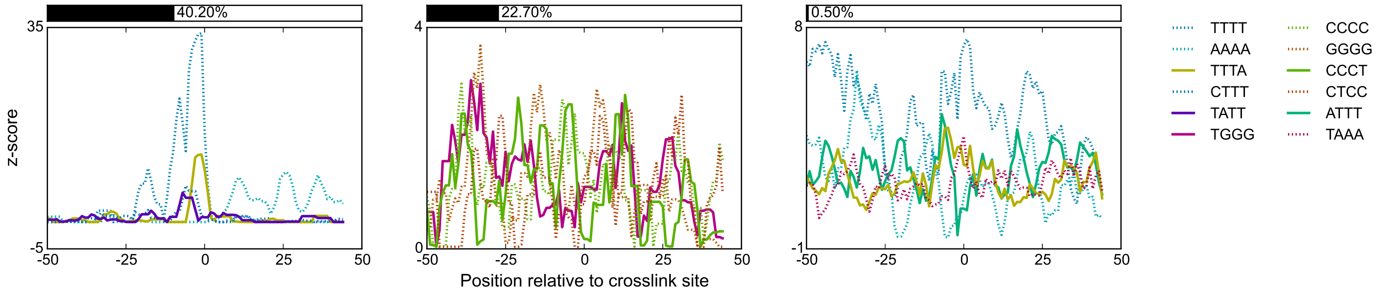

[20] hnRNPL-like ($\alpha = 10^3$)

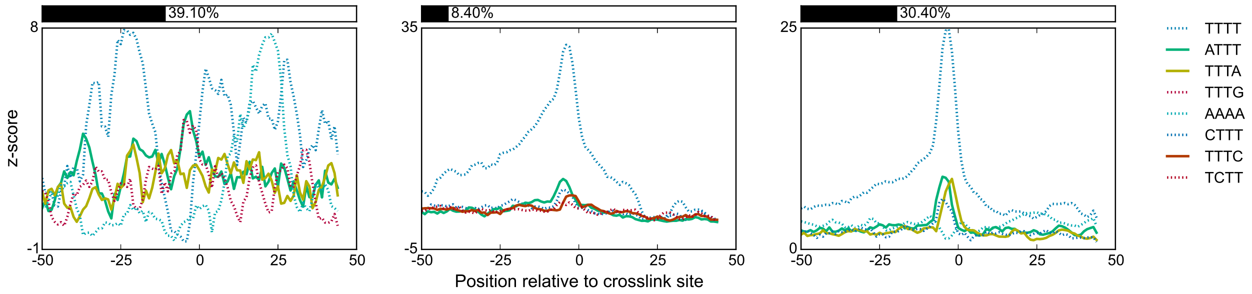

[21] MOV10 ($\alpha = 10^{-1}$)

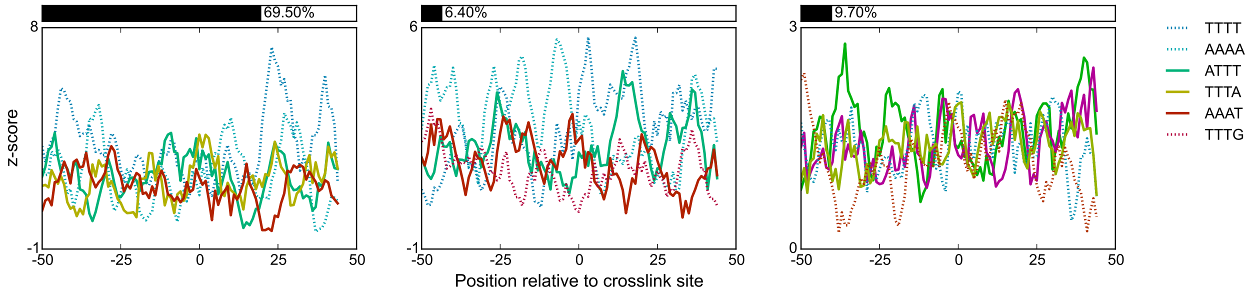

[22] Nsun2 ($\alpha = 10^5$)

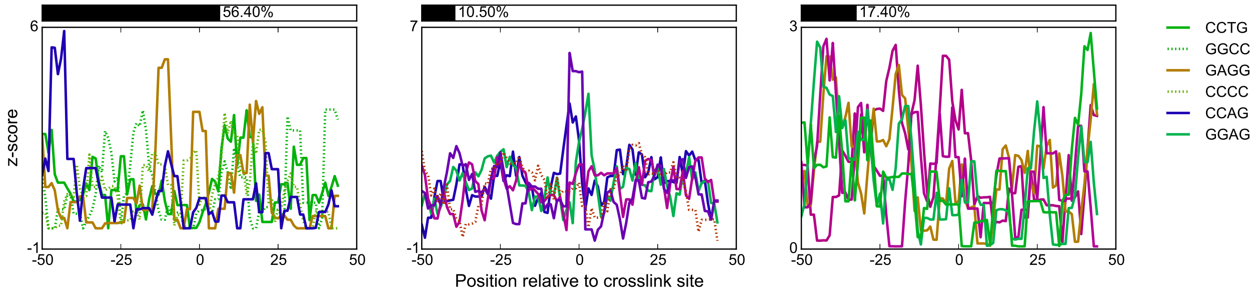

[23] PUM2 ($\alpha = 10^2$)

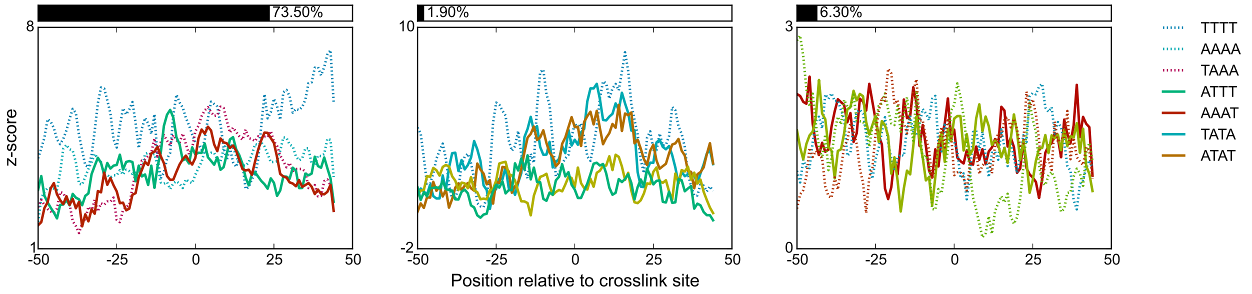

[24] QKI ($\alpha = 10^2$)

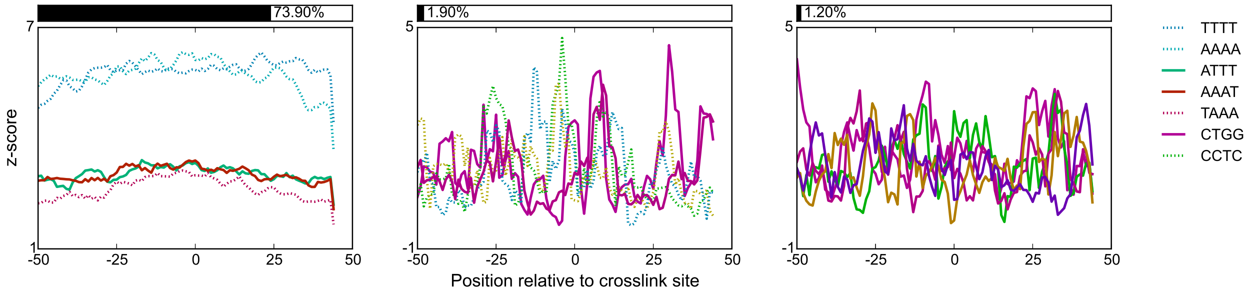

[25] SRSF1 ($\alpha = 10^2$)

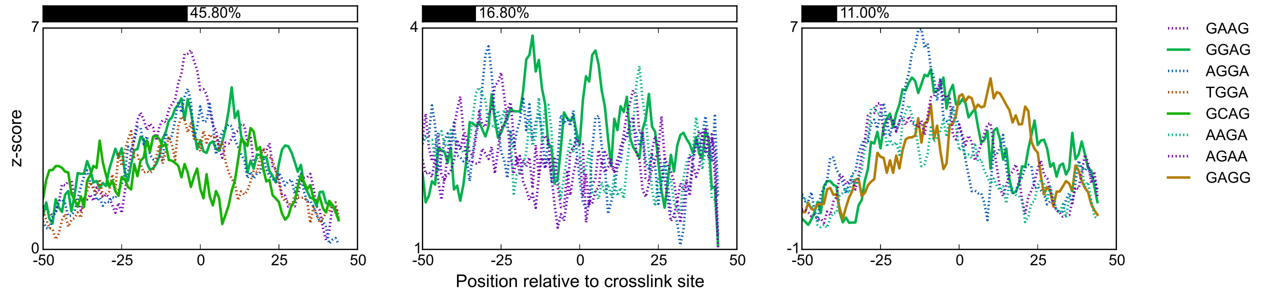

[26] TAF15 ($\alpha = 10^2$)

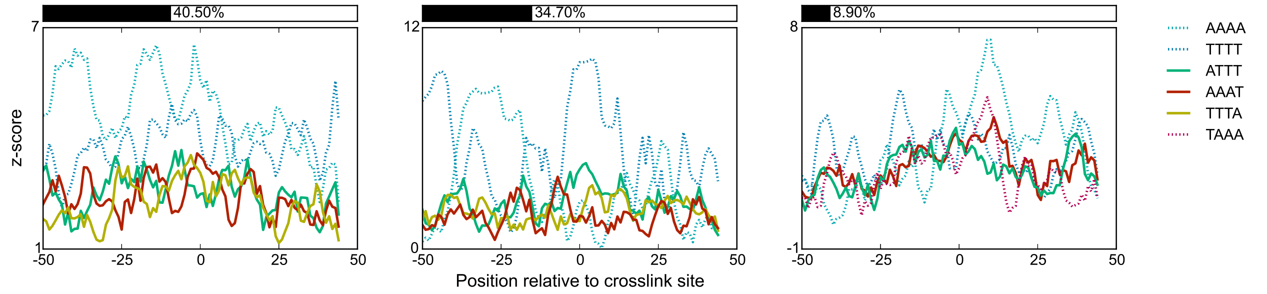

[27] TDP-43 ($\alpha = 10^1$)

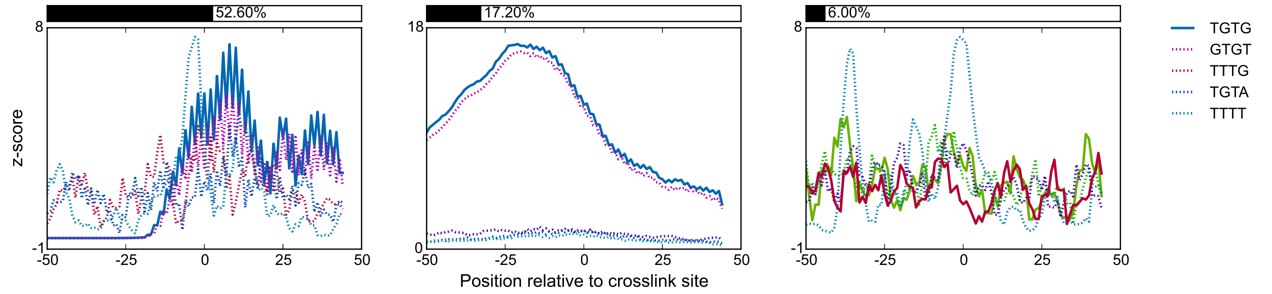

[28] TIA1 ($\alpha = 10^3$)

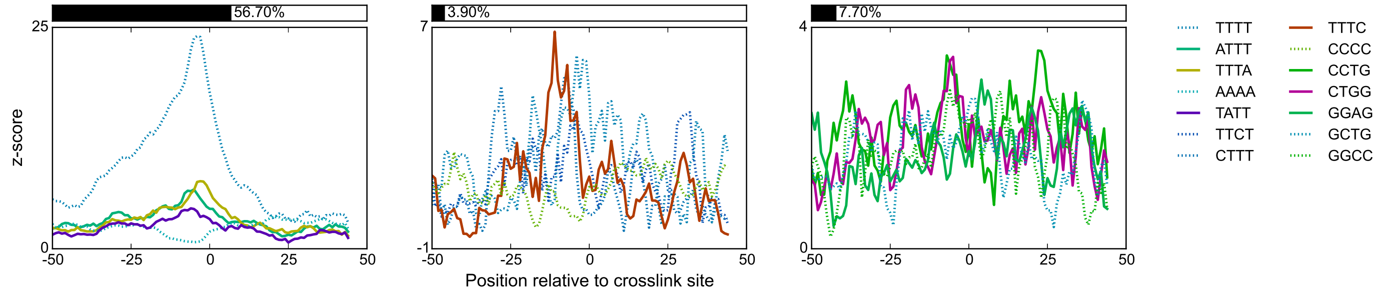

[29] TIAL1 ($\alpha = 10^3$)

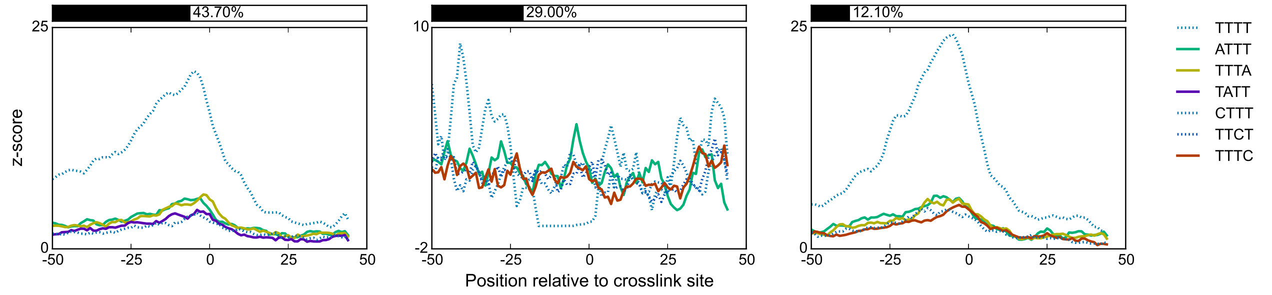

[30] U2AF2 ($\alpha = 10^2$)

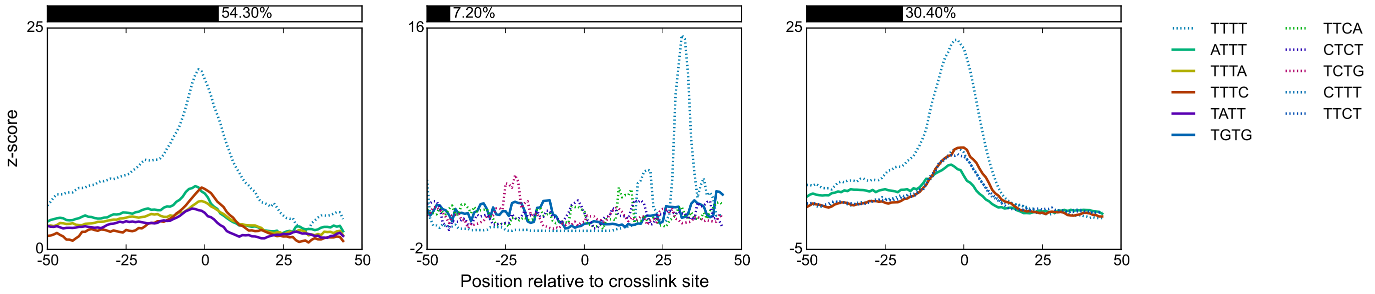

[31] U2AF2 (KD) ($\alpha = 10^{-1}$)

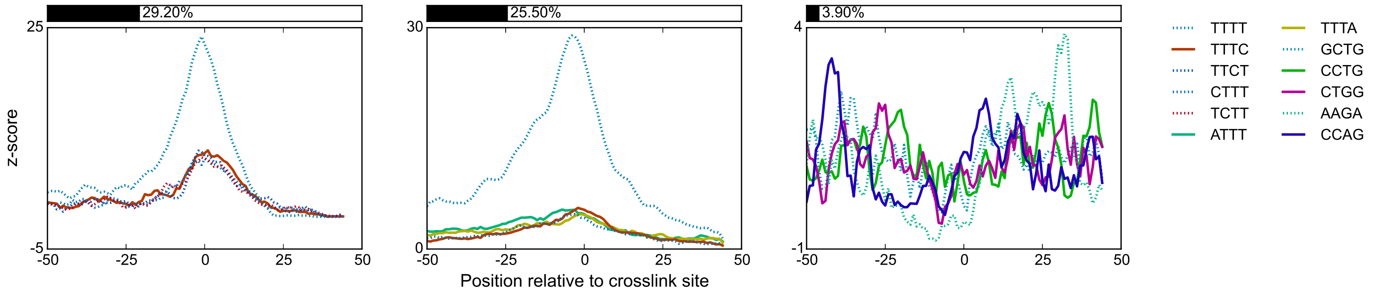

8.2 Region Type ($\mathbf{X_{RG}}$)

[1] Ago/EIF2C1-4 ($\alpha = 10^4$)

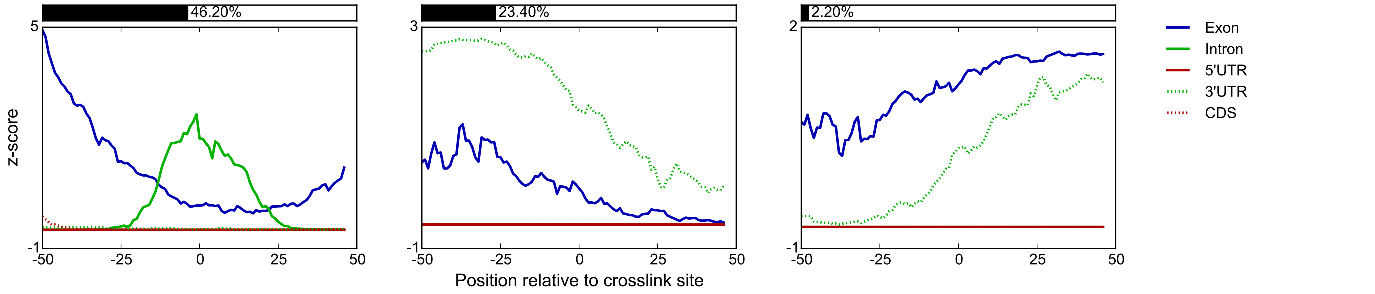

[2] Ago2-MNase ($\alpha = 10^3$)

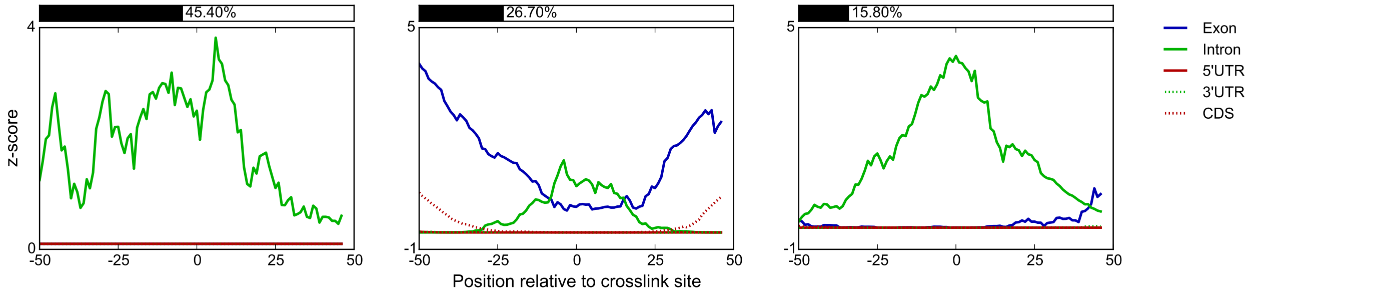

[3] Ago2 (1) ($\alpha = 10^{-1}$)

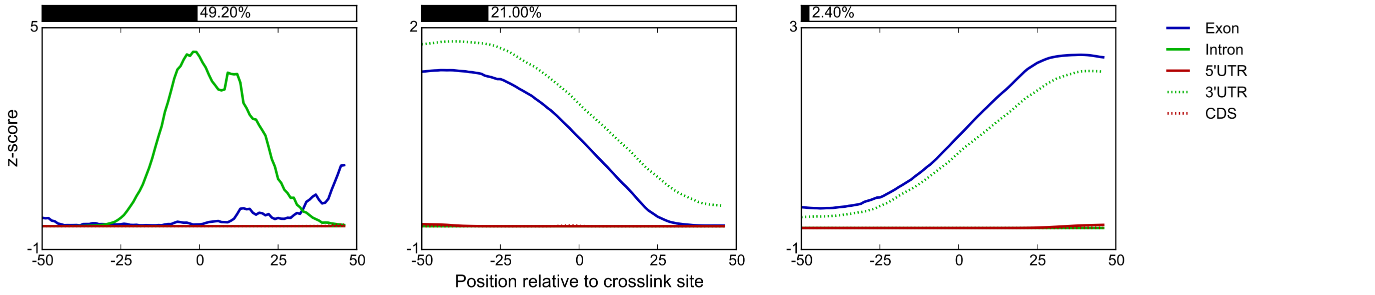

[4] Ago2 (2) ($\alpha = 10^2$)

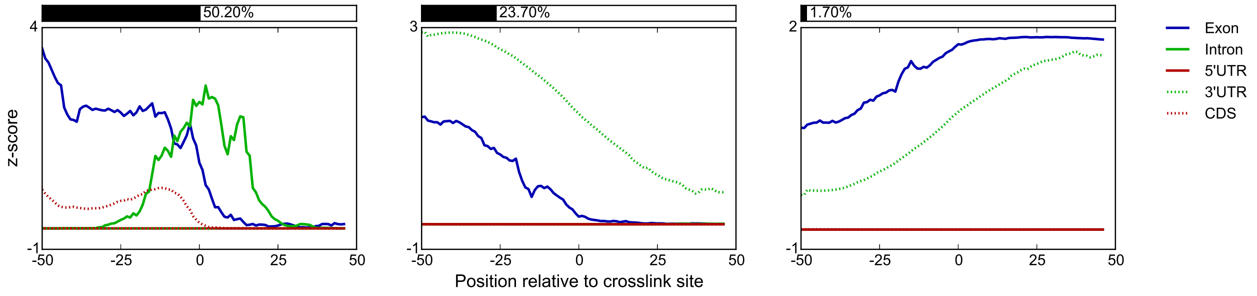

[5] Ago2 ($\alpha = 10^2$)

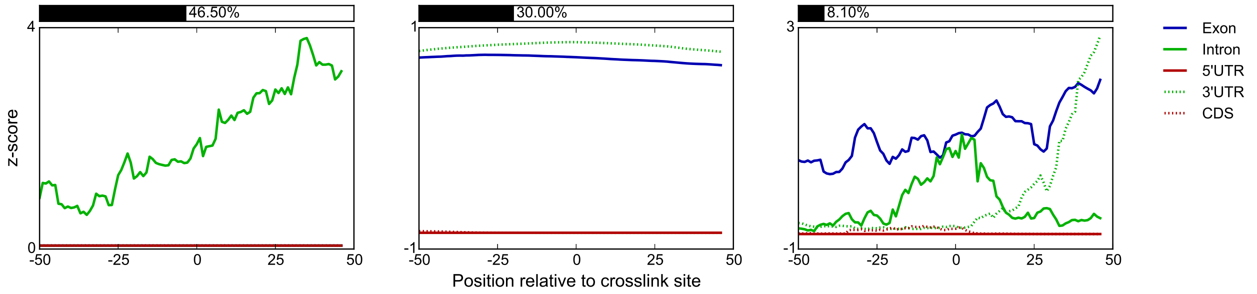

[6] eIF4AIII (1) ($\alpha = 10^3$)

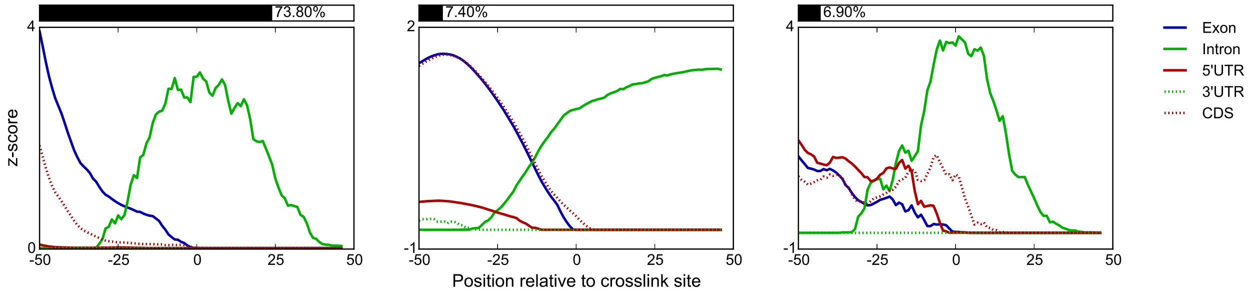

[7] eIF4AIII (2) ($\alpha = 10^2$)

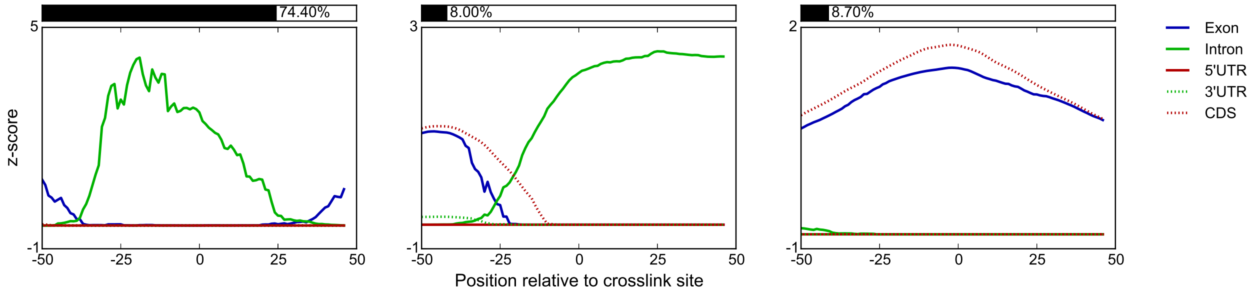

[8] ELAVL1 ($\alpha = 10^2$)

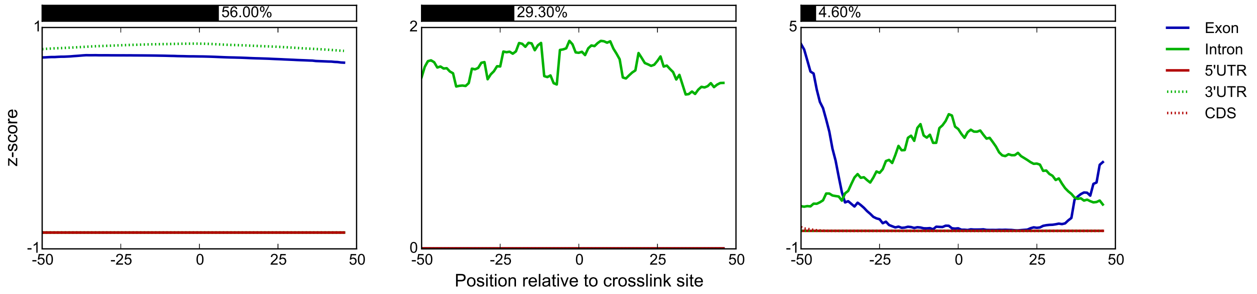

[9] ELAVL1-MNase ($\alpha = 10^0$)

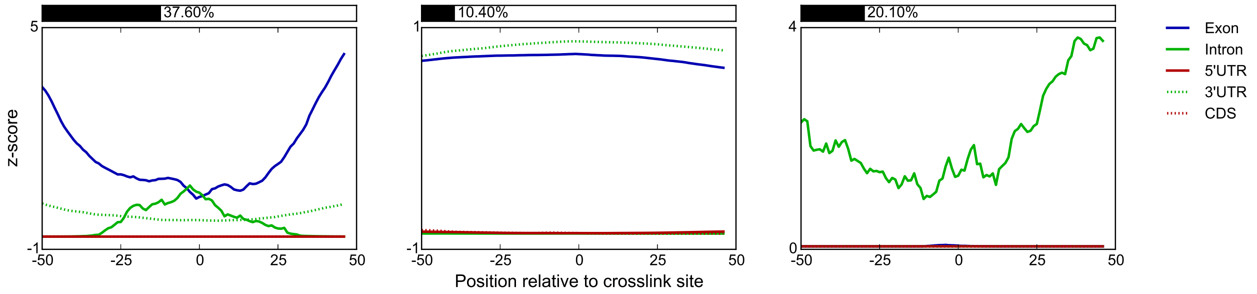

[10] ELAVL1A ($\alpha = 10^3$)

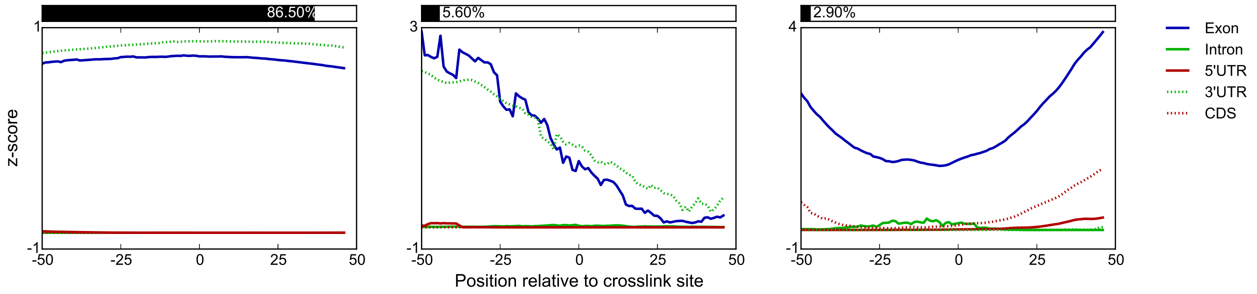

[11] ELAVL1 ($\alpha = 10^2$)

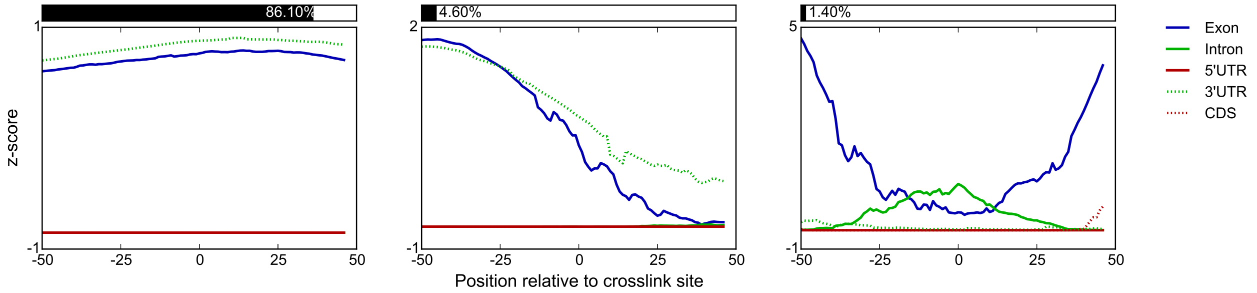

[12] ESWR1 ($\alpha = 10^2$)

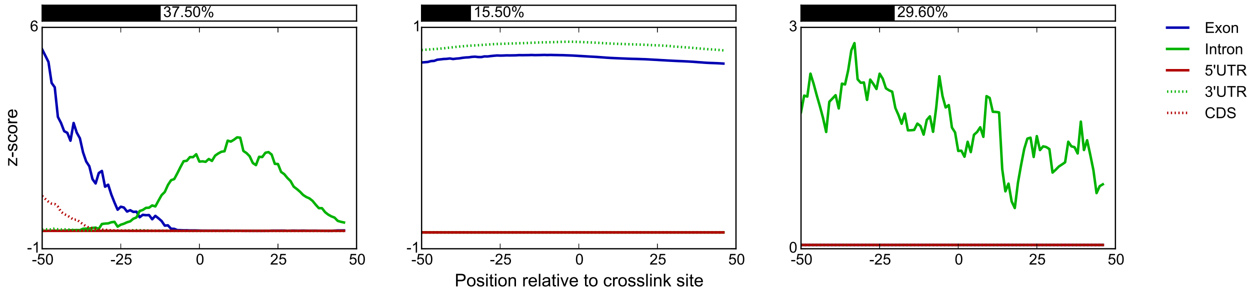

[13] FUS ($\alpha = 10^{-1}$)

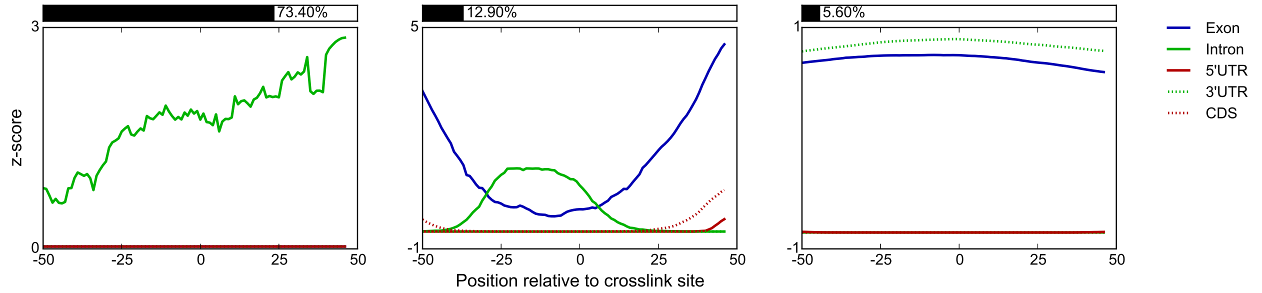

[14] Mut FUS ($\alpha = 10^2$)

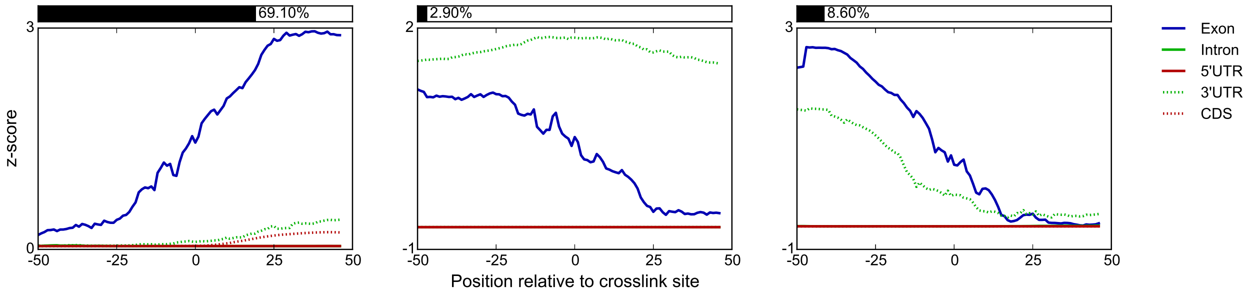

[15] IGF2BP1-3 ($\alpha = 10^2$)

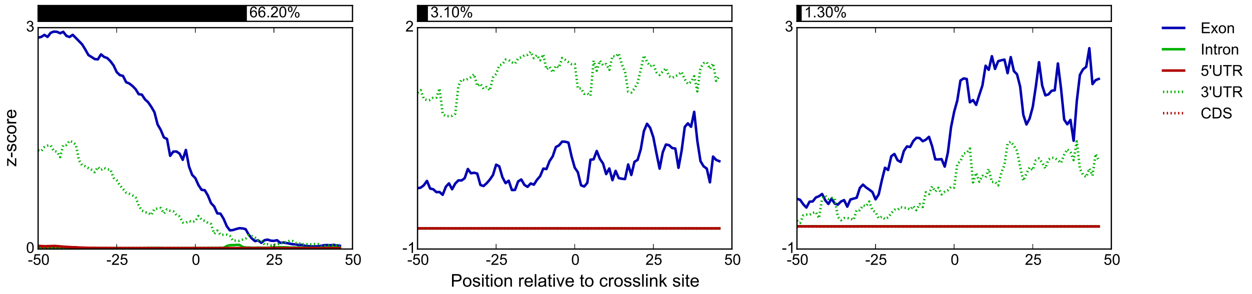

[16] hnRNPC ($\alpha = 10^0$)

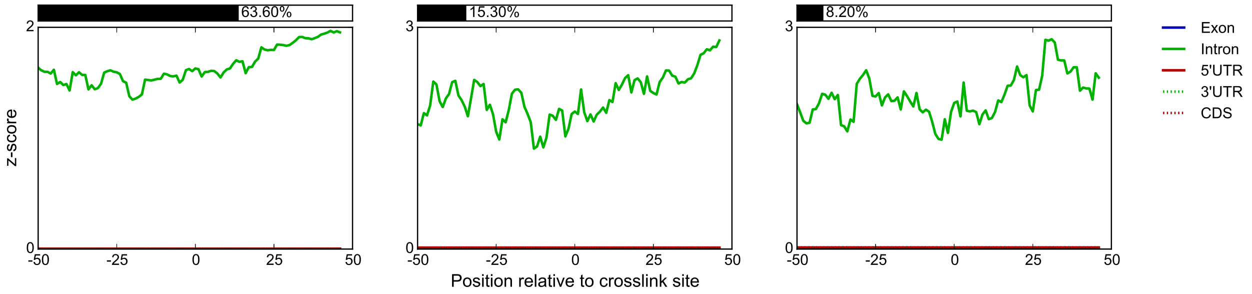

[17] hnRNPC ($\alpha = 10^0$)

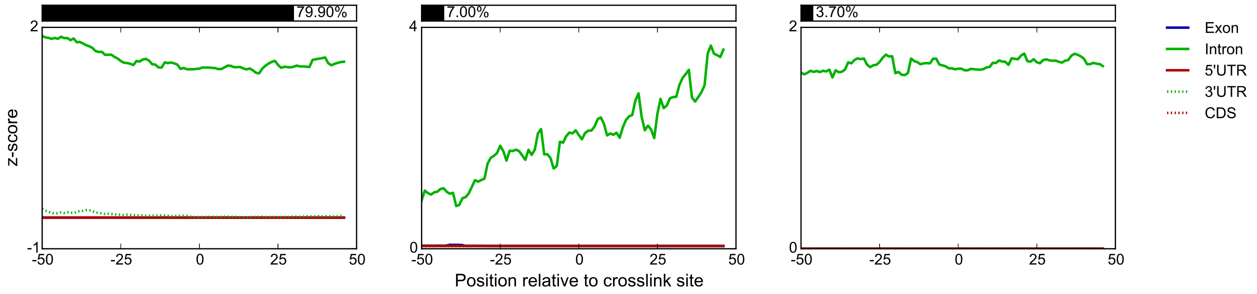

[18] hnRNPL ($\alpha = 10^3$)

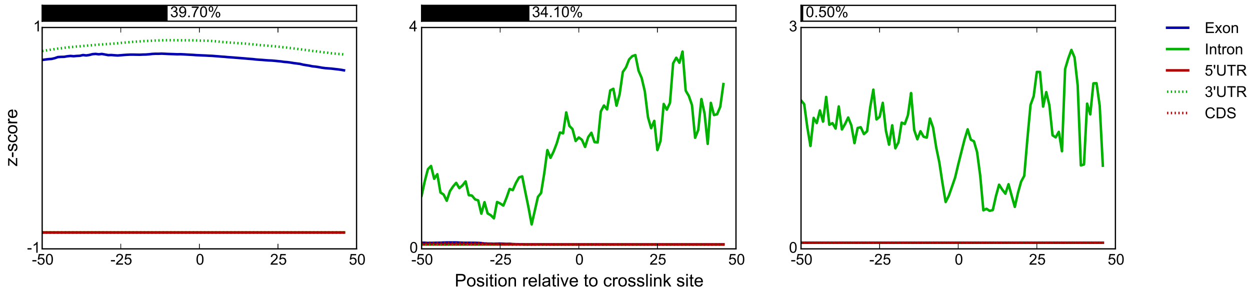

[19] hnRNPL ($\alpha = 10^5$)

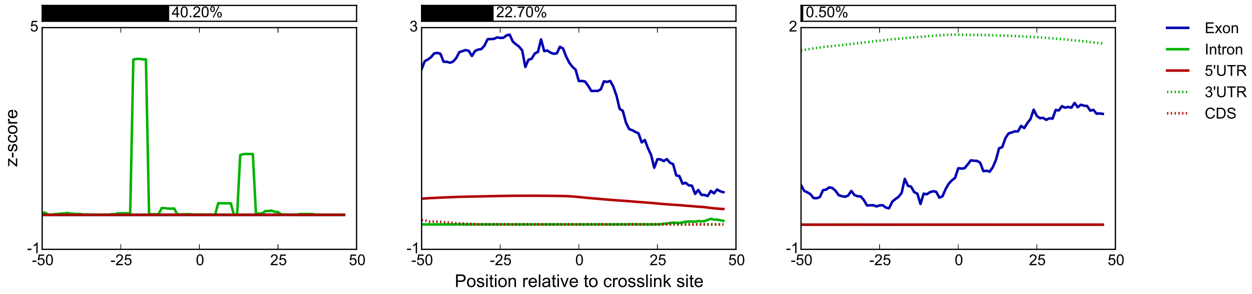

[20] hnRNPL-like ($\alpha = 10^3$)

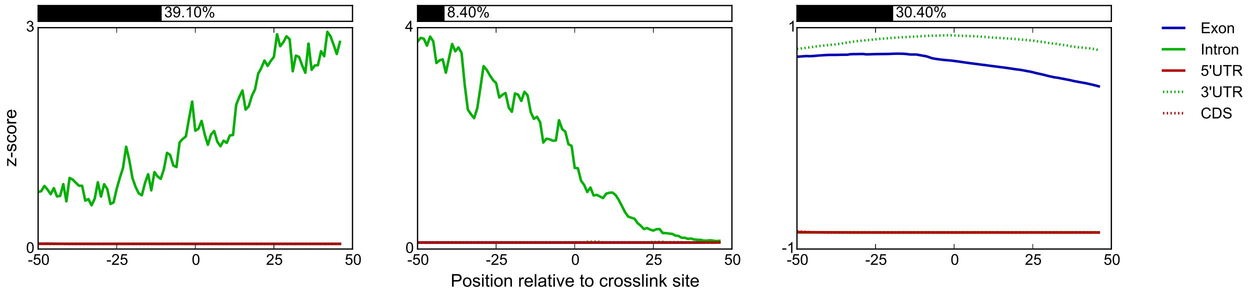

[21] MOV10 ($\alpha = 10^{-1}$)

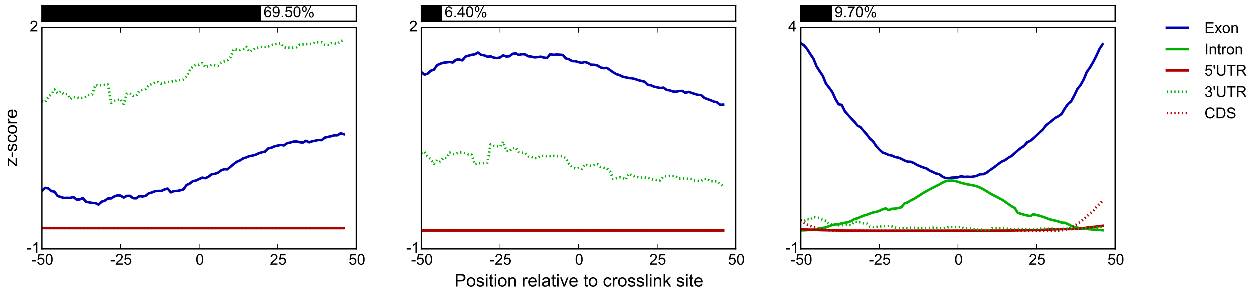

[22] Nsun2 ($\alpha = 10^5$)

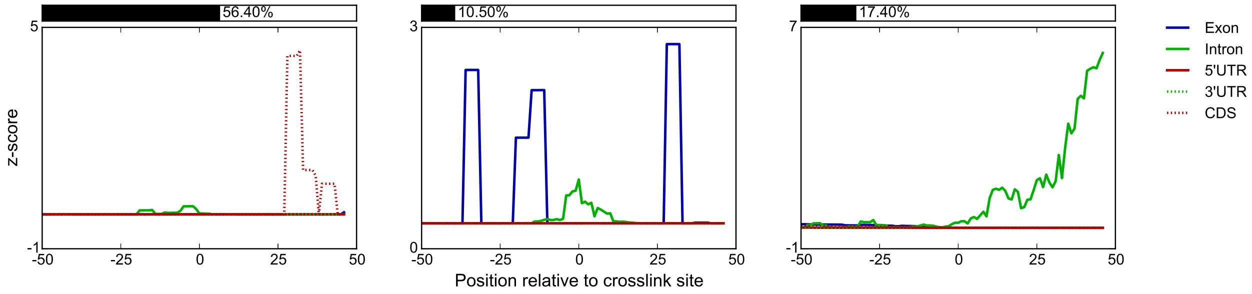

[23] PUM2 ($\alpha = 10^2$)

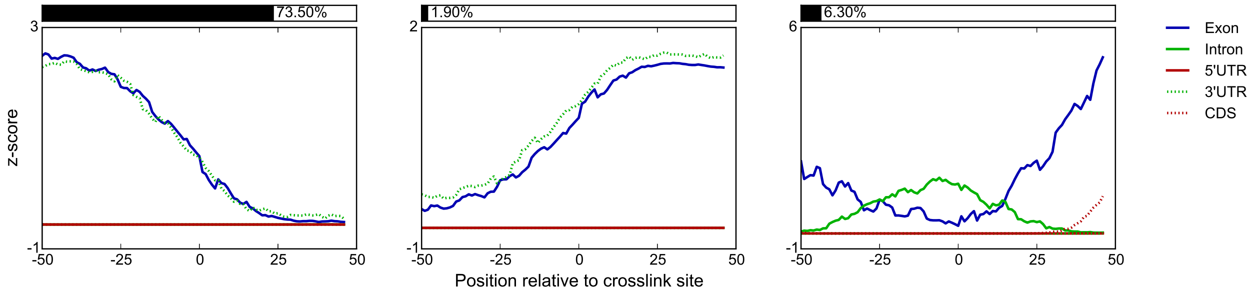

[24] QKI ($\alpha = 10^2$)

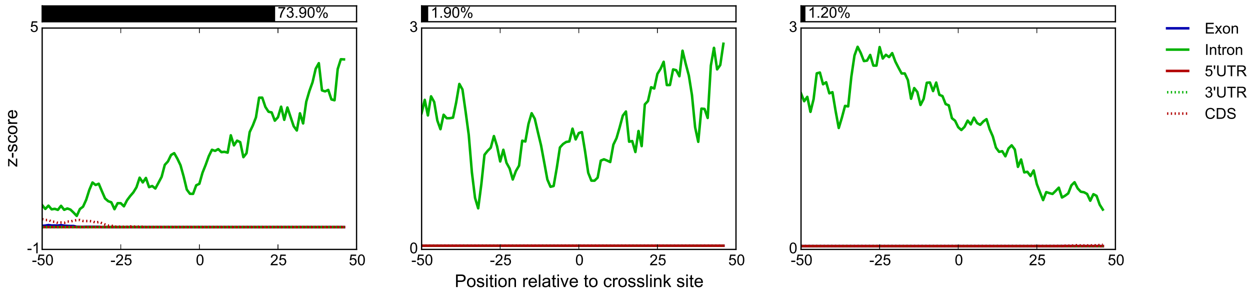

[25] SRSF1 ($\alpha = 10^2$)

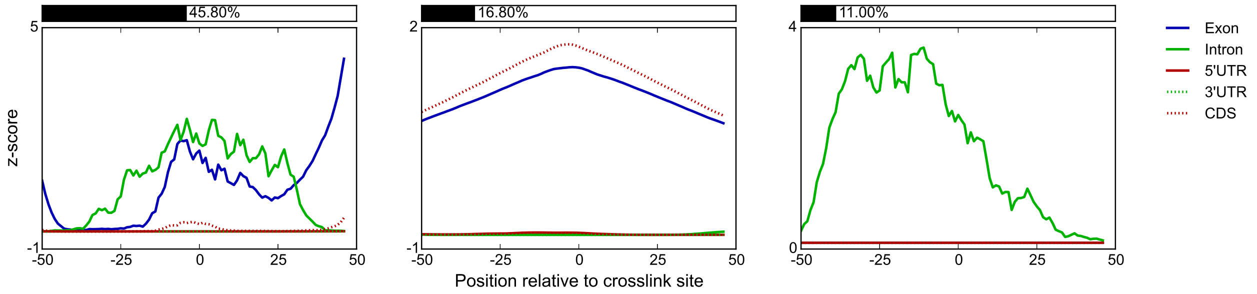

[26] TAF15 ($\alpha = 10^2$)

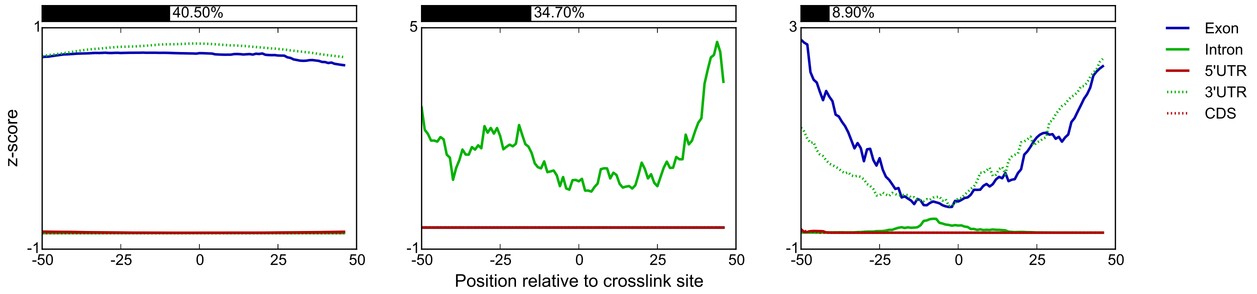

[27] TDP-43 ($\alpha = 10^1$)

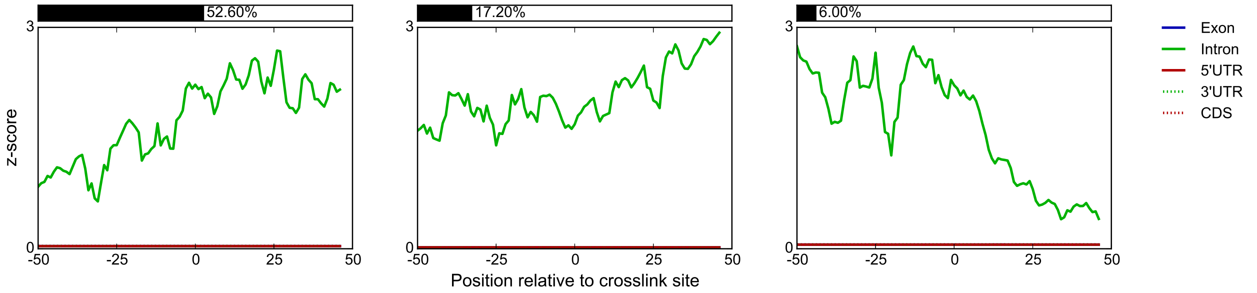

[28] TIA1 ($\alpha = 10^3$)

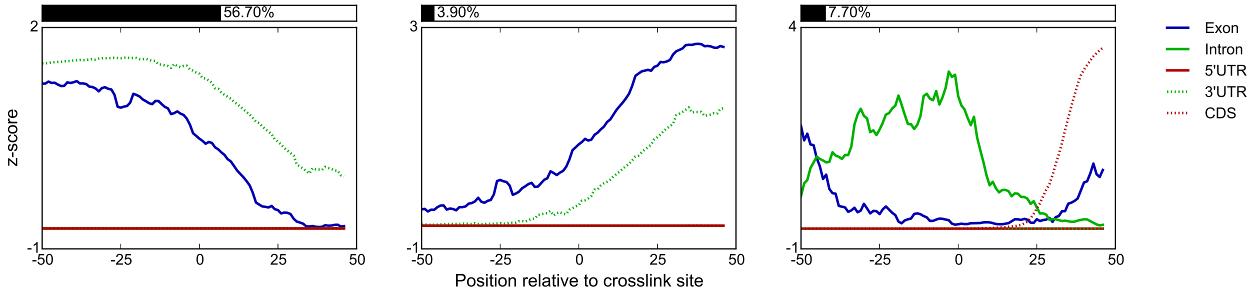

[29] TIAL1 ($\alpha = 10^3$)

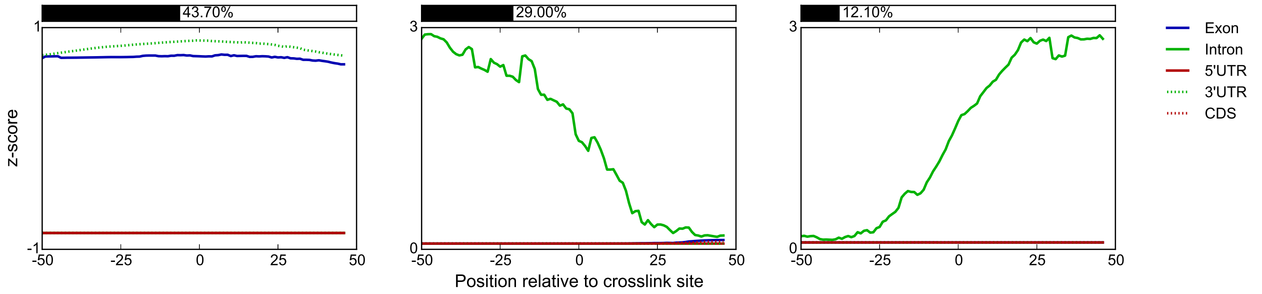

[30] U2AF2 ($\alpha = 10^2$)

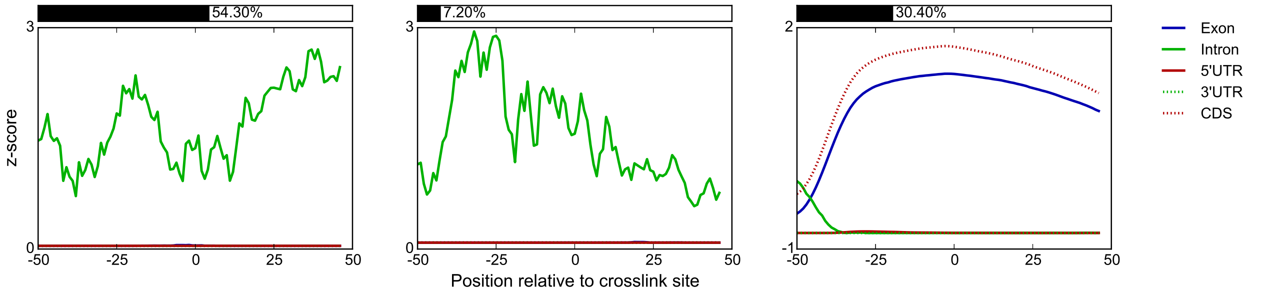

[31] U2AF2 (KD) ($\alpha = 10^{-1}$)

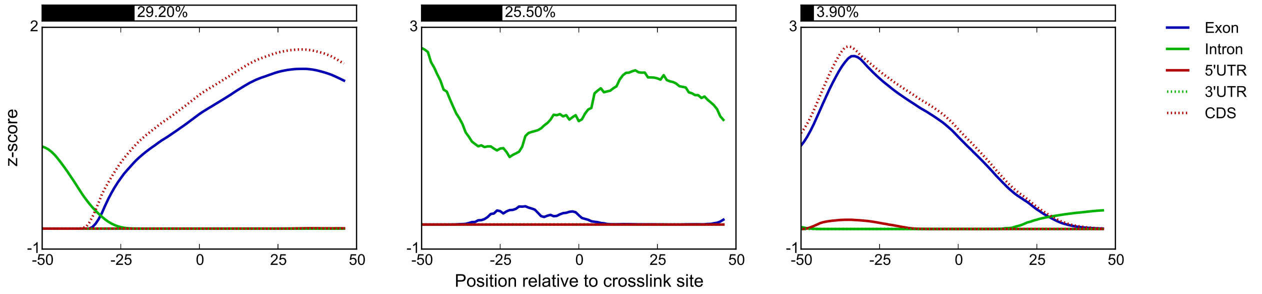

8.3 RNAfold (X_{RNA})

[1] Ago/EIF2C1-4 ($\alpha = 10^4$)

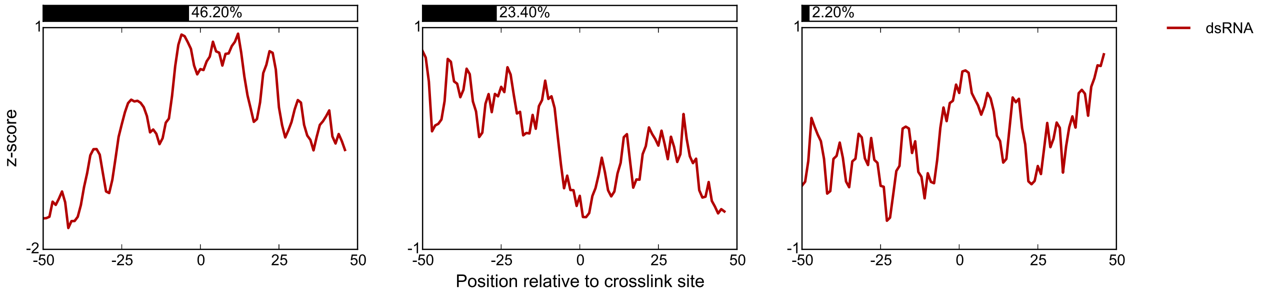

[2] Ago2-MNase ($\alpha = 10^3$)

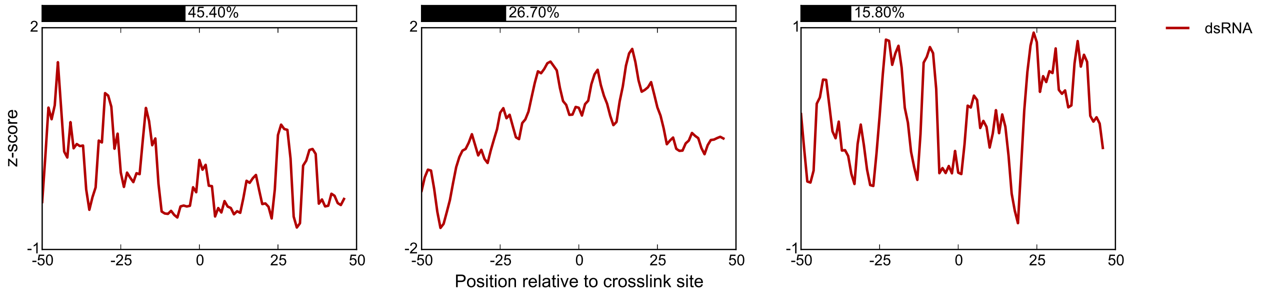

[3] Ago2 (1) ($\alpha = 10^{-1}$)

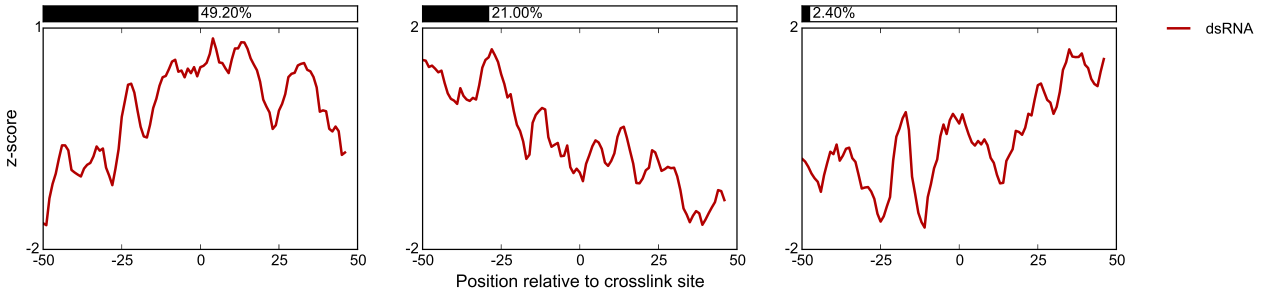

[4] Ago2 (2) ($\alpha = 10^{2}$)

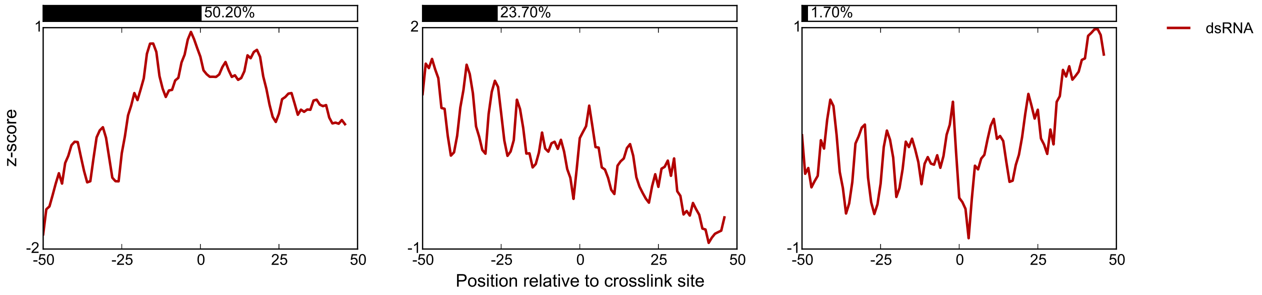

[5] Ago2 ($\alpha = 10^{2}$)

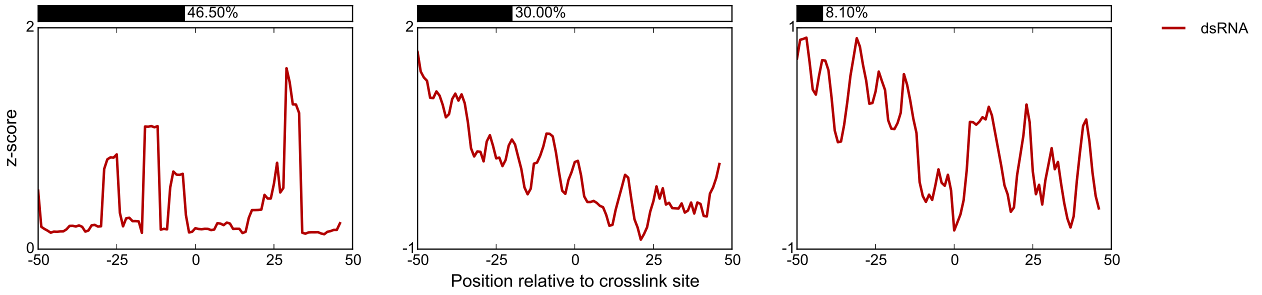

[6] eIF4AIII (1) ($\alpha = 10^{3}$)

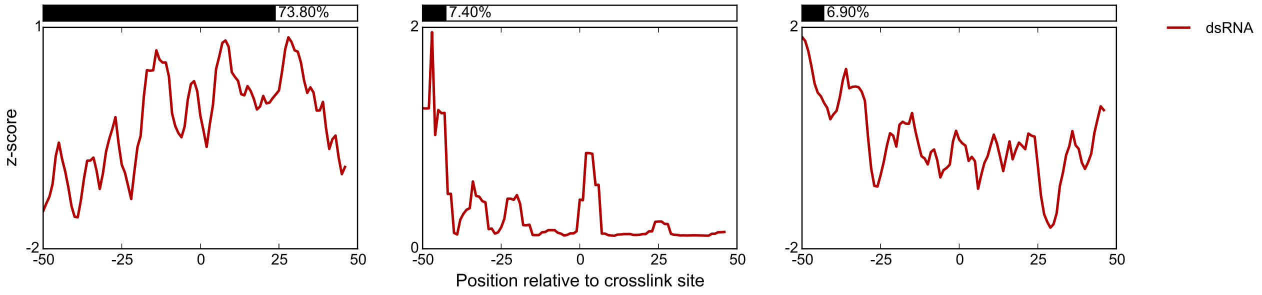

[7] eIF4AIII (2) ($\alpha = 10^{2}$)

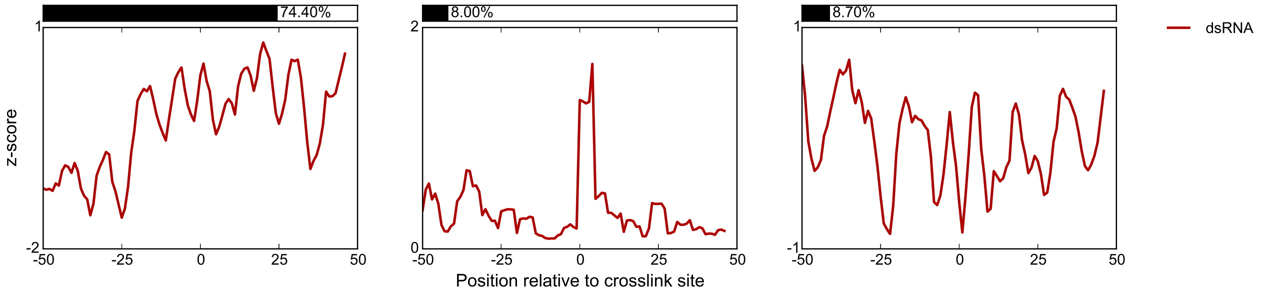

[8] ELAVL1 ($\alpha = 10^2$)

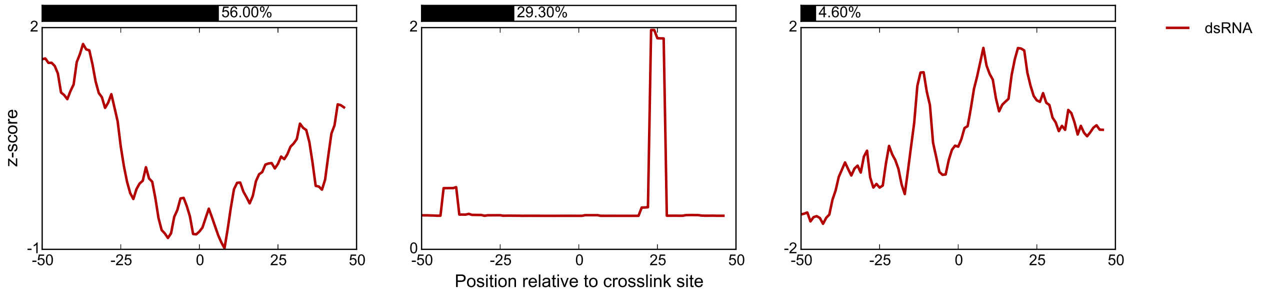

[9] ELAVL1-MNase ($\alpha = 10^0$)

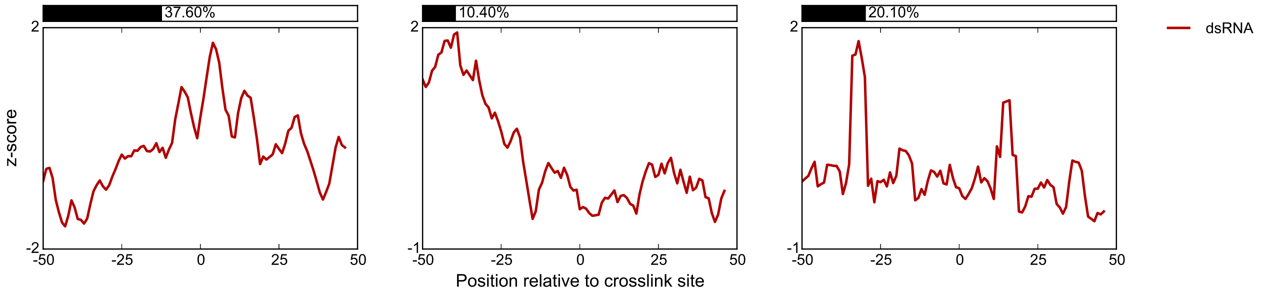

[10] ELAVL1A ($\alpha = 10^3$)

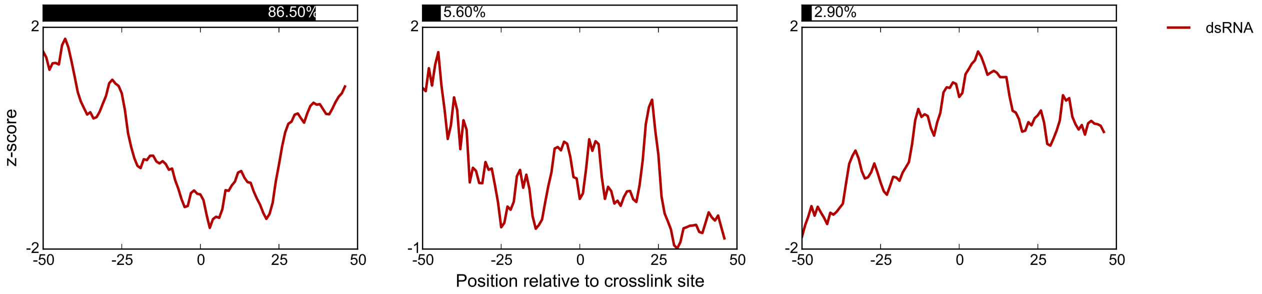

[11] ELAVL1 ($\alpha = 10^2$)

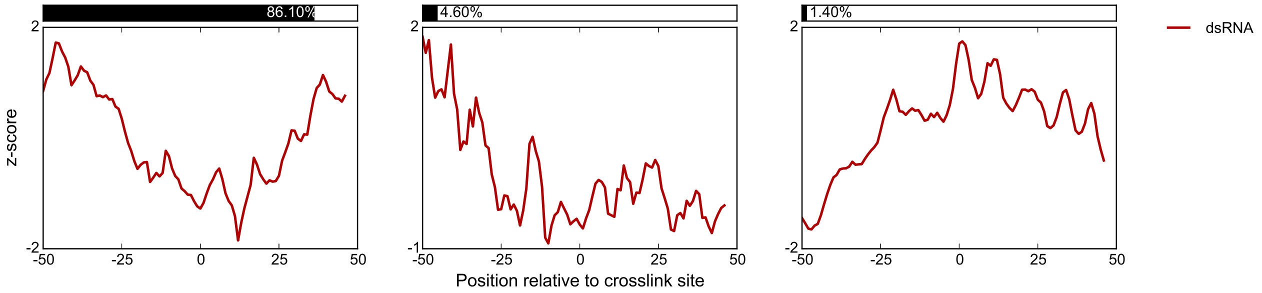

[12] ESWR1 ($\alpha = 10^2$)

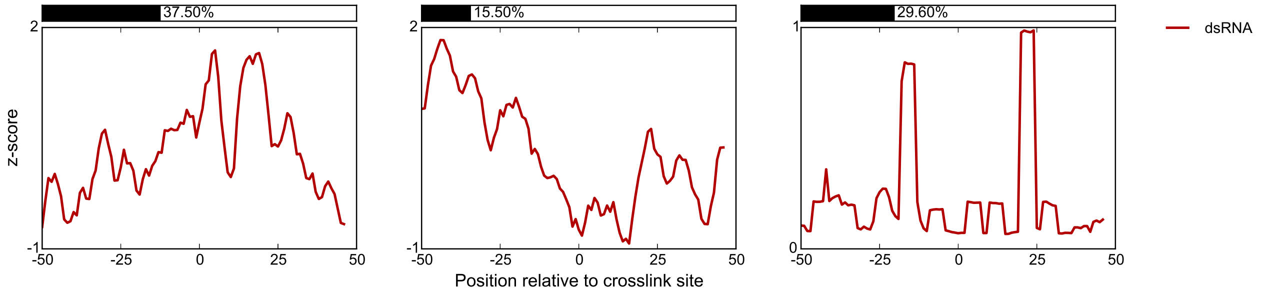

[13] FUS ($\alpha = 10^{-1}$)

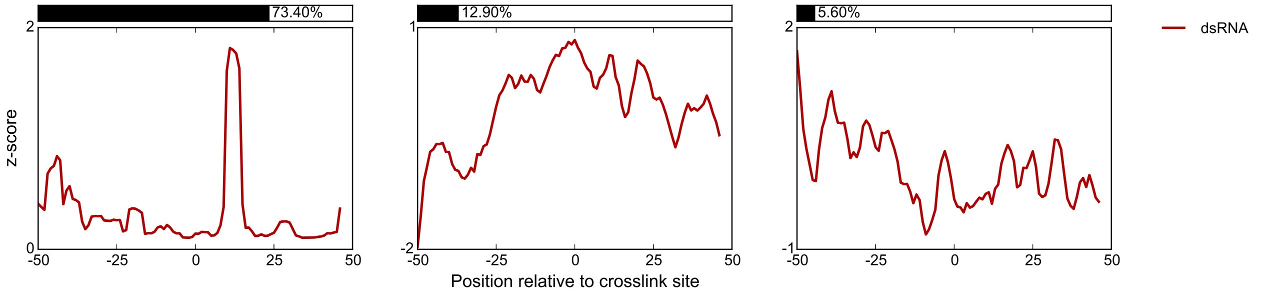

[14] Mut FUS ($\alpha = 10^{2}$)

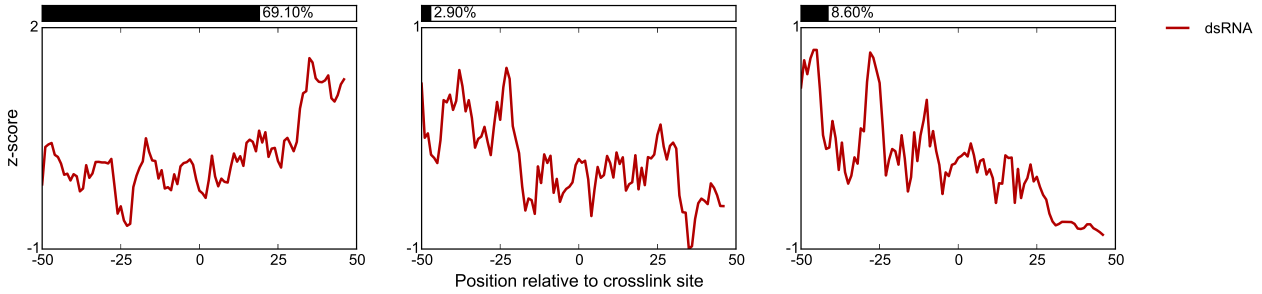

[15] IGF2BP1-3 ($\alpha = 10^{2}$)

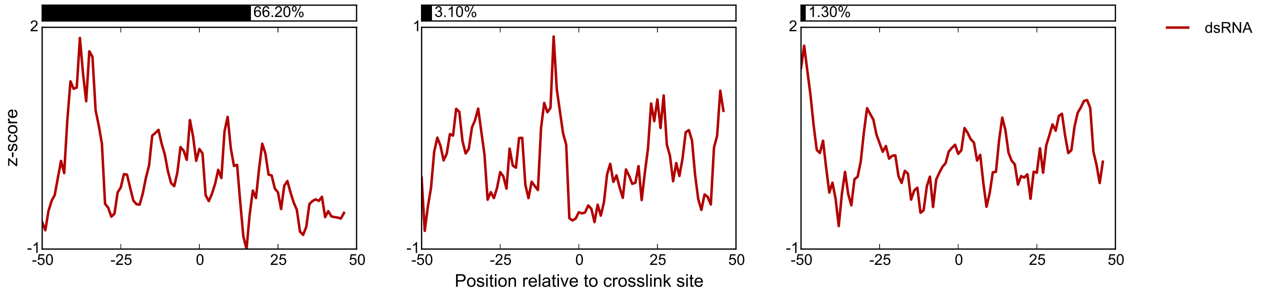

[16] hnRNPC ($\alpha = 10^{0}$)

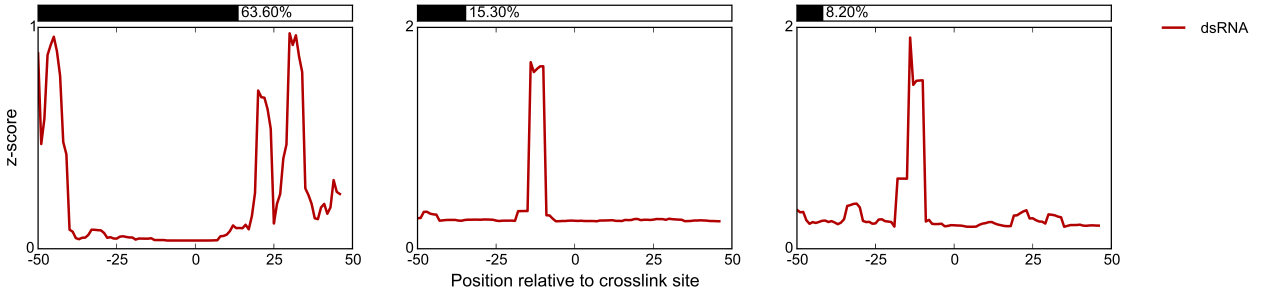

[17] hnRNPC ($\alpha = 10^{0}$)

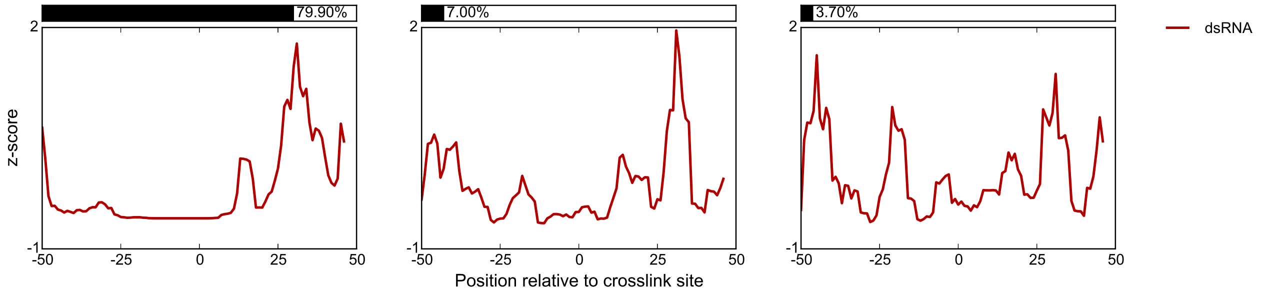

[18] hnRNPL ($\alpha = 10^3$)

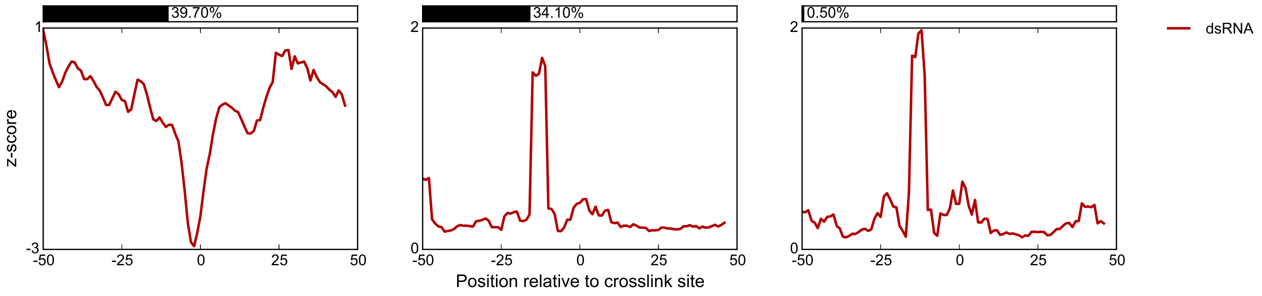

[19] hnRNPL ($\alpha = 10^5$)

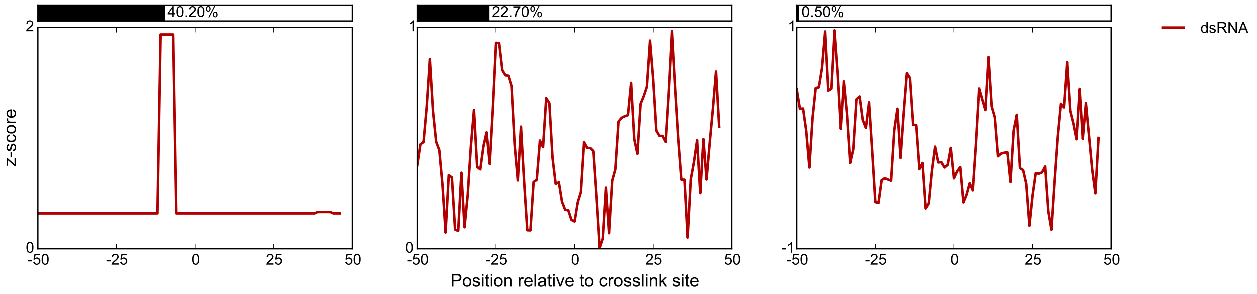

[20] hnRNPL-like ($\alpha = 10^3$)

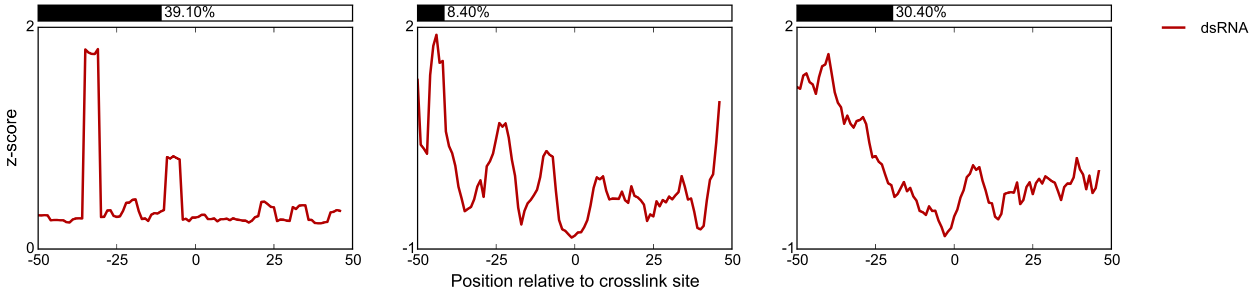

[21] MOV10 ($\alpha = 10^{-1}$)

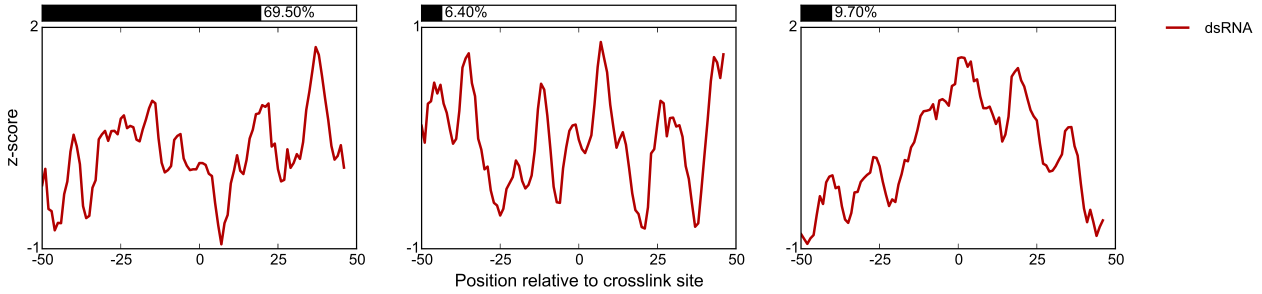

[22] Nsun2 ($\alpha = 10^5$)

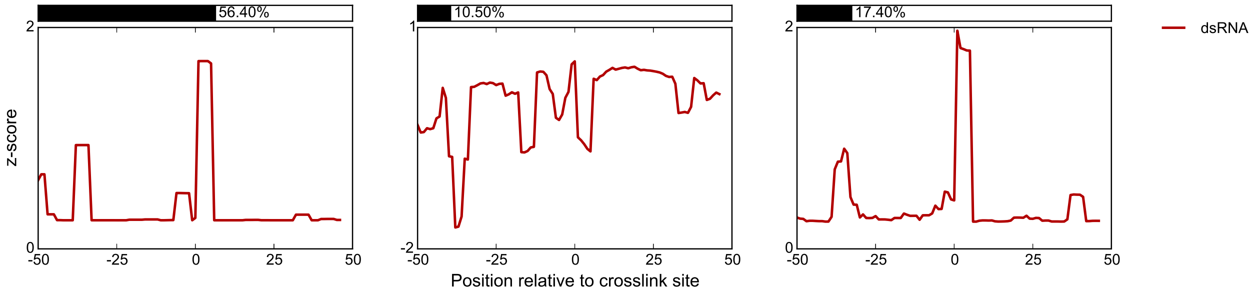

[23] PUM2 ($\alpha = 10^2$)

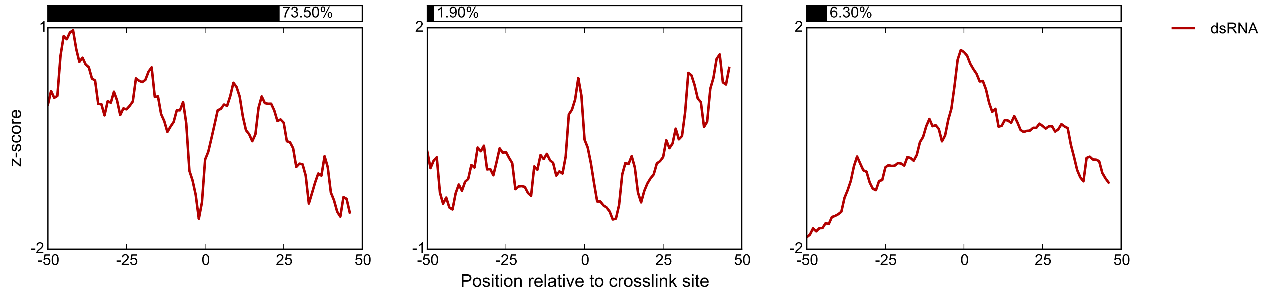

[24] QKI ($\alpha = 10^2$)

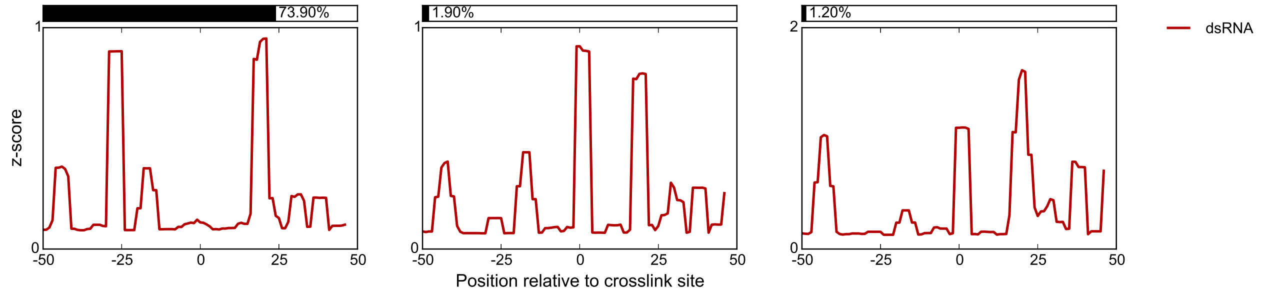

[25] SRSF1 ($\alpha = 10^2$)

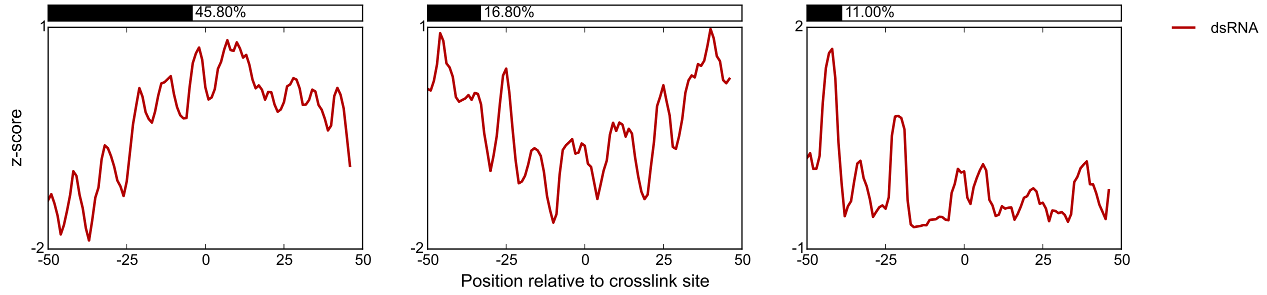

[26] TAF15 ($\alpha = 10^2$)

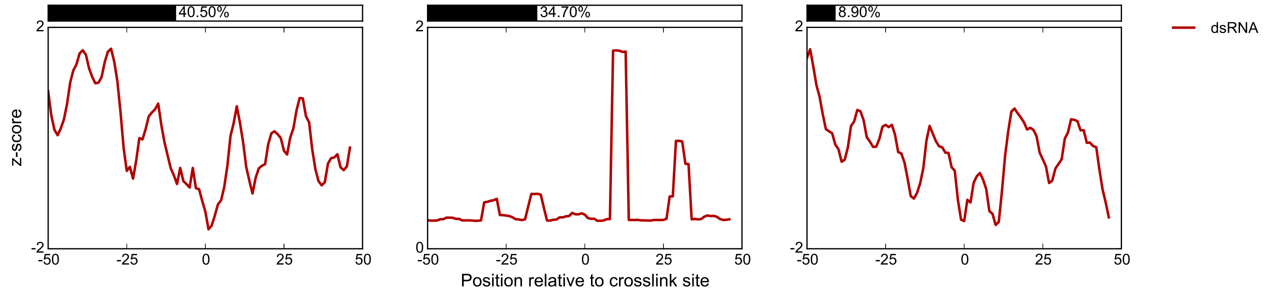

[27] TDP-43 ($\alpha = 10^1$)

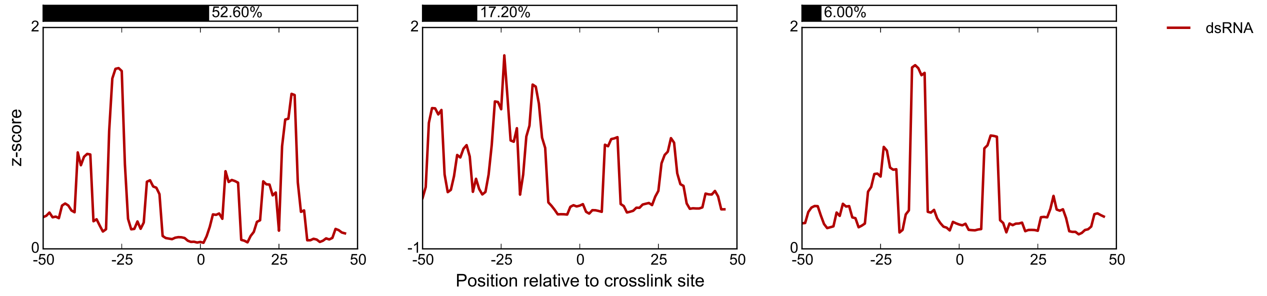

[28] TIA1 ($\alpha = 10^3$)

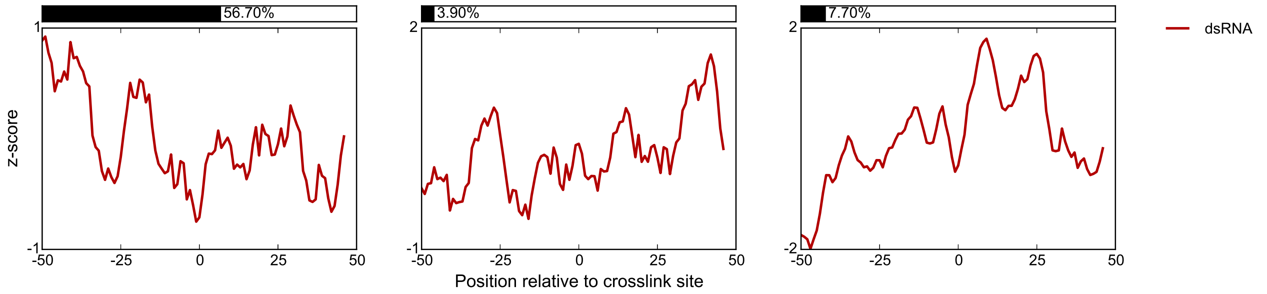

[29] TIAL1 ($\alpha = 10^3$)

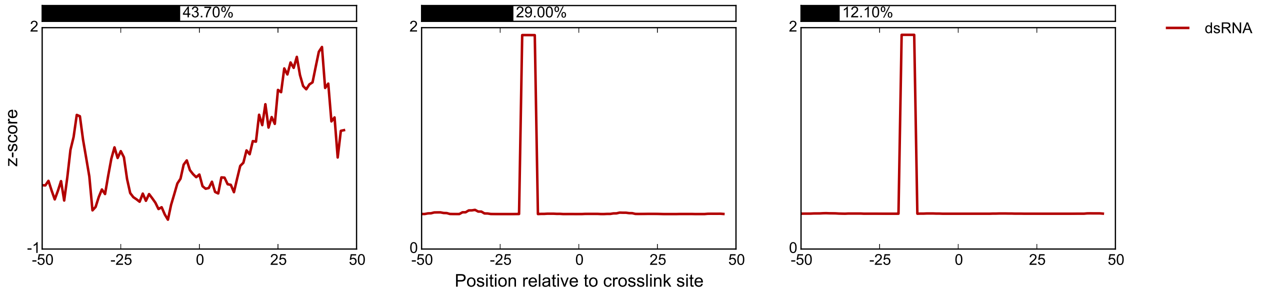

[30] U2AF2 ($\alpha = 10^2$)

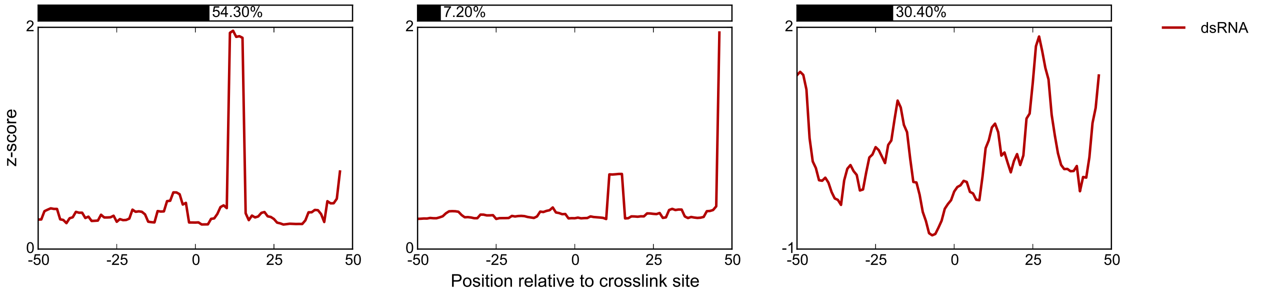

[31] U2AF2 (KD) ($\alpha = 10^{-1}$)

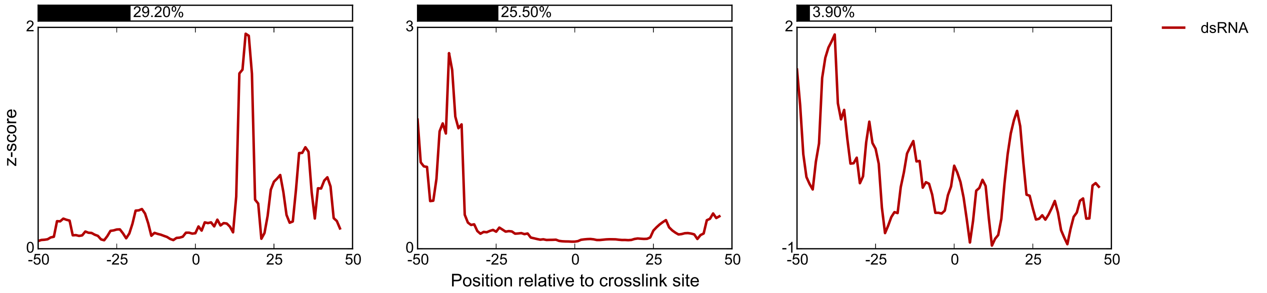

8.4 Experiments (X_{CLIP})

[1] Ago/EIF2C1-4 ($\alpha = 10^4$)

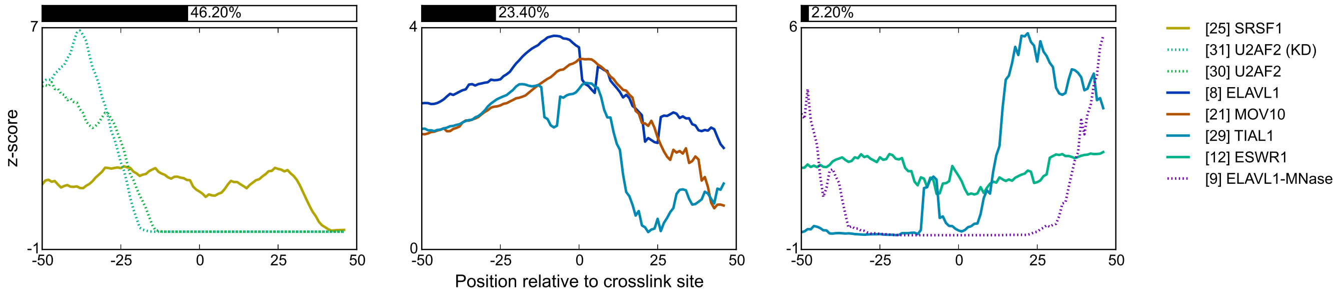

[2] Ago2-MNase ($\alpha = 10^3$)

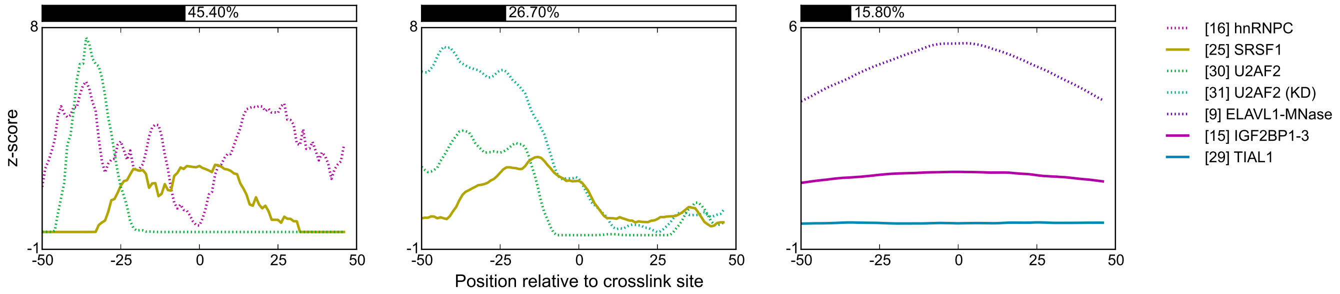

[3] Ago2 (1) ($\alpha = 10^{-1}$)

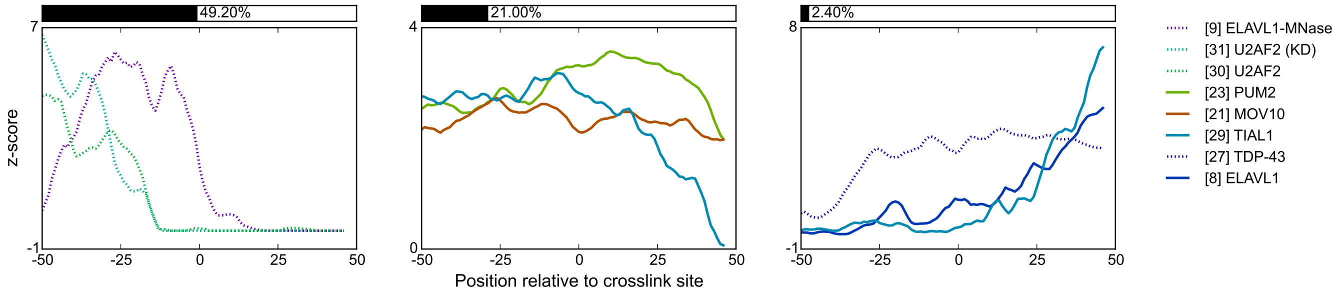

[4] Ago2 (2) ($\alpha = 10^2$)

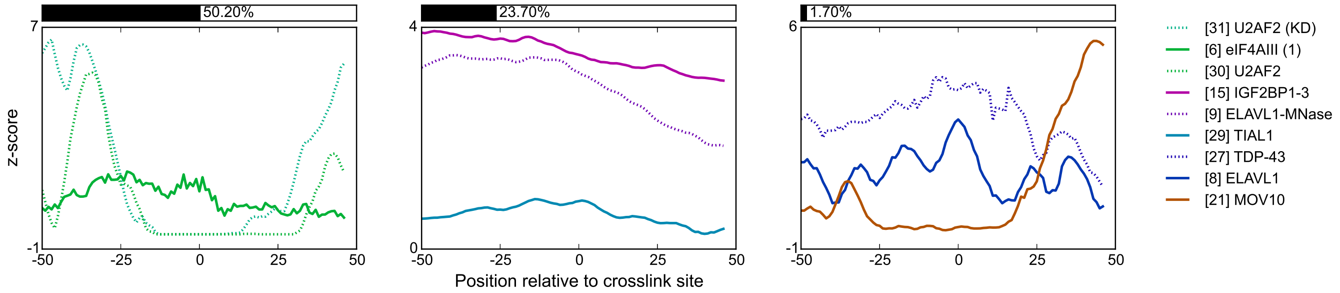

[5] Ago2 ($\alpha = 10^2$)

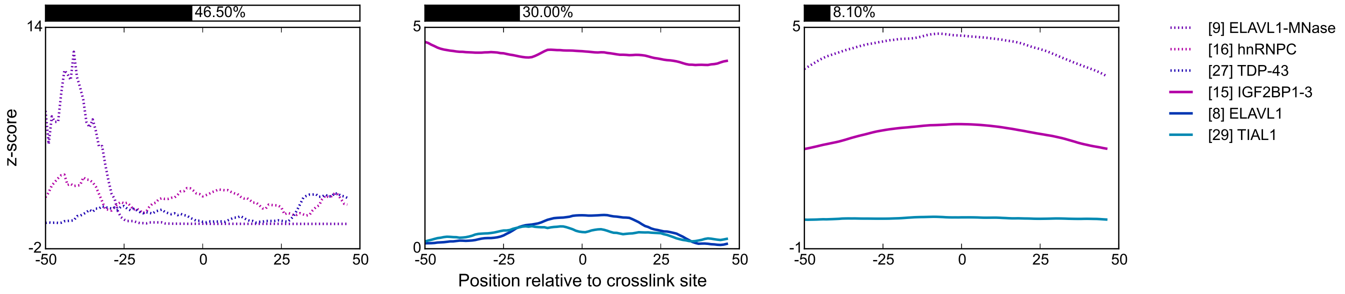

[6] eIF4AIII (1) ($\alpha = 10^3$)

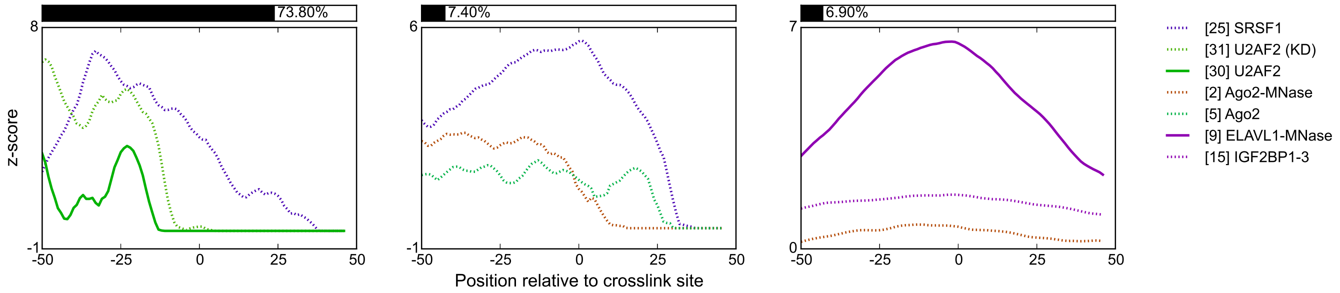

[7] eIF4AIII (2) ($\alpha = 10^2$)

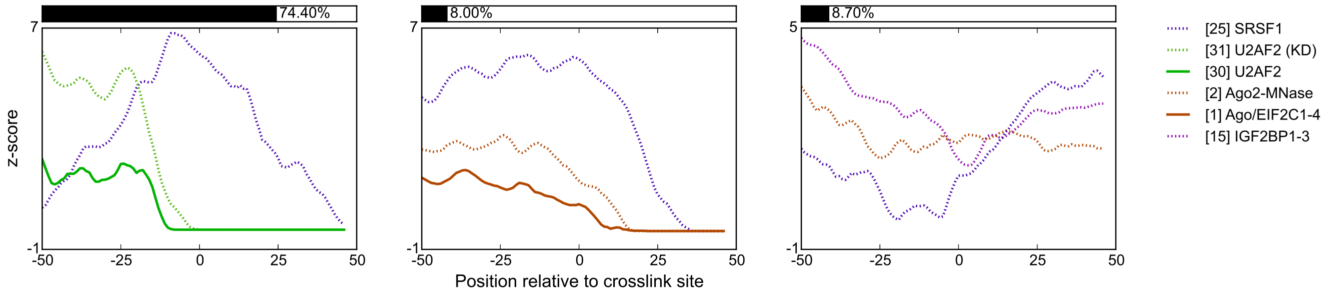

[8] ELAVL1 ($\alpha = 10^2$)

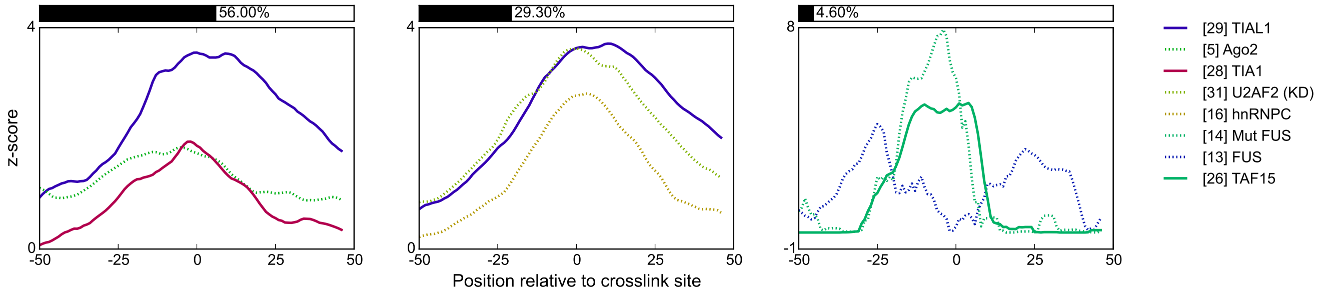

[9] ELAVL1-MNase ($\alpha = 10^0$)

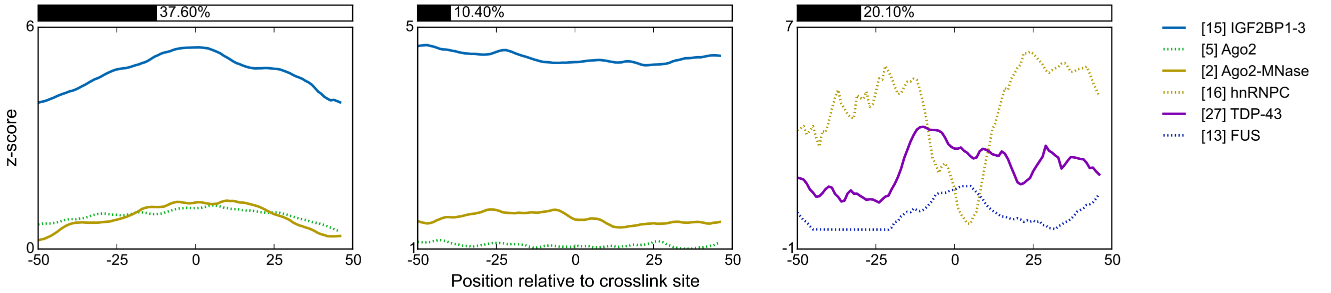

[10] ELAVL1A ($\alpha = 10^3$)

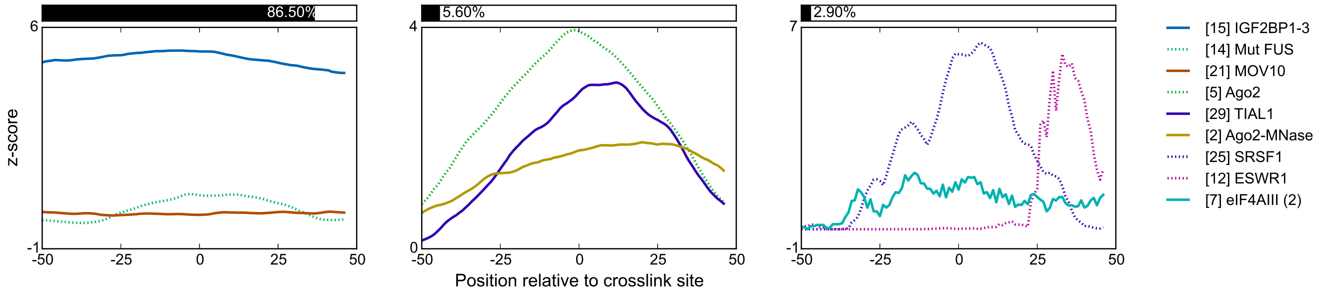

[11] ELAVL1 ($\alpha = 10^2$)

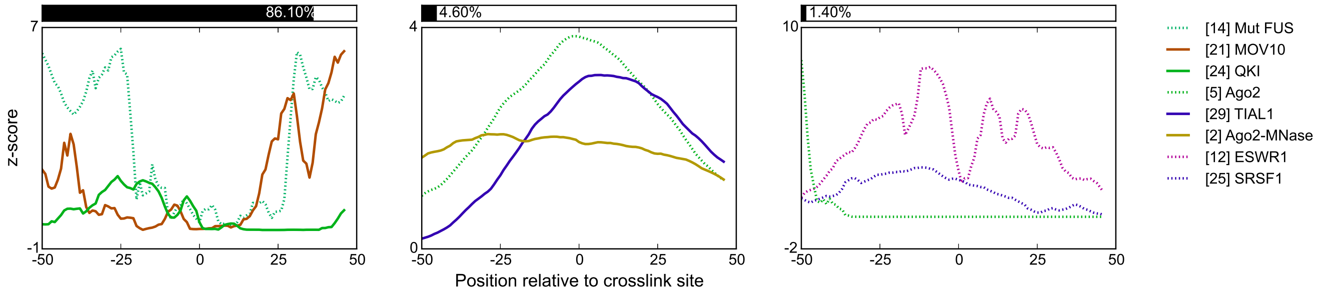

[12] ESWR1 ($\alpha = 10^2$)

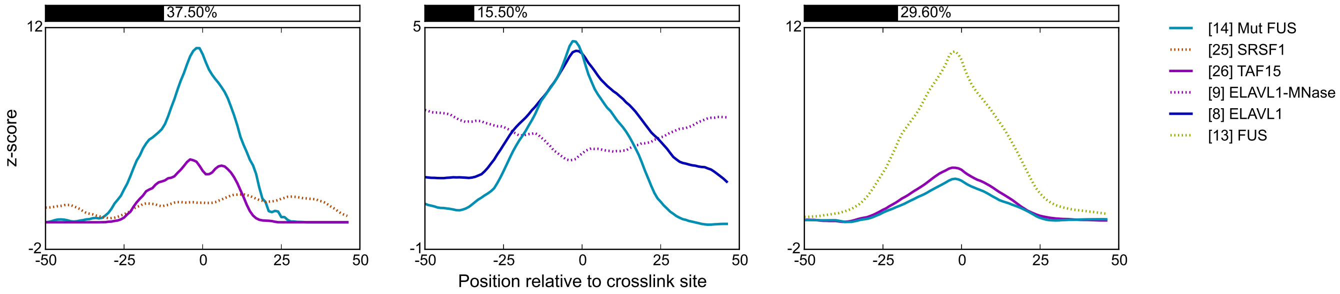

[13] FUS ($\alpha = 10^{-1}$)

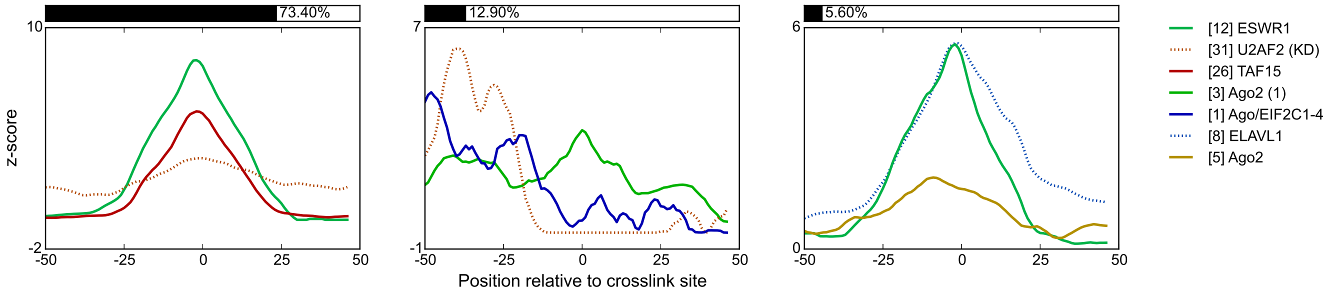

[14] Mut FUS ($\alpha = 10^2$)

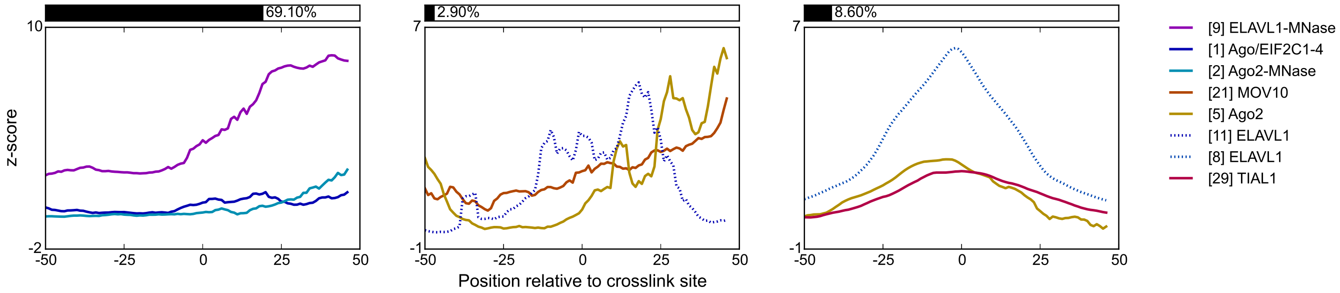

[15] IGF2BP1-3 ($\alpha = 10^2$)

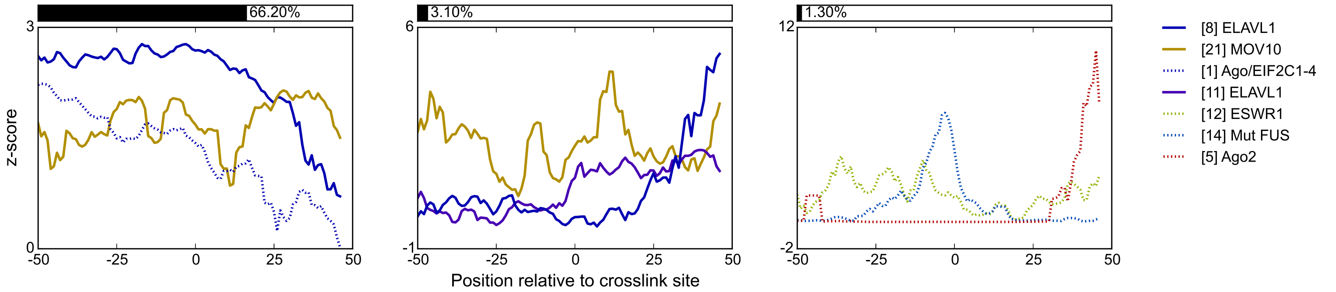

[16] hnRNPC ($\alpha = 10^0$)

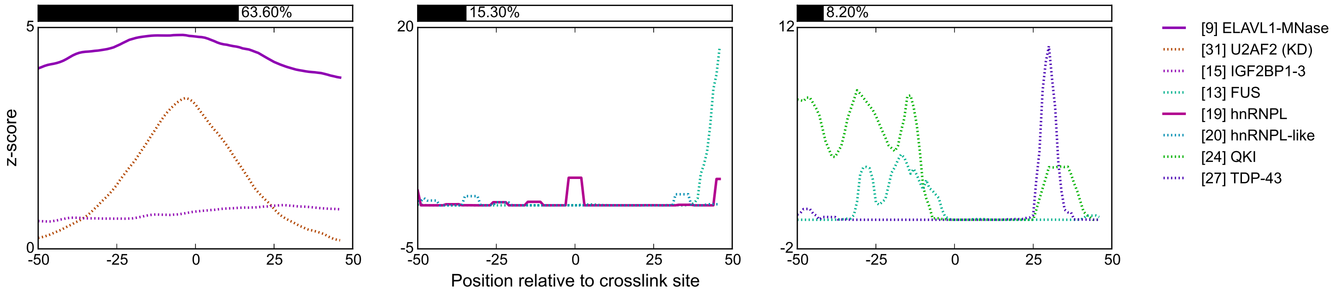

[17] hnRNPC ($\alpha = 10^0$)

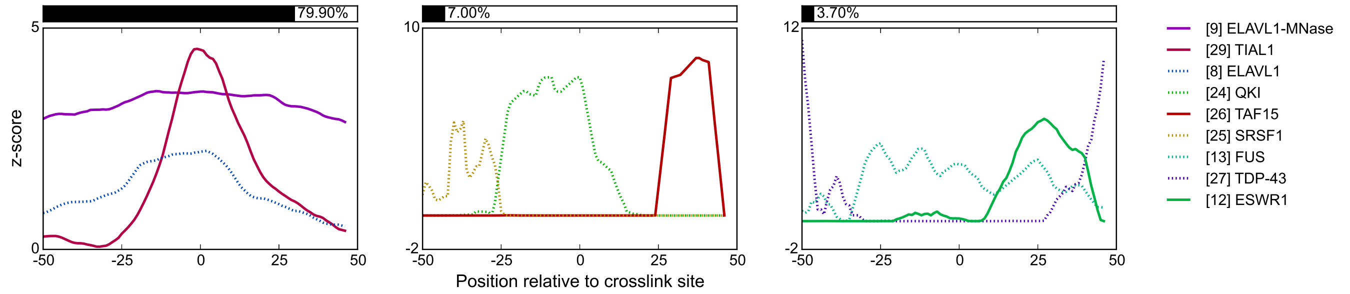

[18] hnRNPL ($\alpha = 10^3$)

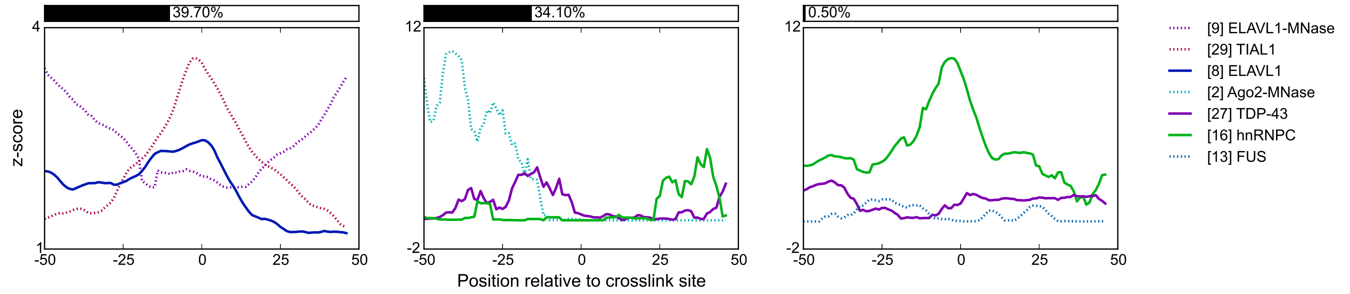

[19] hnRNPL ($\alpha = 10^5$)

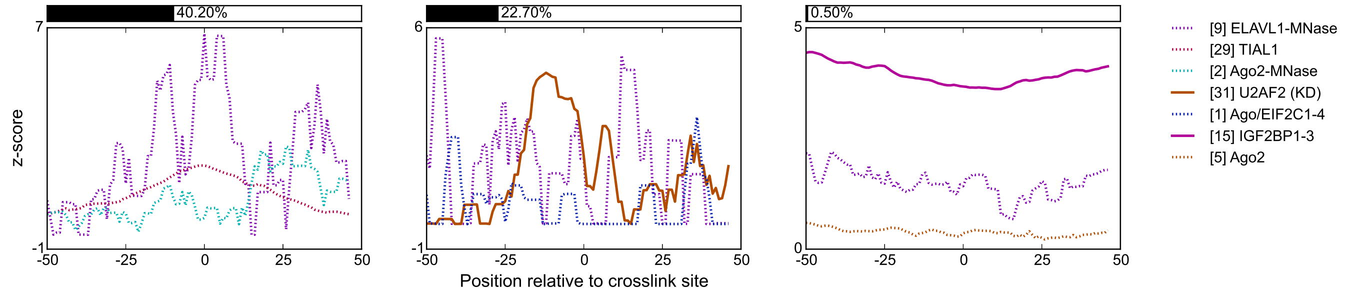

[20] hnRNPL-like ($\alpha = 10^3$)

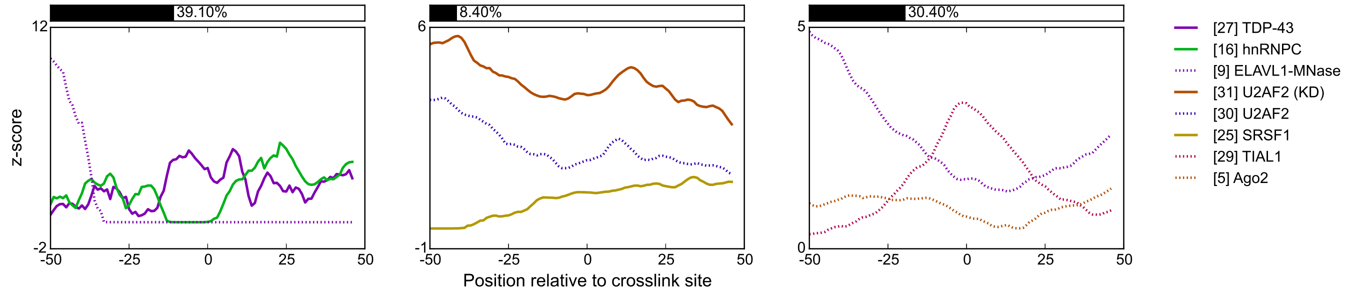

[21] MOV10 ($\alpha = 10^{-1}$)

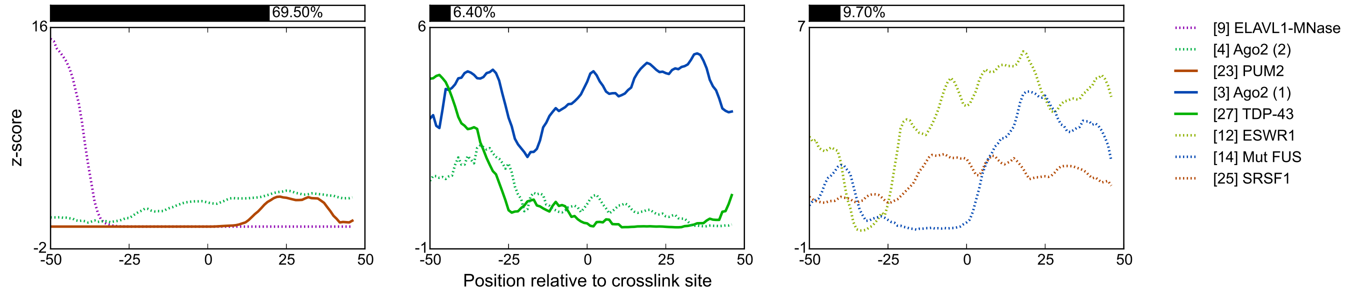

[22] Nsun2 ($\alpha = 10^5$)

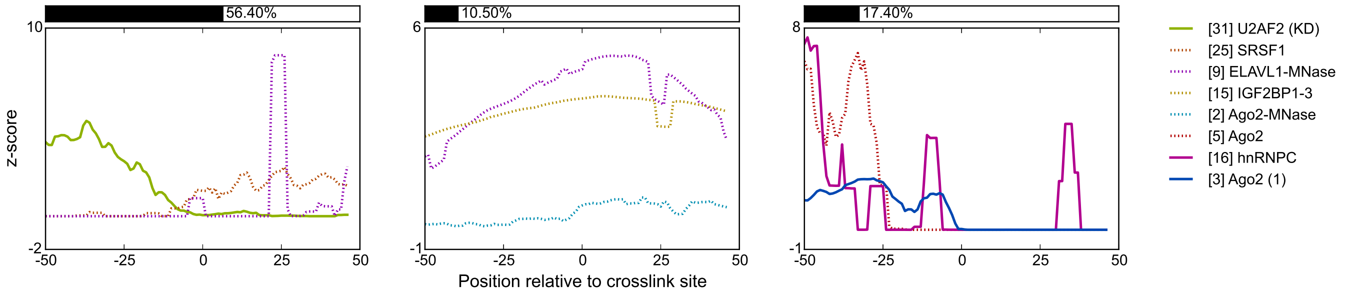

[23] PUM2 ($\alpha = 10^2$)

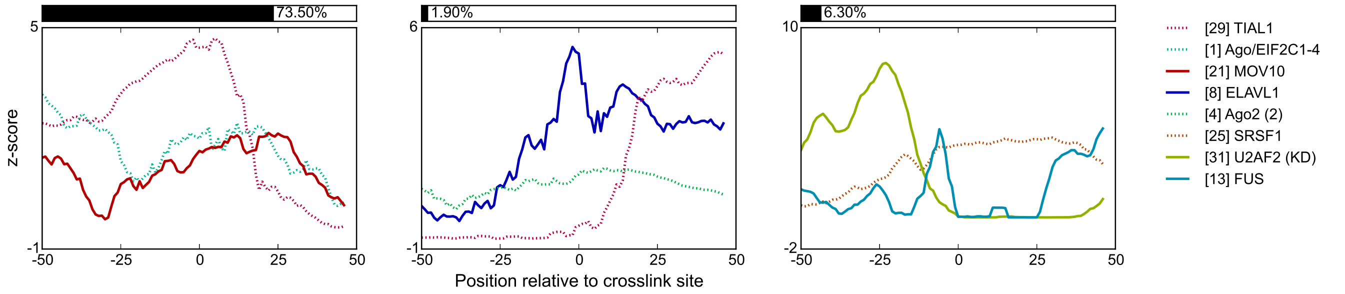

[24] QKI ($\alpha = 10^2$)

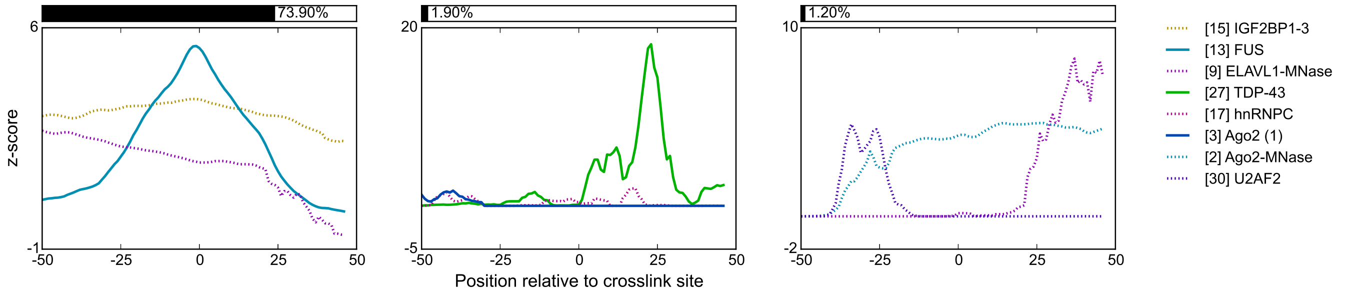

[25] SRSF1 ($\alpha = 10^2$)

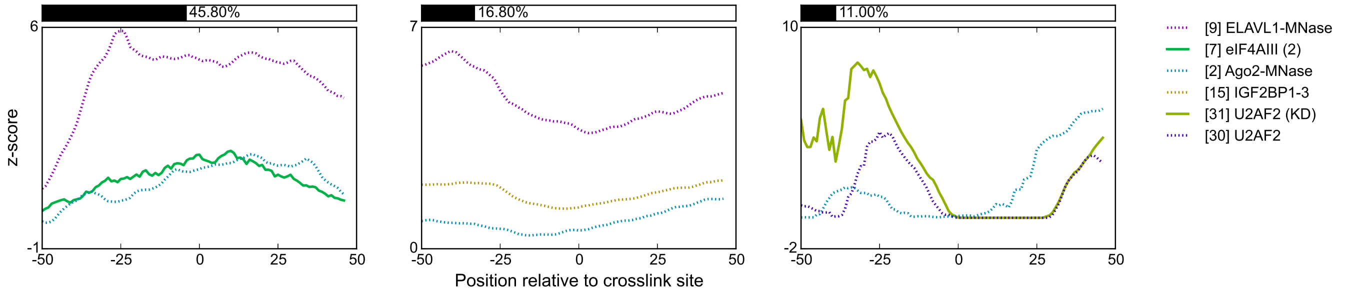

[26] TAF15 ($\alpha = 10^2$)

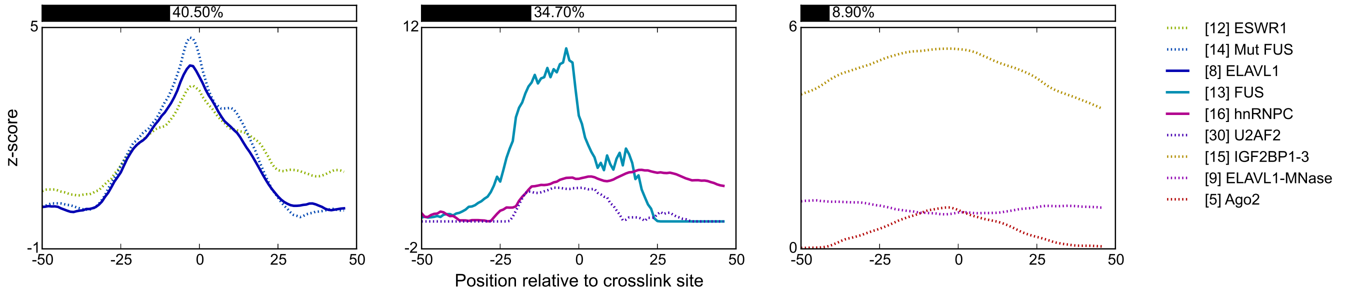

[27] TDP-43 ($\alpha = 10^1$)

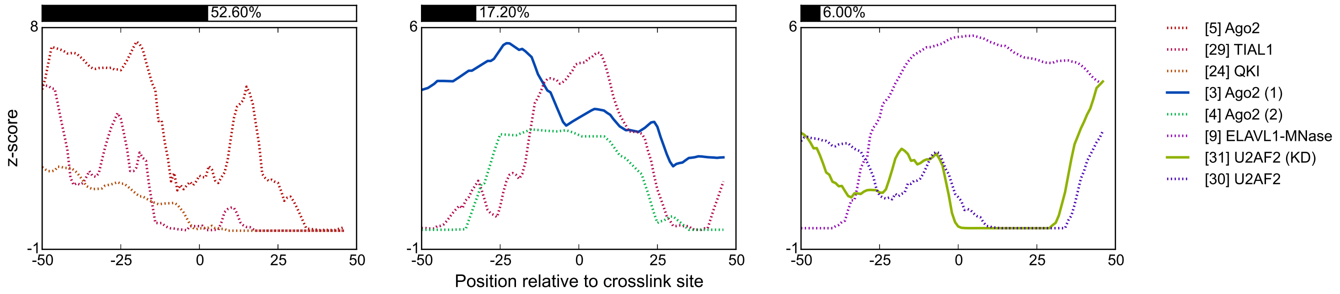

[28] TIA1 ($\alpha = 10^3$)

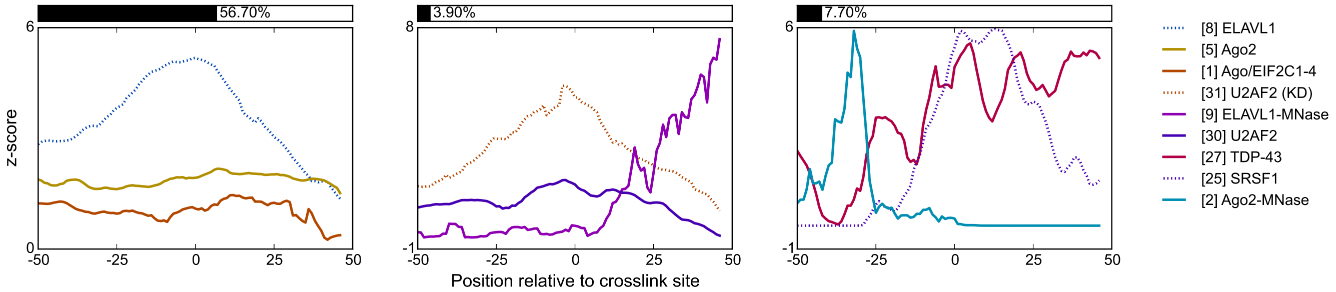

[29] TIAL1 ($\alpha = 10^3$)

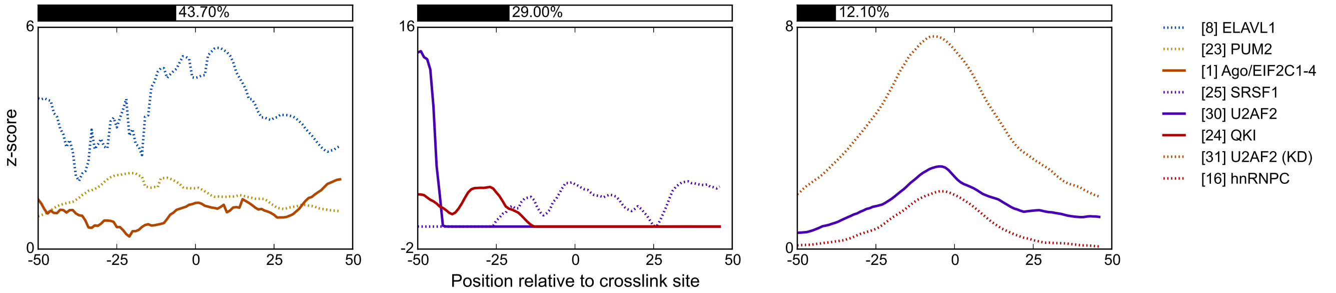

[30] U2AF2 ($\alpha = 10^2$)

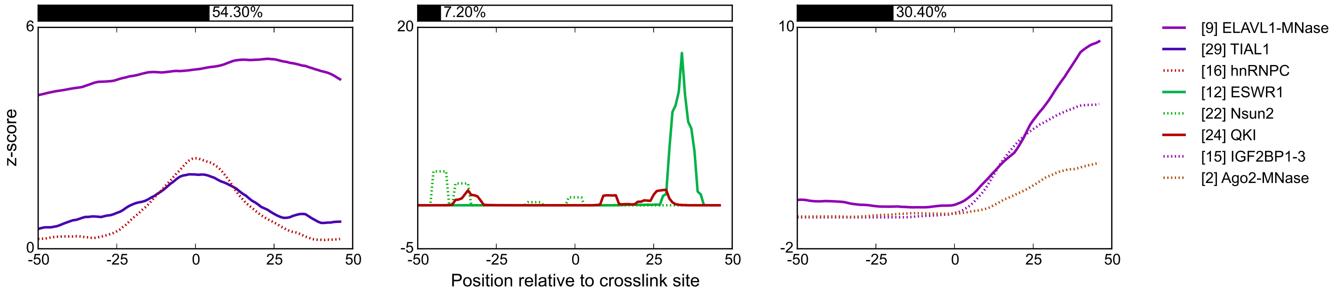

[31] U2AF2 (KD) ($\alpha = 10^{-1}$)

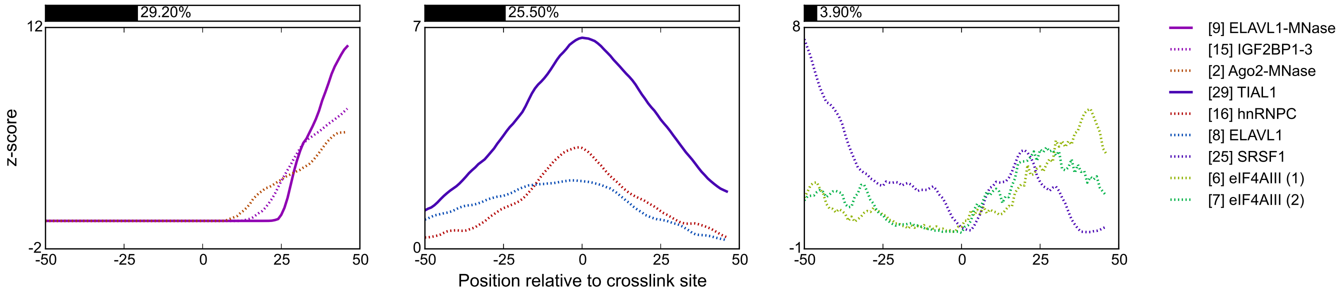

8.5 Gene associations ($\mathbf{H_{GO}}$)

Protein	Term	Module	z-score
[1] Ago/EIF2C1-4	GO:0005515: protein binding	0	77.075
	GO:0005886: plasma membrane	0	60.145
	GO:0003677: DNA binding	0	55.933
	GO:0005737: cytoplasm	0	51.207
	GO:0070062: extracellular vesicular exosome	0	49.568
	GO:0016021: integral component of membrane	1	71.596
	GO:0016020: membrane	1	55.560
	GO:0005634: nucleus	1	54.895
	GO:0044822: poly(A) RNA binding	2	44.502
	GO:0044281: small molecule metabolic process	2	40.813
[2] Ago2-MNase	GO:0070062: extracellular vesicular exosome	0	72.929
	GO:0016020: membrane	0	68.585
	GO:0016021: integral component of membrane	0	59.236
	GO:0005576: extracellular region	0	49.182
	GO:0005886: plasma membrane	0	43.597
	GO:0005737: cytoplasm	1	93.107
	GO:0005634: nucleus	1	73.142
	GO:0005515: protein binding	1	53.963
	GO:0008150: biological_process	1	46.417
	GO:0005654: nucleoplasm	2	37.711
	GO:0043066: negative regulation of apoptotic process	2	35.951
	GO:0003677: DNA binding	2	35.905
	GO:0006355: regulation of transcription, DNA-templated	2	33.608
	GO:0000122: negative regulation of transcription from RNA polymerase II promoter	2	30.941
[3] Ago2 (1)	GO:0016021: integral component of membrane	0	68.692
	GO:0044281: small molecule metabolic process	0	63.496
	GO:0070062: extracellular vesicular exosome	0	55.758
	GO:0005634: nucleus	0	48.881
	GO:0016020: membrane	0	43.997
	GO:0005886: plasma membrane	1	45.056
	GO:0004930: G-protein coupled receptor activity	1	38.928
	GO:0055085: transmembrane transport	1	32.689
	GO:0005515: protein binding	2	112.255
	GO:0005887: integral component of plasma membrane	2	42.661
	GO:0005737: cytoplasm	2	42.402
[4] Ago2 (2)	GO:0005634: nucleus	0	114.855
	GO:0005515: protein binding	0	55.574
	GO:0006351: transcription, DNA-templated	0	50.738
	GO:0016021: integral component of membrane	0	46.070
	GO:0006355: regulation of transcription, DNA-templated	0	43.060
	GO:0005737: cytoplasm	1	74.400
	GO:0005886: plasma membrane	1	73.743
	GO:0005829: cytosol	1	60.417
	GO:0003677: DNA binding	2	47.281

Supplementary Table 10: Gene Ontology terms, modules and z-scores.

Protein	Term	Module	z-score
[5] Ago2	GO:0005789: endoplasmic reticulum membrane	0	67.210
	GO:0008134: transcription factor binding	0	53.965
	GO:0016787: hydrolase activity	0	44.953
	GO:0016568: chromatin modification	0	43.186
	GO:0016575: histone deacetylation	0	37.874
	GO:0005515: protein binding	1	96.456
	GO:0005634: nucleus	1	79.565
	GO:0005737: cytoplasm	1	63.488
	GO:0016021: integral component of membrane	1	51.151
	GO:0005886: plasma membrane	1	49.485
	GO:0003674: molecular_function	2	89.229
	GO:0042692: muscle cell differentiation	2	60.533
	GO:0004842: ubiquitin-protein transferase activity	2	49.561
	GO:0016567: protein ubiquitination	2	45.140
	GO:0008270: zinc ion binding	2	42.228
[6] eIF4AIII (1)	GO:0005515: protein binding	0	86.138
	GO:0005737: cytoplasm	0	66.290
	GO:0016021: integral component of membrane	0	49.745
	GO:0046872: metal ion binding	0	45.442
	GO:0006508: proteolysis	0	40.733
	GO:0005634: nucleus	1	64.103
	GO:0005886: plasma membrane	1	58.316
	GO:0005654: nucleoplasm	2	56.034
	GO:0005615: extracellular space	2	54.789
	GO:0046982: protein heterodimerization activity	2	43.075
	GO:0005887: integral component of plasma membrane	2	41.787
	GO:0006325: chromatin organization	2	38.030
[7] eIF4AIII (2)	GO:0005737: cytoplasm	0	82.691
	GO:0005515: protein binding	0	77.484
	GO:0005886: plasma membrane	0	48.102
	GO:0070062: extracellular vesicular exosome	0	42.820
	GO:0046872: metal ion binding	0	41.573
	GO:0016021: integral component of membrane	1	73.151
	GO:0005634: nucleus	1	44.271
[8] ELAVL1	GO:0005515: protein binding	0	104.294
	GO:0005886: plasma membrane	0	69.770
	GO:0005634: nucleus	0	68.178
	GO:0016021: integral component of membrane	0	65.664
	GO:0005737: cytoplasm	0	58.458
	GO:0046872: metal ion binding	2	50.252
	GO:0005739: mitochondrion	2	42.231
	GO:0006915: apoptotic process	2	36.770
	GO:0006351: transcription, DNA-templated	2	35.397

Supplementary Table 11: Gene Ontology terms, modules and z-scores (continued).

Protein	Term	Module	z-score
[9] ELAVL1-MNase	GO:0016021: integral component of membrane	0	97.512
	GO:0005886: plasma membrane	0	63.696
	GO:0008152: metabolic process	0	38.201
	GO:0005794: Golgi apparatus	0	25.178
	GO:0015031: protein transport	0	24.664
	GO:0005515: protein binding	1	91.793
	GO:0005634: nucleus	1	83.071
	GO:0005737: cytoplasm	1	62.670
[10] ELAVL1A	GO:0005515: protein binding	0	108.075
	GO:0005634: nucleus	0	70.492
	GO:0005737: cytoplasm	0	59.772
	GO:0005886: plasma membrane	0	57.893
	GO:0016021: integral component of membrane	0	53.371
	GO:0005887: integral component of plasma membrane	1	92.088
	GO:0070062: extracellular vesicular exosome	1	50.257
	GO:0007268: synaptic transmission	1	44.181
	GO:0005215: transporter activity	1	42.479
	GO:0046872: metal ion binding	2	57.865
	GO:0006351: transcription, DNA-templated	2	54.741
	GO:0003677: DNA binding	2	49.703
[11] ELAVL1	GO:0005886: plasma membrane	0	97.965
	GO:0016021: integral component of membrane	0	75.231
	GO:0005515: protein binding	0	70.047
	GO:0005634: nucleus	0	49.464
	GO:0005737: cytoplasm	0	40.385
	GO:0016020: membrane	1	69.746
	GO:0005887: integral component of plasma membrane	1	60.813
	GO:0003674: molecular_function	1	47.513
	GO:0005575: cellular_component	1	47.473
	GO:0005615: extracellular space	2	63.931
	GO:0046872: metal ion binding	2	59.304
	GO:0044822: poly(A) RNA binding	2	45.559
	GO:0043547: positive regulation of GTPase activity	2	40.190
[12] ESWR1	GO:0008152: metabolic process	0	56.162
	GO:0016021: integral component of membrane	0	48.494
	GO:0043547: positive regulation of GTPase activity	0	41.376
	GO:0005886: plasma membrane	0	41.270
	GO:0005739: mitochondrion	0	40.938
	GO:0005515: protein binding	1	111.394
	GO:0005634: nucleus	1	76.662
	GO:0005737: cytoplasm	1	65.523
	GO:0030672: synaptic vesicle membrane	2	67.879
	GO:0008021: synaptic vesicle	2	66.696
	GO:0008324: cation transmembrane transporter activity	2	57.959
	GO:0071577: zinc ion transmembrane transport	2	53.026
	GO:0015633: zinc transporting ATPase activity	2	52.415

Supplementary Table 12: Gene Ontology terms, modules and z-scores (continued).

Protein	Term	Module	z-score
[13] FUS	GO:0016021: integral component of membrane	0	101.512
	GO:0005887: integral component of plasma membrane	0	59.167
	GO:0030054: cell junction	0	56.926
	GO:0007155: cell adhesion	0	34.470
	GO:0055085: transmembrane transport	0	32.275
	GO:0005886: plasma membrane	1	53.661
	GO:0005634: nucleus	1	49.929
	GO:0070062: extracellular vesicular exosome	1	44.288
	GO:0046872: metal ion binding	1	38.427
	GO:0005515: protein binding	2	36.118
[14] Mut FUS	GO:0046872: metal ion binding	0	84.447
	GO:0016020: membrane	0	69.206
	GO:0007165: signal transduction	0	64.755
	GO:0005737: cytoplasm	0	51.424
	GO:0006915: apoptotic process	0	45.464
	GO:0005576: extracellular region	1	76.620
	GO:0008150: biological_process	1	75.575
	GO:0016021: integral component of membrane	1	61.124
	GO:0003674: molecular_function	1	38.945
	GO:0016310: phosphorylation	1	34.927
	GO:0005515: protein binding	2	107.827
	GO:0005634: nucleus	2	90.283
	GO:0005886: plasma membrane	2	45.877
	GO:0005829: cytosol	2	39.912
[15] IGF2BP1-3	GO:0016021: integral component of membrane	0	114.720
	GO:0005515: protein binding	0	68.766
	GO:0005886: plasma membrane	0	54.452
	GO:0005829: cytosol	0	50.842
	GO:0070062: extracellular vesicular exosome	0	49.925
	GO:0016020: membrane	1	79.791
	GO:0005509: calcium ion binding	1	45.572
	GO:0046872: metal ion binding	1	41.568
	GO:0005737: cytoplasm	1	37.109
	GO:0005634: nucleus	2	100.397
	GO:0005524: ATP binding	2	31.817
[16] hnRNPC	GO:0016021: integral component of membrane	0	82.823
	GO:0009887: organ morphogenesis	0	32.683
	GO:0030054: cell junction	0	32.478
	GO:0045211: postsynaptic membrane	0	31.327
	GO:0007411: axon guidance	0	30.861
	GO:0005576: extracellular region	1	113.430
	GO:0005575: cellular_component	1	24.747
	GO:0002576: platelet degranulation	1	21.334
	GO:0043198: dendritic shaft	1	18.367
	GO:0000187: activation of MAPK activity	2	48.065
	GO:0005615: extracellular space	2	40.811
	GO:0030141: secretory granule	2	39.817
	GO:0007267: cell-cell signaling	2	38.915

Supplementary Table 13: Gene Ontology terms, modules and z-scores (continued).

Protein	Term	Module	z-score
[17] hnRNPC	GO:0016021: integral component of membrane	0	118.205
	GO:0005783: endoplasmic reticulum	0	34.582
	GO:0005886: plasma membrane	0	33.213
	GO:0007186: G-protein coupled receptor signaling pathway	0	30.764
	GO:0004930: G-protein coupled receptor activity	0	28.010
	GO:0005576: extracellular region	1	69.160
	GO:0007399: nervous system development	1	40.265
	GO:0030054: cell junction	1	38.002
	GO:0031225: anchored component of membrane	1	35.974
	GO:0055085: transmembrane transport	2	61.091
	GO:0044281: small molecule metabolic process	2	48.630
	GO:0005829: cytosol	2	47.062
[18] hnRNPL	GO:0005634: nucleus	0	88.777
	GO:0005737: cytoplasm	0	75.631
	GO:0005515: protein binding	0	75.574
	GO:0016020: membrane	0	49.703
	GO:0005829: cytosol	0	46.632
	GO:0016021: integral component of membrane	1	171.457
	GO:0005887: integral component of plasma membrane	1	44.157
	GO:0005739: mitochondrion	1	43.976
	GO:0007186: G-protein coupled receptor signaling pathway	1	32.572
	GO:0005525: GTP binding	1	27.170
	GO:0050911: detection of chemical stimulus involved in sensory perception of smell	2	74.183
	GO:0004984: olfactory receptor activity	2	62.970
	GO:0003674: molecular_function	2	62.043
	GO:0004930: G-protein coupled receptor activity	2	40.845
[19] hnRNPL	GO:0043234: protein complex	0	80.576
	GO:0007264: small GTPase mediated signal transduction	0	69.778
	GO:0046872: metal ion binding	0	53.504
	GO:0044281: small molecule metabolic process	0	42.670
	GO:0005739: mitochondrion	0	41.949
	GO:0005886: plasma membrane	1	167.380
	GO:0008150: biological_process	1	49.313
	GO:0005794: Golgi apparatus	1	40.909
	GO:0055085: transmembrane transport	1	37.471
	GO:0007186: G-protein coupled receptor signaling pathway	1	32.705
	GO:0016021: integral component of membrane	2	166.905
	GO:0005887: integral component of plasma membrane	2	70.153
	GO:0005509: calcium ion binding	2	35.860
	GO:0003674: molecular_function	2	24.407
[20] hnRNPL-like	GO:0008146: sulfotransferase activity	0	47.583
	GO:0032968: positive regulation of transcription elongation from RNA polymerase II promoter	0	46.821
	GO:0045638: negative regulation of myeloid cell differentiation	0	40.413
	GO:0008150: biological_process	0	35.127
	GO:0071565: nBAF complex	0	31.879
	GO:0007091: metaphase/anaphase transition of mitotic cell cycle	1	43.761
	GO:0060596: mammary placode formation	1	38.246
	GO:0032098: regulation of appetite	1	37.771
	GO:0008299: isoprenoid biosynthetic process	1	32.403
	GO:0016601: Rac protein signal transduction	1	30.525
	GO:0005515: protein binding	2	91.455
	GO:0005634: nucleus	2	89.348
	GO:0005737: cytoplasm	2	71.793
	GO:0016021: integral component of membrane	2	53.390
	GO:0005886: plasma membrane	2	50.505

Supplementary Table 14: Gene Ontology terms, modules and z-scores (continued).

Protein	Term	Module	z-score
[21] MOV10	GO:0005886: plasma membrane	0	100.872
	GO:0016021: integral component of membrane	0	57.765
	GO:0005634: nucleus	0	52.848
	GO:0005515: protein binding	0	50.166
	GO:0005576: extracellular region	0	33.377
	GO:0070062: extracellular vesicular exosome	1	53.077
	GO:0005737: cytoplasm	2	59.889
	GO:0006351: transcription, DNA-templated	2	56.019
	GO:0003677: DNA binding	2	34.837
[22] Nsun2	GO:0005515: protein binding	0	177.262
	GO:0005576: extracellular region	0	45.046
	GO:0005739: mitochondrion	0	37.624
	GO:0001701: in utero embryonic development	0	25.010
	GO:0016020: membrane	0	24.395
	GO:0005634: nucleus	1	121.214
	GO:0005737: cytoplasm	1	97.238
	GO:0005886: plasma membrane	1	60.317
	GO:0006351: transcription, DNA-templated	1	42.172
	GO:0016021: integral component of membrane	1	39.295
	GO:0045944: positive regulation of transcription from RNA polymerase II promoter	2	57.689
	GO:0008270: zinc ion binding	2	57.567
	GO:0005829: cytosol	2	51.262
[23] PUM2	GO:0016021: integral component of membrane	0	104.505
	GO:0005515: protein binding	0	78.901
	GO:0005886: plasma membrane	0	72.916
	GO:0005634: nucleus	0	45.876
	GO:0005829: cytosol	0	36.622
	GO:0016020: membrane	1	71.783
	GO:0005737: cytoplasm	1	67.493
[24] QKI	GO:0016021: integral component of membrane	0	105.330
	GO:0070062: extracellular vesicular exosome	0	80.410
	GO:0005576: extracellular region	0	72.835
	GO:0005515: protein binding	0	59.225
	GO:0005615: extracellular space	0	46.980
	GO:0031512: motile primary cilium	1	47.727
	GO:0005930: axoneme	1	47.572
	GO:0005581: collagen trimer	1	44.589
	GO:0005891: voltage-gated calcium channel complex	1	39.471
	GO:0045880: positive regulation of smoothened signaling pathway	1	38.182
	GO:0005242: inward rectifier potassium channel activity	2	66.494
	GO:0008237: metallopeptidase activity	2	45.909
	GO:0006958: complement activation, classical pathway	2	43.938
	GO:0018149: peptide cross-linking	2	43.116

Supplementary Table 15: Gene Ontology terms, modules and z-scores (continued).

Protein	Term	Module	z-score
[25] SRSF1	GO:0005515: protein binding	0	88.929
	GO:0005634: nucleus	0	71.631
	GO:0005886: plasma membrane	0	64.521
	GO:0005737: cytoplasm	0	60.743
	GO:0016021: integral component of membrane	0	51.526
	GO:0003674: molecular_function	2	40.899
	GO:0007186: G-protein coupled receptor signaling pathway	2	38.157
	GO:0004930: G-protein coupled receptor activity	2	29.785
	GO:0050911: detection of chemical stimulus involved in sensory perception of smell	2	25.774
[26] TAF15	GO:0005515: protein binding	0	84.202
	GO:0016021: integral component of membrane	0	83.297
	GO:0005634: nucleus	0	75.860
	GO:0005886: plasma membrane	0	61.955
	GO:0070062: extracellular vesicular exosome	0	47.794
	GO:0044281: small molecule metabolic process	1	79.358
	GO:0005743: mitochondrial inner membrane	1	53.461
	GO:0003824: catalytic activity	1	45.080
	GO:0007268: synaptic transmission	1	33.809
	GO:0030203: glycosaminoglycan metabolic process	1	30.629
	GO:0005737: cytoplasm	2	69.312
	GO:0008270: zinc ion binding	2	38.725
	GO:0003674: molecular_function	2	35.756
[27] TDP-43	GO:0006766: vitamin metabolic process	0	77.188
	GO:0006767: water-soluble vitamin metabolic process	0	77.085
	GO:0032324: molybdopterin cofactor biosynthetic process	0	68.944
	GO:0006777: Mo-molybdopterin cofactor biosynthetic process	0	62.723
	GO:0051539: 4 iron, 4 sulfur cluster binding	0	58.392
	GO:0016021: integral component of membrane	1	85.692
	GO:0031625: ubiquitin protein ligase binding	1	35.546
	GO:0023035: CD40 signaling pathway	1	34.419
	GO:0000209: protein polyubiquitination	1	34.214
	GO:0035631: CD40 receptor complex	1	34.057
	GO:0003674: molecular_function	2	67.879
	GO:0071797: LUBAC complex	2	48.589
	GO:0045211: postsynaptic membrane	2	47.205
[28] TIA1	GO:0005515: protein binding	0	108.598
	GO:0016021: integral component of membrane	0	80.368
	GO:0005737: cytoplasm	0	67.533
	GO:0005886: plasma membrane	0	66.893
	GO:0070062: extracellular vesicular exosome	0	48.539
	GO:0005634: nucleus	1	128.608
	GO:0006351: transcription, DNA-templated	1	51.522
	GO:0003676: nucleic acid binding	1	35.871
	GO:0008270: zinc ion binding	1	35.594
	GO:0005829: cytosol	1	32.837

Supplementary Table 16: Gene Ontology terms, modules and z-scores (continued).

Protein	Term	Module	z-score
[29] TIAL1	GO:0005515: protein binding	0	101.379
	GO:0005634: nucleus	0	78.523
	GO:0005737: cytoplasm	0	74.823
	GO:0005886: plasma membrane	0	53.732
	GO:0016020: membrane	0	43.364
	GO:0006351: transcription, DNA-templated	1	50.429
	GO:0003677: DNA binding	1	42.686
	GO:0005829: cytosol	1	40.065
	GO:0016021: integral component of membrane	2	102.536
	GO:0005783: endoplasmic reticulum	2	33.579
	GO:0042803: protein homodimerization activity	2	32.274
	GO:0042127: regulation of cell proliferation	2	31.910
	GO:0005794: Golgi apparatus	2	31.725
[30] U2AF2	GO:0016021: integral component of membrane	0	100.698
	GO:0005783: endoplasmic reticulum	0	61.477
	GO:0005789: endoplasmic reticulum membrane	0	60.172
	GO:0005575: cellular_component	0	48.037
	GO:0003674: molecular_function	0	45.775
	GO:0005615: extracellular space	1	37.947
	GO:0007186: G-protein coupled receptor signaling pathway	1	36.486
	GO:0005578: proteinaceous extracellular matrix	1	31.276
	GO:0005581: collagen trimer	1	22.699
	GO:0005515: protein binding	2	105.051
	GO:0005634: nucleus	2	79.353
	GO:0005737: cytoplasm	2	60.386
	GO:0005886: plasma membrane	2	50.441
[31] U2AF2 (KD)	GO:0016021: integral component of membrane	0	117.715
	GO:0046872: metal ion binding	0	57.454
	GO:0055085: transmembrane transport	0	42.999
	GO:0005576: extracellular region	0	37.210
	GO:0030198: extracellular matrix organization	0	34.364
	GO:0008140: cAMP response element binding protein binding	1	39.260
	GO:0030176: integral component of endoplasmic reticulum membrane	1	39.030
	GO:0005789: endoplasmic reticulum membrane	1	37.815
	GO:0005783: endoplasmic reticulum	1	36.159
	GO:0005886: plasma membrane	2	76.026
	GO:0005887: integral component of plasma membrane	2	57.721
	GO:0005739: mitochondrion	2	54.135
	GO:0008152: metabolic process	2	44.507

Supplementary Table 17: Gene Ontology terms, modules and z-scores (continued).

References

Aenkoe, M.-L., *et al.* (2012). The RNA-binding landscapes of two SR proteins reveal unique functions and binding to diverse RNA classes. *Genome Biol.*, **13**(3), R17.

Boudreau, R. L., *et al.* (2014). Transcriptome-wide Discovery of microRNA Binding Sites in Human Brain. *Neuron*, **81**(2), 294–305.

Chatterjee, S., *et al.* (2015). *Regression analysis by example*. John Wiley & Sons.

Hafner, M., *et al.* (2010). Transcriptome-wide identification of RNA-binding protein and microRNA target sites by PAR-CLIP. *Cell*, **141**(1), 129–41.

Hoell, J. I., *et al.* (2011). RNA targets of wild-type and mutant FET family proteins. *Nat. Struct. Mol. Biol.*, **18**(12), 1428–1431.

Hussain, S., *et al.* (2013). NSun2-mediated cytosine-5 methylation of vault noncoding RNA determines its processing into regulatory small RNAs. *Cell Reports*, **4**(2), 255–61.

Hutchins, L. N., *et al.* (2008). Position-dependent motif characterization using non-negative matrix factorization. *Bioinformatics*, **24**(23), 2684–90.

Kazan, H., *et al.* (2010). RNAcontext: a new method for learning the sequence and structure binding preferences of RNA-binding proteins. *PLoS Comput. Biol.*, **6**(7).

Kishore, S., *et al.* (2011). A quantitative analysis of CLIP methods for identifying binding sites of RNA-binding proteins. *Nat. Methods*, **8**(7), 559–64.

König, J., *et al.* (2010). iCLIP reveals the function of hnRNP particles in splicing at individual nucleotide resolution. *Nat. Struct. Mol. Biol.*, **17**(7), 909–15.

Lee, D. D. D., *et al.* (2001). Algorithms for Non-negative Matrix Factorization. *Advances in NIPS*, (1), 548–562.

Lin, C.-J. (2007). Projected gradient methods for nonnegative matrix factorization. *Neural Comput.*, **19**(10), 2756–79.

Maticzka, D., *et al.* (2014). GraphProt: modeling binding preferences of RNA-binding proteins. *Genome Biol.*, **15**(1), R17.

Ray, D., *et al.* (2013). A compendium of RNA-binding motifs for decoding gene regulation. *Nature*, **499**(7457), 172–7.

Rossbach, O., *et al.* (2014). Crosslinking-immunoprecipitation (iCLIP) analysis reveals global regulatory roles of hnRNP L. *RNA Biol.*, **11**(2), 146–155.

Salakhutdinov, R., *et al.* (2008). Bayesian probabilistic matrix factorization using Markov chain Monte Carlo. *Proceedings of the 25th international conference on Machine learning - ICML '08*, pages 880–887.

Sanford, J. R., *et al.* (2009). Splicing factor SFRS1 recognizes a functionally diverse landscape of RNA transcripts. *Genome Res.*, **19**(3), 381–394.

Saulière, J., *et al.* (2012). CLIP-seq of eIF4AIII reveals transcriptome-wide mapping of the human exon junction complex. *Nat. Struct. Mol. Biol.*, **19**(11), 1124–1131.

Sievers, C., *et al.* (2012). Mixture models and wavelet transforms reveal high confidence RNA-protein interaction sites in MOV10 PAR-CLIP data. *Nucleic Acids Res.*, **40**(20), e160—-e160.

Tollervey, J. R., *et al.* (2011). Characterizing the RNA targets and position-dependent splicing regulation by TDP-43. *Nat. Neurosci.*, **14**(4), 452–U180.

Uren, P. J., *et al.* (2011). Genomic Analyses of the RNA-binding Protein Hu Antigen R (HuR) Identify a Complex Network of Target Genes and Novel Characteristics of Its Binding Sites. *J. Biol. Chem.*, **286**(43), 37063–37066.

Wang, Z., *et al.* (2010). iCLIP Predicts the Dual Splicing Effects of TIA-RNA Interactions. *PLoS Biol.*, **8**(10), e1000530.

Zarnack, K., *et al.* (2013). Direct competition between hnRNP C and U2AF65 protects the transcriptome from the exonization of Alu elements. *Cell*, **152**(3), 453–466.

Zdunek, R., *et al.* (2006). Non-negative matrix factorization with quasi-newton optimization. *Artificial Intelligence and Soft Computing*, pages 870–879.
